# The Mars Environmental Dynamics Analyzer, MEDA. A Suite of Environmental Sensors for the Mars 2020 Mission

**DOI:** 10.1007/s11214-021-00816-9

**Published:** 2021-04-13

**Authors:** J. A. Rodriguez-Manfredi, M. de la Torre Juárez, A. Alonso, V. Apéstigue, I. Arruego, T. Atienza, D. Banfield, J. Boland, M. A. Carrera, L. Castañer, J. Ceballos, H. Chen-Chen, A. Cobos, P. G. Conrad, E. Cordoba, T. del Río-Gaztelurrutia, A. de Vicente-Retortillo, M. Domínguez-Pumar, S. Espejo, A. G. Fairen, A. Fernández-Palma, R. Ferrándiz, F. Ferri, E. Fischer, A. García-Manchado, M. García-Villadangos, M. Genzer, S. Giménez, J. Gómez-Elvira, F. Gómez, S. D. Guzewich, A.-M. Harri, C. D. Hernández, M. Hieta, R. Hueso, I. Jaakonaho, J. J. Jiménez, V. Jiménez, A. Larman, R. Leiter, A. Lepinette, M. T. Lemmon, G. López, S. N. Madsen, T. Mäkinen, M. Marín, J. Martín-Soler, G. Martínez, A. Molina, L. Mora-Sotomayor, J. F. Moreno-Álvarez, S. Navarro, C. E. Newman, C. Ortega, M. C. Parrondo, V. Peinado, A. Peña, I. Pérez-Grande, S. Pérez-Hoyos, J. Pla-García, J. Polkko, M. Postigo, O. Prieto-Ballesteros, S. C. R. Rafkin, M. Ramos, M. I. Richardson, J. Romeral, C. Romero, K. D. Runyon, A. Saiz-Lopez, A. Sánchez-Lavega, I. Sard, J. T. Schofield, E. Sebastian, M. D. Smith, R. J. Sullivan, L. K. Tamppari, A. D. Thompson, D. Toledo, F. Torrero, J. Torres, R. Urquí, T. Velasco, D. Viúdez-Moreiras, S. Zurita, The MEDA team

**Affiliations:** 1 Centro de Astrobiología (INTA-CSIC), Madrid, Spain; 2 Jet Propulsion Laboratory/California Institute of Technology, Pasadena, CA USA; 3 CRISA-Airbus, Tres Cantos, Spain; 4 Instituto Nacional de Técnica Aeroespacial (INTA), Madrid, Spain; 5 Universidad Politécnica de Cataluña, Barcelona, Spain; 6 Cornell Center for Astrophysics and Planetary Science, Cornell University, Ithaca, NY USA; 7 Added-Value-Solutions, Elgoibar, Spain; 8 Instituto de Microelectrónica de Sevilla (US-CSIC), Seville, Spain; 9 Universidad del País Vasco (UPV/EHU), Bilbao, Spain; 10 Carnegie Institution, Washington, DC USA; 11 Università degli Studi di Padova, Padova, Italy; 12 University of Michigan, Ann Arbor, MI USA; 13 Finnish Meteorological Institute, Helsinki, Finland; 14 Space Science Institute, Boulder, CO USA; 15 Lunar and Planetary Institute, Houston, TX USA; 16 Aeolis Corporation, Sierra Madre, CA USA; 17 Universidad Politécnica de Madrid, Madrid, Spain; 18 Southwest Research Institute, Boulder, CO USA; 19 Universidad de Alcalá, Alcalá de Henares, Spain; 20 John Hopkins APL, Laurel, MD USA; 21 Dept. of Atmospheric Chemistry and Climate, Institute of Physical Chemistry Rocasolano, CSIC, Madrid, Spain; 22 NASA Goddard Space Flight Center, Greenbelt, MD USA

**Keywords:** MEDA instrument, Mars2020, Perseverance, Instruments, Mars, Atmosphere, Pressure, Wind, Temperature, Surface temperature, Albedo, Dust, Clouds, UV, Thermal infrared, Radiation fluxes

## Abstract

NASA’s Mars 2020 (M2020) rover mission includes a suite of sensors to monitor current environmental conditions near the surface of Mars and to constrain bulk aerosol properties from changes in atmospheric radiation at the surface. The Mars Environmental Dynamics Analyzer (MEDA) consists of a set of meteorological sensors including wind sensor, a barometer, a relative humidity sensor, a set of 5 thermocouples to measure atmospheric temperature at ∼1.5 m and ∼0.5 m above the surface, a set of thermopiles to characterize the thermal IR brightness temperatures of the surface and the lower atmosphere. MEDA adds a radiation and dust sensor to monitor the optical atmospheric properties that can be used to infer bulk aerosol physical properties such as particle size distribution, non-sphericity, and concentration. The MEDA package and its scientific purpose are described in this document as well as how it responded to the calibration tests and how it helps prepare for the human exploration of Mars. A comparison is also presented to previous environmental monitoring payloads landed on Mars on the Viking, Pathfinder, Phoenix, MSL, and InSight spacecraft.

## Introduction

The missions sent to Mars during the last decades have significantly increased the level of knowledge and understanding of the processes and environmental dynamics of our neighboring planet. The Martian atmosphere is intrinsically different from the Earth atmosphere, and provides an example of the diversity of environments that can be found on different planets (Petrosyan et al. 2011).

The near surface remains one of the least understood regions of the Martian atmosphere. At the time of this writing several stations have landed on a range of locations on Mars providing information on a subset of Martian environments and only Viking, Curiosity and InSight sampled their environment beyond one Martian season. In parallel, Mars orbiting instruments have provided a global picture of the composition and dynamics of the free atmosphere. However, data recorded from orbit lack the vertical resolution necessary to discriminate near-surface atmospheric processes from those occurring within the lowest few kilometers of the atmosphere (McCleese et al. 2007) or to analyze the local surface properties at the small horizontal scales achievable with a landed instrument, especially in those processes of interest where a small resolution scale is required. This is particularly important in preparing for future human exploration missions on Mars, where an accurate understanding of those processes is crucial for the design of mission assets as well as the safe landing of astronauts and their survival on the surface in this hostile and challenging Martian environment.

The Mars 2020 mission has four primary objectives: explore Jezero Crater as an astrobiologically relevant ancient environment on Mars, to decipher its geological processes and history, including the assessment of the past habitability conditions; assess the biosignature preservation potential and search for potential biosignatures on the selected geological environments; take relevant samples from those environments, in preparation for the future mission to return them to Earth; and prepare for human exploration, by demonstrating significant technical progress compatible with the science payloads. The preparation for human exploration includes the characterization of the environment following the MEPAG recommendations (MEPAG 2014):

*Contribute to the preparation for human exploration of Mars by making significant progress towards filling at least one major Strategic Knowledge Gap (SKG).*

The highest priority SKG measurements that are synergistic with Mars 2020 science objectives, and compatible with the mission concept are (in priority order):

1. Demonstration of In-Situ Resource Utilization (ISRU) technologies to enable propellant and consumable oxygen production from the Martian atmosphere for future exploration missions.
2. Characterization of atmospheric dust size and morphology to understand its effects on the operation of surface systems and human health. (Mission Goal D1)
3. Surface weather measurements to validate global atmospheric models. (Mission Goal D2)

The Mars Environmental Dynamics Analyzer, MEDA, was selected to satisfy Mission Goals D1 and D2. MEDA additionally informs the environmental context in which M2020 samples have been collected and preserved on the Martian surface.

MEDA will help with Goal D1, which includes characterizing the dust environment during and between operations of the MOXIE (Mars Oxygen In-Situ Resource Utilization Experiment) instrument.

Aerobraking, entry-descent-landing, ascent, and surface human operations are all sensitive to the state of the atmosphere, yet the MEPAG Goals Document notes that “We do not have sufficient Martian atmospheric observations [of the lower atmosphere] to confidently model winds, which significantly affect EDL design...” and that “atmospheric models for Mars have not been well validated due to a lack of sufficient observational data, and thus our confidence in them (for use in mission engineering) is significantly limited”. For this reason, M2020 Goal D3 is to make “Surface weather measurements to validate global atmospheric models” and MEDA sensors will provide significant advances here compared to previous missions: a greater measurement capability, more precision and accuracy, more sensors and new variables. Also, in combination with the other two working stations currently in operation (REMS in Gale Crater and TWINS in Elysium Planitia), the three systems will be used to help further validate and constrain global circulation models and parameterizations therein.

Additionally, MEDA supports the overall science and sampling mission by providing environmental context. M2020’s goal C1 is to “Obtain samples that are scientifically selected, for which the field context is documented...” with atmospheric conditions forming part of the context in which the samples were acquired. While a sample return mission is being planned for the mid-2020s, the samples may wait for retrieval for longer than that if plans change. It will be important to understand the full environmental conditions, particularly the relatively humidity, as trapped water vapor may influence the sample over time. Thus, MEDA will document the atmospheric environment that was present at the time the sample was collected and which may be sealed in with the sample.

MEDA measurements will also address key science objectives noted by the Mars Exploration Program Analysis Group in their Goals Document (MEPAG 2014). Goal II is to “Understand the processes and history of the climate on Mars” and MEPAG has defined the objectives, sub-objectives, and investigations that would further this goal for present-day Mars. Specific investigations that drove the definition of the sensors requirements are given in each sensor’s section. In general the MEPAG document cites among the high-priority measurements winds measured simultaneously with temperature and pressure, which MEDA will provide. MEDA will also constrain the surface energy balance, needed to understand local surface exchange processes and atmospheric forcing from the ground. Dust lifting processes can be improved via the combination of surface measurements by associated Mars 2020 cameras, MEDA surface net heating measurements, and atmospheric pressure, temperature, wind, and dust loading measurements. The instrument provides observations on diurnal to yearly (and perhaps longer) timescales that are needed to understand processes such as dust events and atmospheric or surface condensation and the potential for sub-surface exchange of water. The instrument also provides new information on regional influences of atmospheric behavior from conducting measurements in a location on Mars not previously visited. Finally, the return of atmospheric samples with the cached geologic samples can aid in understanding the trace gas content and its relationship to surface chemistry and any trapped dust or aerosols can be examined for potential substrates upon which heterogeneous chemistry could occur.

MEDA is an evolution of the environmental suite (the REMS instrument) and cameras (HazCam) on the Mars Science Laboratory (MSL) and Mars Exploration Rover (MER) missions, as well as the wind sensors on InSight, TWINS. Based on the experience gained and the lessons learned, this new generation of sensors on M2020 has been designed to characterize more completely the local micrometeorology and microclimatology near the surface and to measure how the bulk atmospheric aerosol properties affect the solar radiation observed at the surface. Those changes in solar radiation at the surface are used by MEDA’s camera to infer the atmospheric aerosol properties. The main science objectives of MEDA are thus to characterize both the forcing and response of the near-surface atmosphere.

To carry out the aforementioned investigations, MEDA has been designed as a set of separate sensors, each of them accommodated in the most suitable position possible, within rover constraints. The sensors are listed below and will be discussed in the following sections:

- Air Temperature Sensor (ATS)
- Pressure Sensor (PS)
- Radiation and Dust Sensor (RDS), including SkyCam
- Relative Humidity Sensor (HS)
- Thermal Infrared Sensor (TIRS)
- Wind Sensor (WS)

## Air Temperature Sensor (ATS)

### ATS Science Objectives and Requirements

MEDA’s ATS will characterize the near surface air temperatures at two heights at the locations visited by the rover. Being on a landed rover, it has the capability to measure local thermal processes that cannot be resolved from orbital instruments. The measurements will constrain models of the development and preservation of near-surface biological activity, the current conditions for the samples that will be cached, the physics of atmospheric processes at the surface, and provide information for design requirements for human exploration. MEDA measurements also help us place in context the thermodynamics of processes that occur on other worlds, and thereby how different our own planet might have been.

Several missions have measured the near surface temperature of the Martian atmosphere. The Viking landers, Mars Pathfinder, Phoenix, Curiosity and InSight all carried thermometers (Martínez et al. 2017). As on Earth, and for its Martian predecessors, the MEDA ATS will face two tough challenges. The first relates to the intrinsic atmospheric variability, whereby the temperature at a given location may be different from the surrounding air parcels a few meters away, which raises the question of how generally representative the air parcel being measured is. The second relates to the degree to which the measured air parcel is affected by the temperature of the atmospheric station hardware itself, in this case the M2020 rover, with its own radiative emissions, heaters and perturbed airflow. The goal is to provide measurements as accurate as possible at the ATS sensor locations, and to interpret those values in the context of everything else occurring in the environment and near the rover so that the values can be used as representative temperatures. Both the accuracy and resolution of the MEDA ATS are sufficient to be able to resolve temperature fluctuations typical of the physical processes of interest. These processes span diurnal and seasonal scales and include estimating the local radiative balance and other heat transfer processes (Incropera and Dewit 1985). The MEDA observations at Jezero will be compared to those at previous mission locations, thereby addressing one of the MEPAG goals of exploring as many different locations as possible on Mars.

Many processes contribute to changing the air temperature at global and local scales. While an orbiter’s strength is in providing global coverage (Kleinböhl et al. 2009), a surface station allows measurement of many local phenomena that are not accessible to orbital sensors. The diurnal temperature signature permits estimates of the local radiative energy budget (Martinez et al. 2014), which informs models of the heat exchange between the atmosphere and surface. This then affects the local hydrological cycle, for example, the possibility of frost formation at night and whether or not regolith adsorption/desorption might occur (Savijärvi et al. 2020, e.g.). Temperature changes are also caused by winds transporting different air masses across the region. These winds in turn are driven by seasonal cycles, thermal tides, and topography. The vertical temperature structure near the surface is indicative of the stability of the atmosphere and, with it, the time-spectral properties of atmospheric winds (e.g. Mahrt 2014; Panofsky 1974, for reviews). Air temperatures also respond to changes in cloud cover (de la Torre-Juarez et al. 2019) and eddies that can develop into dust devils (Ellehøj et al. 2010; Newman et al. 2019; Ordoñez-Etxeberría et al. 2018).

The MEDA ATS is designed to minimize the impact of rover perturbations. It consists of three sensors distributed azimuthally around the rover to be able to correct for rover influences, as well as 2 sensors on the sides of the rover body to get a constraint on the vertical temperature gradient near the surface. Combined with two channels on the MEDA Thermal Infrared Sensor that measure the surface brightness temperature and the air temperature of a layer of air emitting in a thermal infrared window with a weighting function centered at ∼40 m above the surface, MEDA will be able to provide vertical temperature profiles at the surface (0 m), 0.84 m, 1.45 m and ∼40 m. This combination will enable characterization of the air temperature vertical profile, and how it changes as the atmosphere transitions from a nighttime stable profile to unstable daytime convection. The profile within this vertical range from the surface affects horizontal and vertical winds, temperature oscillations (e.g. Geiger 1957; Largeron et al. 2013; Miller et al. 2018; Panofsky 1974; Sorbjan et al. 2009) and near surface turbulence (e.g. Tillman et al. 1994; Davy et al. 2010; Banfield et al. 2020), all of which need to be understood to improve the representation of the lower boundary conditions in Mars atmospheric models (Savijärvi 2011).

To achieve an understanding of the physical processes that leave their thermal signatures in the air, to characterize the near-surface environment for Human exploration and for other M2020 rover investigations, with what is technologically possible for a Mars mission payload, there were several requirements set on the ATS. These requirements are outlined in Table 1.

**Table 1:** ATS requirements

Investigation	Requirements	Performance
Air temperature at 1.5 m altitude above the local surface	MEDA’s Air Temperature Sensor (ATS) shall characterize the thermal environment at the surface of Mars, by taking measurements at least once per hour.	The ATS is a passive unit able to record measurements whenever prompted by the ICU.
	The ATS shall have a temperature range of [150 K to 300 K] locally, at the sensor input.	ATS components have an operating range of at least 73 K–1023 K
	The ATS resolution shall be equal or better than [0.1 K] locally, at the sensor input.	Resolution better than ±0.01 K
	The ATS accuracy shall be equal or better than [±1 K] locally, at the sensor input.	Including effects of control electronics, error is <±1 K
	The ATS response time shall be equal or better than [1 sec]	Manufacturer data sheets specify 0.04s in air. Taylor et al. (2008) measured <0.77 s with no wind.
	ATS shall allow recording the readings at a programmable sampling rate with a maximum of [2 Hz]	ATS signals are continuous (analog) and meet the 2 Hz sampling rate.
	MEDA’s ATS shall be placed at least 5 cm away from all vertical rover surfaces.	Met by design of rover, sensor, and its accommodation location.

### ATS Design and Description

#### Measurement Principle

MEDA’s ATS uses a set of thermocouple sensors. Each of them consists of thin wires of two dissimilar metals joined together at one end, the hot junction, and with the other end of the wire fixed with an electrical insulator to an aluminum support, or cold junction. So, the hot junction is exposed to the air. Any temperature difference between the cold and the hot junctions produces a small thermoelectric voltage difference that is measured and used to determine the temperature at the hot junction by means of several transfer functions. The temperature of the cold junction located in the aluminum block is considered the reference temperature for each unit, and is measured with a PT1000 thermistor on each of them.

#### Mechanical and Thermal Design

**Fig. 1:**
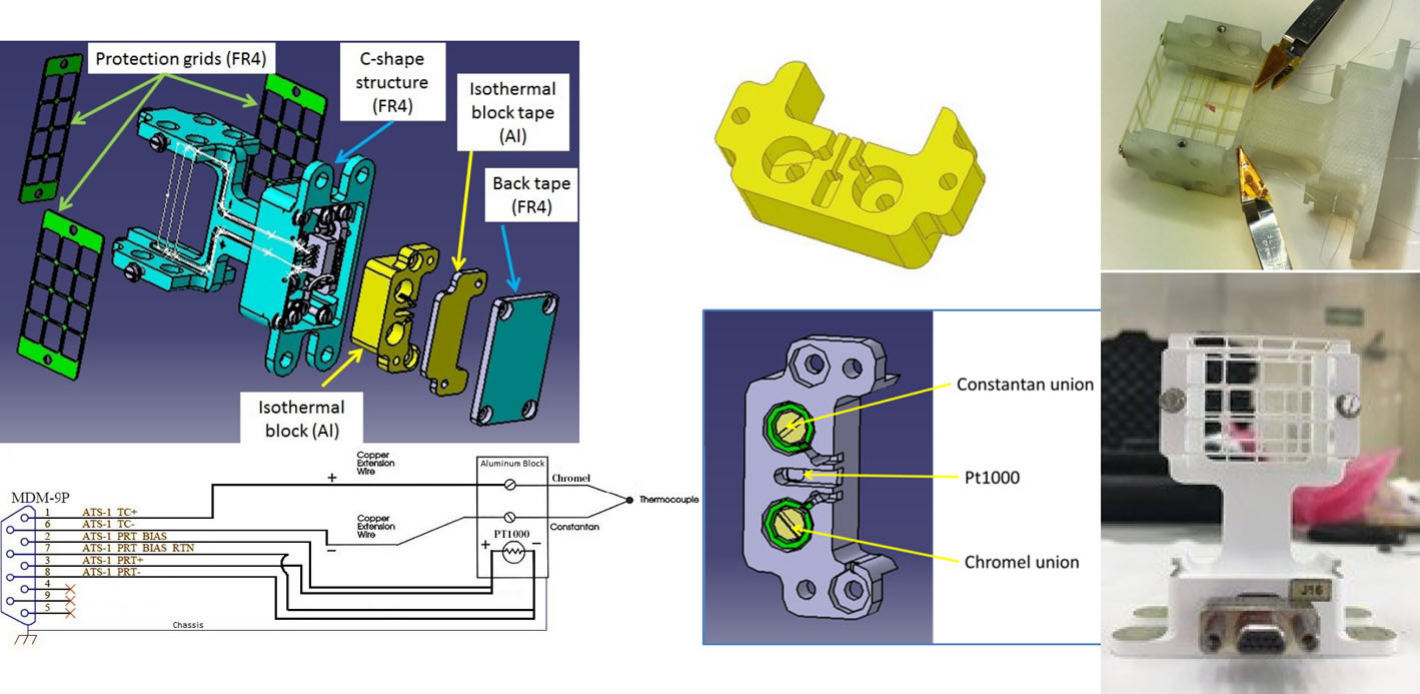
(Left) Elements of each Temperature Sensor TS (top) and TS Circuit Diagram (bottom). (Center) Aluminum isothermal block (top) and components of each isothermal block (bottom). (Right) ATS Three thermocouples glued to the FR4 structure (top) and finished TS unit (bottom)

The MEDA thin-wire thermocouples are made of Constantan/Chromel (E-type) 0.075 mm diameter with a butt-welded junction. Three thermocouples are glued to a C-shaped FR4 structure, with the hot junction suspended mid-way between the arms of the C. The details of the components are shown in Fig. 1.

The reference junctions connect the three thermocouple wires to copper wires via two alumina pieces shown in Fig. 1 (center), and housed in an isothermal block of aluminum (cold junction) in the base support of the ATS, as seen in Fig. 1 (left). The location of the PT1000 thermistor that provides the reference temperature is shown in Fig. 1 (center).

The ATS Circuit Diagram is in the bottom left of Fig. 1. The exposed thermocouple wires are protected against Entry Descent and Landing (EDL) debris impacts by means of a grid seen in the top right of the figure. Signals from the ATS are routed through the harnesses to be digitized inside the MEDA Instrument Control Unit (ICU) by means of a dedicated analog acquisition chain.

#### Accommodation

**Fig. 2:**
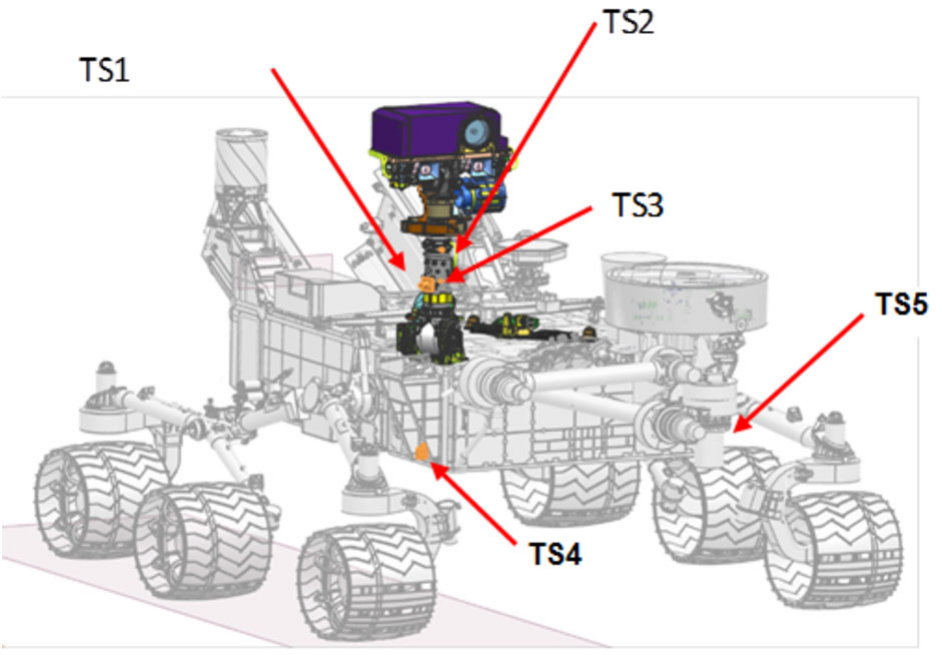
Location of the ATS units on the rover

The location of the 5 Thermocouple Sensors (TS) that form MEDA’s Air temperature is shown in Fig. 2: Three are located at 150 cm above the surface and around the rover’s Remote Sensing Mast (RSM), and two are located on the vertical surface of the rover front body at 70 cm above the surface.

The ATS horizontal distribution around the RSM is driven by the need to identify and minimize thermal influences from the rover surfaces by ensuring that at least 1 unit measures temperatures from air parcels that have not been exposed to the RSM. To minimize the influence from direct solar illumination, the air temperature at 1.5 m altitude above the surface will be defined as the coldest of the three readings from TS1, TS2, or TS3 assuming that the rover is hotter than the air temperature far away the rover. This method of having several thermocouples and select the colder of the readings is similar to the one used in radiosondes on Earth where several thermometers are used, and those exposed directly to solar radiation will measure a warmer temperature than those exposed to the shade. Nevertheless, this strategy can result in nonphysical jumps in temperature readings as it switches from one sensor to another. It will be re-evaluated once Perseverance lands on Mars, and the data generated by the ATS can be processed together and correlatively with the wind, also recorded by the MEDA’s WS.

### ATS Operation

**Fig. 3:**
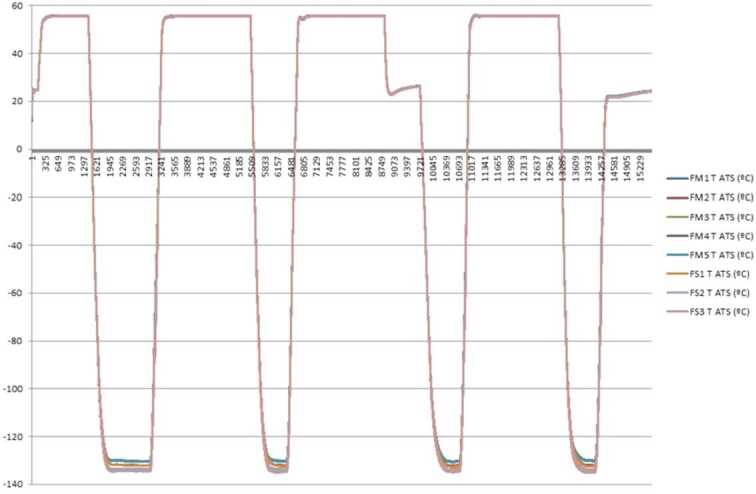
Temperatures measured during the Thermal Vacuum Test in a chamber – Range from 133 K to 328 K for 127 hours

The ATS will collect readings at up to 2 Hz frequency for as long as it is commanded to do so by the ICU. The sampling times and frequency are programmable to meet the requirements of specific investigations proposed by the M2020 operations team. All ATS units have been tested and calibrated to be able to operate properly in the full range of temperatures expected on the surface of Mars as shown in Fig. 3.

### ATS Temporal Response

**Fig. 4:**
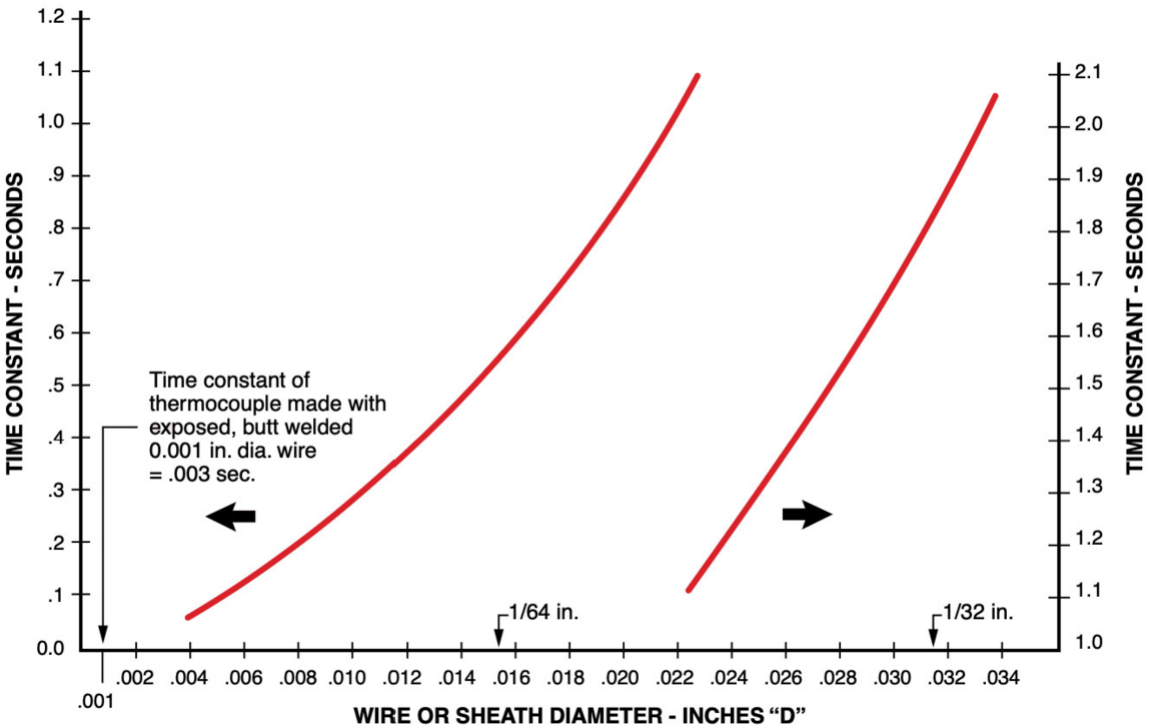
Thermocouple time response in air with a velocity of 19,8 m/s (65 feet/s). Further information is available in vendor’s website (Omega 2021)

The ATS probes use the same thermocouple wires as those on the Phoenix’s (PHX) lander. Taylor et al. (2008) characterized the Phoenix air temperature sensors under Martian atmospheric conditions as having a time constant at wind speeds greater than 5 m/s of less than 0.5 s, and at zero wind speed or less than 0.77 s, as shown in Fig. 4. Thus, the MEDA ATS exceeds the temporal response requirement of better than 1 second under all Martian conditions.

### ATS Thermal Analysis

Thermal analysis was performed to verify that the ATS fulfills the environmental requirements of the mission and to assess its thermal behavior (Peinado et al. 2017). Two worst case scenarios, hot (WHC) and cold (WCC), were analyzed in order to cover the most extreme conditions for both TS3 (RSM mounted) and TS5 (chassis mounted) taking into account in particular the thermal interface with the rover. While it is outside the scope of this document to present the full results of those analyses, we do summarize the WHC and WCC steady state analysis results that could have the most effect on the sensor performance. Tables 2 and 3 show the effects of the predicted temperature gradients in the isothermal block between the PT1000 and the two cold junction locations. We considered both free convection in calm conditions, and forced convection assuming a frontal wind speed of 15 m/s. In all cases, the ATS performances are barely dependent of the isothermal block temperature determined by the PT1000 measurement: the accuracy is affected in 0.2 ^∘^C in the WHC in free regime, while it is not affected in the WCC.

**Table 2:** Temperature gradient for TS3

Gradient	WHC free (^∘^C)	WHC forced (^∘^C)	WCC free (^∘^C)	WCC forced (^∘^C)
PT1000-Cold Junction Chromel	0.2	0.1	0.0	0.0
PT1000-Cold Junction Constantan	0.2	0.1	0.0	0.0

**Table 3:** Temperature gradient for TS5

Gradient	WHC free (^∘^C)	WHC forced (^∘^C)	WCC free (^∘^C)	WCC forced (^∘^C)
PT1000-Cold Junction Chromel	0.1	−0.1	0.0	0.0
PT1000-Cold Junction Constantan	0.1	−0.1	0.0	0.0

### ATS Calibration Results and Transfer Functions

**Fig. 5:**
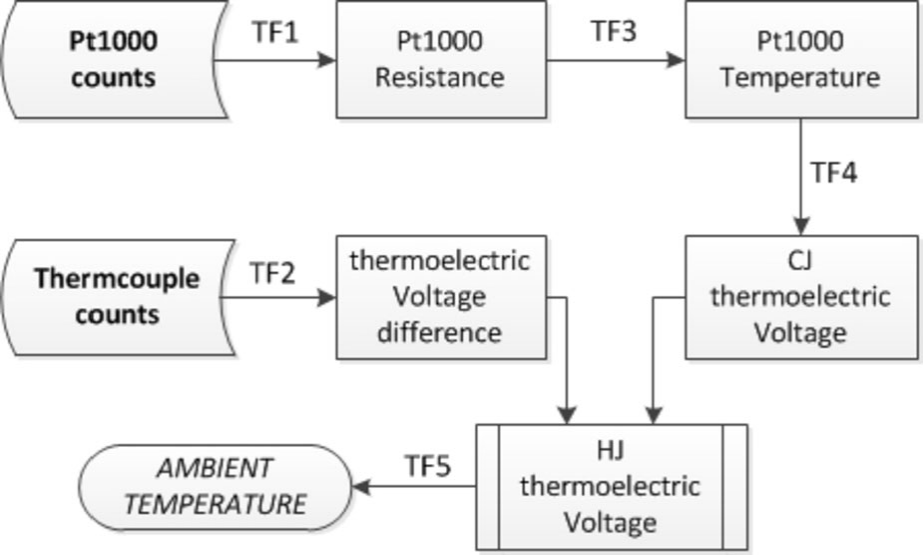
Flow diagram of the retrieval process followed to calculate ambient temperatures from raw counts readings of each TS

The ATS requires two readings to determine the ambient temperature, as summarized in Fig. 5. The hardware provides both readings in “counts”: the PT1000 counts are converted via a transfer function TF1 into a PT1000 resistance that provides the cold junction (CJ) temperature, T_1_ after using a transfer function TF3; and the thermocouple counts are converted via the transfer function TF2 into the thermoelectric Voltage difference (tV_*d*_) between the hot and the cold junction. An equivalent thermoelectric voltage for the CJ (tV_1_) may be assigned to the temperature T_1_ with help of transfer function TF4. The ambient temperature is obtained after combining this reference thermoelectric voltage with the hot junction into tV_2_=tV_1_+tV_*d*_ to infer the ambient temperature after applying a last transfer function TF5.

The calibration of an ATS unit consisted of defining the specific transfer functions that characterize the response of the hardware corresponding to the channels of the ICU, the PT1000 and the 3 redundant thermocouples. The accuracy of the calibrated thermocouple and related electronics is better than 0.25 ^∘^C for both of them. The accuracy of the standard Pt1000 depends on its working temperature (from 0.4 ^∘^C at -123^∘^C to 0.15 ^∘^C at 0 ^∘^C), thus the accuracy of the whole chain (TS and electronics) is better than 0.9 ^∘^C in the worst case, to less than 0.65 ^∘^C at 0 ^∘^C.

#### Transfer Functions

1. The ICU transfer function TF1, to convert the PT1000’s raw data (counts) to resistance (Ohm). This mathematical expression is the same for all ATS channels (ATS_ATS#_PRT).
  Input range for raw data (counts): [6713; 53732]
  Output range for resistance (Ohm): [432.01; 1144.01]
  $$ R = \frac{ \frac{5*(\mathit{Raw}\_\mathit{data})}{65536} +1.6644}{0.0050382645} $$
2. The ICU transfer function TF2, to convert the thermocouple’s raw data (counts) to thermoelectric voltage difference (in micro Volt, μV). Likewise, it is the same for all ATS channels (ATS_ATS#).
  Input range for raw data (counts): [13690; 59016]
  Output range for thermoelectric voltage difference (micro Volt): [-3000; 1200]
  $$ tV_{d} = \frac{5*(\mathit{Raw}\_\mathit{data}) - 232279.5}{54.607872} $$
3. The transfer function TF3, to convert the resistances of the PT1000s (Ohm) to temperature (^∘^C). This is an interpolated function of the inverse of the standard function to convert temperature to resistance of a PT1000 Class A (3850 ppm).
  The expression for the interpolated function depends on the temperature range defined. For the calibration and acceptance tests, a temperature range larger than that one expected on the surface of Mars was used.
  So, the transfer function estimated for the thermal range anticipated for this Mars mission, i.e. between -125 ^∘^C and +40 ^∘^C, is defined as:
$T_{1} = 1.1348346\cdot 10^{-5}\cdot R^{2} + 2.3327711\cdot 10^{-1} \cdot R - 2.4465296\cdot 10^{2}$
4. The transfer function TF4, obtained by calibration to calculate the thermoelectric voltage for a known temperature, such as the temperature given by the PT1000. It is given by:
  tV$_{1} = 2.10413\cdot 10^{-11} \cdot T_{1}^{6} + 5.16133\cdot 10^{-9} \cdot T_{1}^{5} + 2.25473\cdot 10^{-7} \cdot T_{1}^{4} - 1.18513 \cdot 10^{-4} \cdot T_{1}^{3} + 5.13118\cdot 10^{-2}\cdot T_{1}^{2} + 58.1291 \cdot T_{1} + 1.57731 $
5. The transfer function TF5, to convert the thermoelectric voltage, tV_2_, in $\mu $V, to temperature (^∘^C). It is an interpolated function of the previous one. As in the case of the PT1000, the expression of the interpolated function also depends on the temperature range definition for the thermocouple, being defined in the range -125 ^∘^C to +40 ^∘^C. It is given by:
  $T_{2} = -4.65708\cdot 10^{-23} \cdot tV_{2}^{6} - 2.80126\cdot 10^{-19} \cdot tV_{2}^{5} - 1.26304\cdot 10^{-15} \cdot tV_{2}^{4} + 2.13656 \cdot 10^{-11} \cdot tV_{2}^{3} - 2.60200\cdot 10^{-7} \cdot tV_{2}^{2} + 1.71966\cdot 10^{-2} \cdot tV_{2} - 2.85373\cdot 10^{-2}$.

## Pressure Sensor (PS)

### PS Science Objectives and Requirements

The MEDA Pressure Sensor (PS) will continue a long history of pressure measurements from the surface of Mars that began with the Viking landers and continue through the currently operating Mars Science Laboratory/Curiosity rover and the InSight lander (Harri et al. 2014a; Haberle et al. 2014; Banfield et al. 2020). Surface pressure measurements are important for understanding the global dynamics, mass balance, CO_2_ and dust cycles, etc. among other interesting phenomena of the Martian atmosphere, which in turn enables a better understanding of the current Martian climate and a higher probability of being able to successfully model a past Martian climatic state.

The current Martian atmosphere is 95.1% CO_2_ (Trainer et al. 2019), of which ∼30% condenses out in polar caps annually. Surface pressure sensors are well-suited to tracking this process, and by comparing MEDA measurements to those over the past 45 years, it may be possible to discern any differences in this annual global cycle (Haberle and Kahre 2010). Another large-scale feature of the Martian atmosphere that can be detected is the overturning Hadley-cell circulation (Haberle et al. 1982; Wilson 1997), the strength of which should vary seasonally at the M2020 Jezero Crater landing site at ∼19^∘^ N. Diurnal and semi-diurnal tides, and the effect of dust storms on them, can also be detected from a surface pressure sensor (e.g. Guzewich et al. 2016). Surface pressure measurements can also be used to detect regional atmospheric changes such as those caused by baroclinic instabilities (Barnes 1980; Haberle et al. 2017) and at low latitudes can be used to detect the equatorial reach of high latitude storm activity (e.g. Haberle et al. 2017). Surface pressure can also detect the signal of local topographically-driven changes such as hydrostatic adjustment (slope) flows, gravity waves, or low-elevation pooling of air (Haberle et al. 2014; Tyler and Barnes 2015; Rafkin et al. 2016; Richardson and Newman 2019). Finally, the low-pressure core of convective vortices (clear ones or dust-filled “dust devils”) can be detected when passing over the landed asset, as has been done from Mars Pathfinder to InSight (e.g. Murphy and Nelli 2002; Ellehøj et al. 2010; Kahanpää et al. 2016; Steakley and Murphy 2016; Ordoñez-Etxeberría et al. 2018; Spiga et al. 2018; Banfield et al. 2020).

MEDA will build on the results of previous landers discussed above. Its landing location in Jezero crater, while a smaller crater, offers the potential to validate topography-driven pressure effects proposed based on MSL/Curiosity results (Haberle et al. 2014; Rafkin et al. 2016). Its latitudinal location is similar to Viking Lander 1, and between Curiosity and InSight, to the south, and Viking Lander 2, to the north. This location offers the possibility to observe baroclinic waves that may not be detectable at the more southerly locations of the currently operating Curiosity and InSight landed spacecraft.

The PS works in conjunction with other instruments on M2020/Perseverance to elucidate the state and variability of the local environment. Pressure, as well as temperature, is needed to provide the near-surface air density (which varies hugely with time of day and season), which in turn is needed to determine the wind stress at the surface. This is crucial to understand the linkage between meteorology, aeolian changes, and dust injection, as discussed further in Sect. 6. Imaging of dust devils by Mastcam-Z and the engineering cameras is particularly synergistic with the PS measurements of distinctive vortex pressure drops. Together, it is possible to constrain what fraction or type of vortices carry significant dust, and document their sizes and speeds.

The pressure sensor investigations and requirements shown in Table 4 were developed to support the M2020 science goals described above and the MEPAG (2014) goals stated in the table, below. Testing throughout development demonstrated the pressure sensor performance shown in the same table.

**Table 4:** Pressure sensor requirements

MEPAG Goals (2014)	Investigation	Requirements	Performance
Goal II, Obj. A1, A4 Goal III, Obj. A3 Goal IV, Obj. B4, B7.	Detection of dust devil pressure signatures.	The PS resolution shall be better than 0.5 Pa.	NGM resolution in nominal mode (1 Hz measurement) ∼0.12 Pa. RSP2M resolution in nominal mode in order of 0.3-0.4 Pa.
		The sensor shall be able to detect pressure changes with a variation speed of at least 1 Pa/s (PS’ response time shall be equal or better than [1 sec]).	On sensor level <1 Pa/s. On rover level achieved by heritage in the design (similar to REMS onboard MSL).
Goal II, Obj. A1, A4 Goal III, Obj. A3 Goal IV, Obj. B1, B7.	Seasonal and diurnal pressure cycles	The PS shall have a range of [1-1200 Pa] [Req. L4-MEDA-05]. Note: the calibration shall be optimized for Mars range 400-1200 Pa and also for high vacuum.	The sensor has been calibrated in the range of [0–1400 Pa].
Goal II, Obj. A1.	Pressure oscillations on the diurnal and hour scale	The PS accuracy shall be equal or better than [+/-20 Pa] from [1 to 400 Pa] and [+/-10 Pa] from [400 to 1200 Pa], over the operational temperature range of [-45 C to +55 C] [Req. L4-MEDA-43].	With preliminary calibration coefficients, the accuracy of measurements performed during rover level tests is better than +/-5 Pa. Final calibration coefficients and accuracy BOL will be determined after the cruise calibration checkout.

### PS Design Description

MEDA PS is a miniature pressure device based on Finnish company Vaisala, Inc. Barocap^®^ sensor head and transducer electronics. The transducer measurements are controlled by Vaisala proprietary ASIC. The technology of the Barocap^®^ is well known and it has previously flown in 6 missions, including MSL (REMS-P) and ExoMars 2016/Schiaparelli lander (DREAMS-P). MEDA PS design is very similar to REMS-P (Harri et al. 2014a), inheriting some parts also from DREAMS-P (Esposito et al. 2018).

**Fig. 6:**
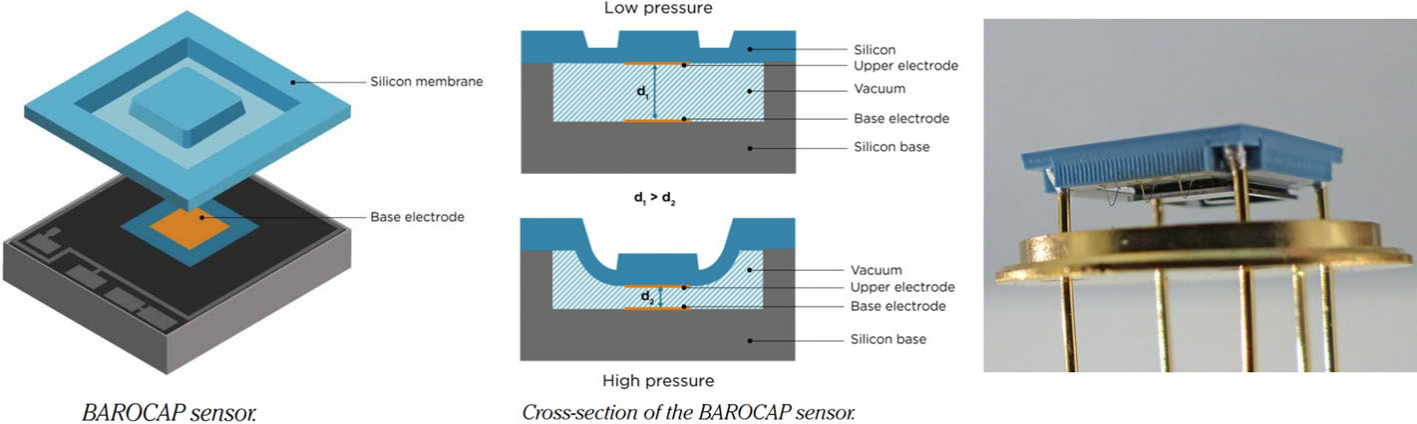
Schematic of the Barocap^®^ sensor head, drawing by Vaisala, Inc. (left). Barocap^®^ sensor head type NGM (right)

Barocap^®^ is a micro-machined capacitive pressure sensor head. Pressure moves the capacitor plates in the sensor, changing its capacitance, as shown in Fig. 6. This movement is not sensitive to the composition of the gas medium, resulting in the same capacitance change in terrestrial air or for the Mars CO_2_ atmosphere.

The capacitance of the sensor head is not only pressure, but also temperature dependent, and so accurate reference temperature measurement is needed to correctly interpret the output capacitance of the sensor head. The nominal capacitance of NGM and RSP2M type Barocap^®^ sensor heads used in MEDA PS is around 27 pF and 14 pF, respectively. These sensor head types have been specifically modified and manufactured by Vaisala, Inc. for FMI for Mars applications.

MEDA PS contains 2 pressure transducers (oscillators) on the same multilayer printed circuit board (PCB), each with its own controlling ASIC and 8 channels containing Barocap^®^ sensor heads, Thermocap temperature sensor heads, and constant reference capacitors. The temperature sensor heads are located directly on the PS PCB close to the pressure sensor heads to provide accurate housekeeping temperature measurements needed to support Barocap^®^ measurements. The output of each of the 8 channels in a transducer is frequency [Hz], and the reference channels with constant capacitors are used for calculating the capacitances of the sensor heads from the frequency output. The process of obtaining capacitance values from frequency is Vaisala proprietary and is not described in this paper.

There are altogether 3 pressure channels and 2 housekeeping temperature channels in PS transducer 1 (called P1), and 2 pressure and 2 housekeeping temperature channels in transducer 2 (P2), listed in Table 5. P1 is the primary transducer for science.

**Table 5:** PS Barocap^®^ and Thermocap channels

Transducer	Channel #	Sensor head type	Note
P1	1.1	Barocap^®^ NGM	
P1	1.4	Thermocap	
P1	1.5	Thermocap	
P1	1.6	Barocap^®^ RSP2M	Secondary Barocap^®^ for science
P1	1.8	Barocap^®^ NGM	Primary Barocap^®^ for science
P2	2.1	Barocap^®^ RSP2M	
P2	2.4	Thermocap	
P2	2.5	Thermocap	
P2	2.6	Barocap^®^ RSP2M	

Table 6 shows a summary of the most relevant physical and electrical characteristics of MEDA PS.

**Table 6:** PS properties

PS	Value
Dimensions	62×50×17 mm (without pipe)
Mass	40 g
Power consumption	15 mW

NGM Barocap^®^ is a new sensor head type for Martian applications by Vaisala with the best resolution so far and very good stability. Because of its size and structure it has bigger temperature dependence than RSP2M, which makes it more susceptible to temperature changes and can result in longer warm-up time after power on. The heating effect can, however, be compensated by data processing. The RSP2M, the sensor head used also in REMS-P sensor onboard MSL, has slightly worse resolution than NGM, and worse stability, but it’s warm-up time is less than 2 s. Unlike NGM, which is shorted in pressures above ∼50–75 hPa, RSP2M can also be measured in ambient Earth conditions.

**Fig. 7:**
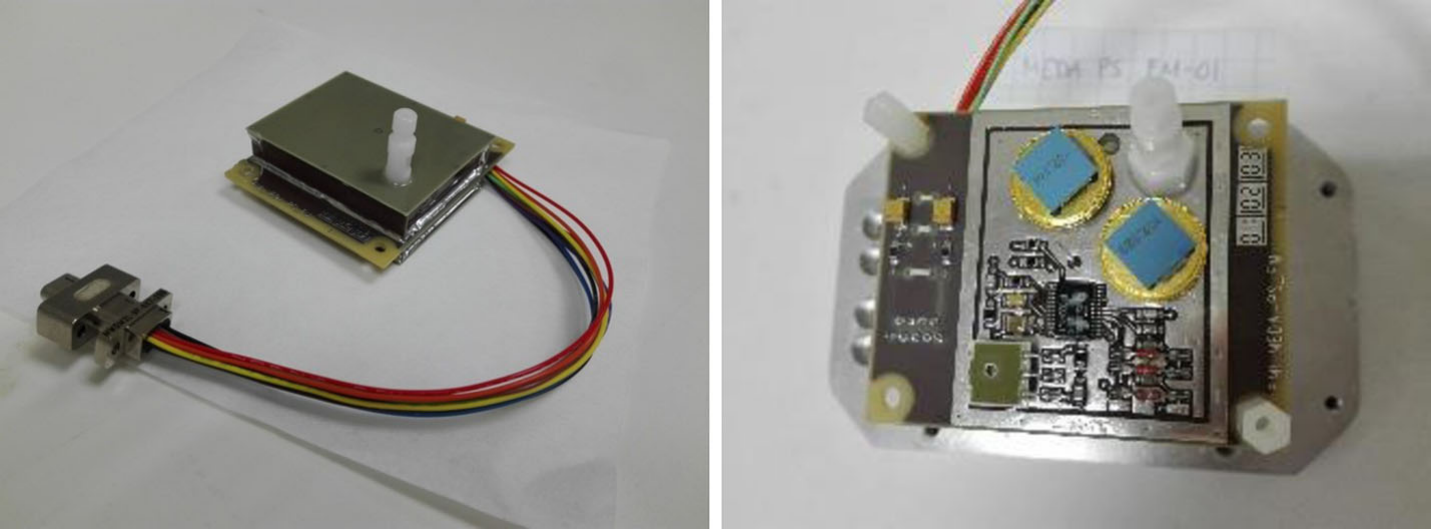
Pressure sensor with the pipe protruding through the Faraday shield (left). Right: PS PCB top containing P1 electronics without a cover. NGM Barocaps^®^ are in top right corner, RSP2M 1.6 in bottom left. Thermocap sensor heads are reddish glass tubes in bottom right corner

PS electronics are protected by box-like Faraday shields made of PCB material. Fig. 7 shows a picture of the actual MEDA PS. PS is located in MEDA Instrument Control Unit (ICU) box inside rover body in a temperature-controlled compartment, and connected to the atmosphere through a dedicated pipe. The pipe exits the rover body through a small opening in Rover Avionics Mounting Panel.

### PS Calibration

#### Overview

**Fig. 8:**
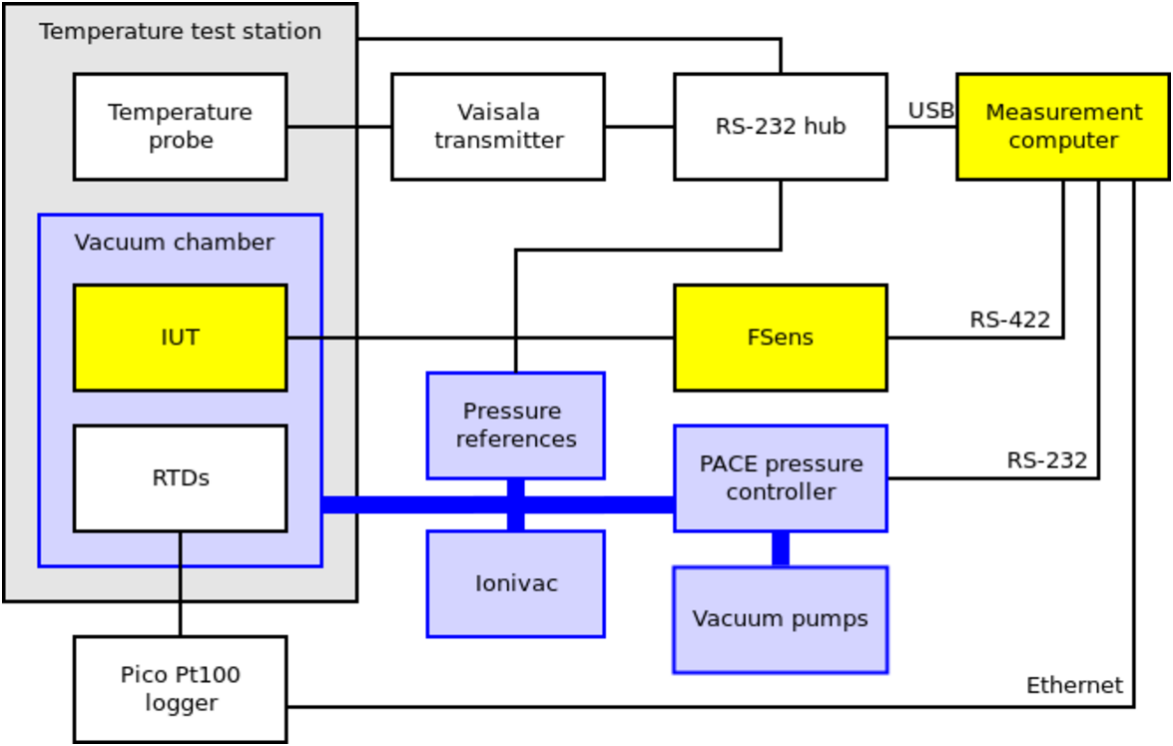
Block diagram of calibration equipment at FMI

**Fig. 9:**
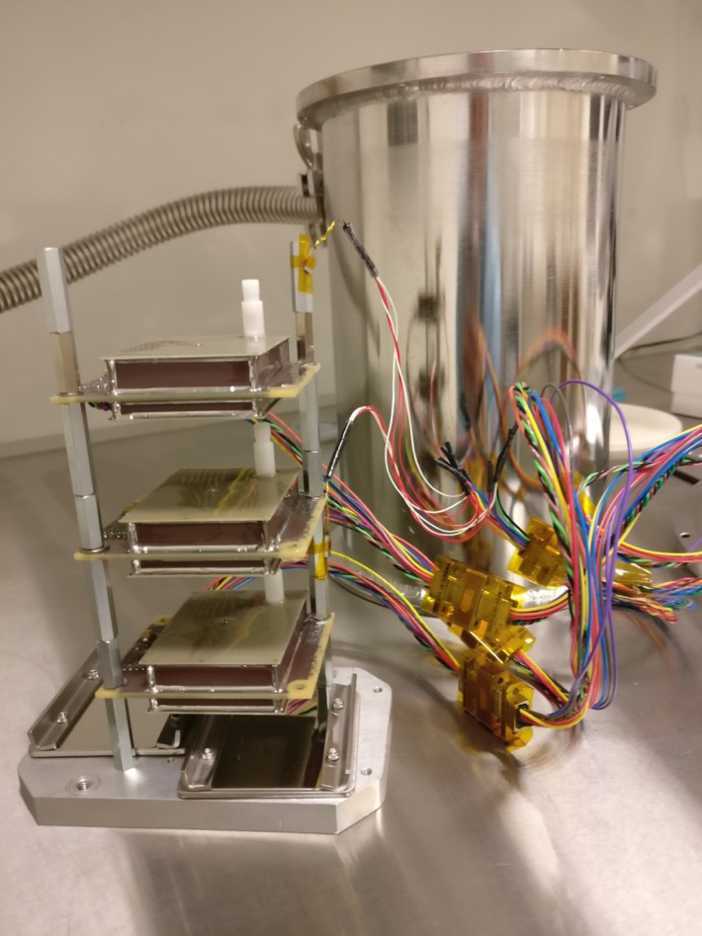
MEDA PS Flight, Spare and Qualification models attached to a support plate. Pressure vessel used in the MEDA PS tests is visible behind the models

The first stage of the pressure and housekeeping temperature calibration of the PS was performed in FMI calibration laboratory using the setup shown in Fig. 8 and Fig. 9. The pressure calibration was done against transfer references, which in turn were calibrated against a national standard reference sensor. The total accuracy of the reference pressure sensor (Baratron 10 Torr) was 1 Pa, and the time constant was 400 ms in the nominal position and 25 ms in the fast position (MKS Instruments 1997). The reference temperature accuracy was 0.25 ^∘^C.

The test equipment consists of measurement electronics for measuring and controlling the instruments’ oscillators and of environmental control and reference equipment. The same measurement computer controls everything: the instrument measurements, reference transmitter measurements and the environment. The tests are run with “MSens” software written by FMI.

Thermal vacuum environment is created by placing the pressure vessel inside the temperature test station. Temperature inside the vessel can then be controlled within the operational range of the temperature test station. Temperature inside the pressure vessel is measured with two dedicated Pt100 temperature sensors. High vacuum is achieved with a combination of a rotary vane vacuum pump and a turbomolecular pump. In the Martian pressure range the pressure inside the pressure vessel is controlled with a commercial PACE pressure control unit.

Because water ingassed into the Barocap^®^ sensor heads and PCB material both affect the capacitance of the sensor heads, the PS was allowed to outgas in warm (50–55 ^∘^C) vacuum for a minimum of 48 hours before each calibration run. Thus, the PS outgassing conditions were comparable to the conditions after cruise to Mars.

**Fig. 10:**
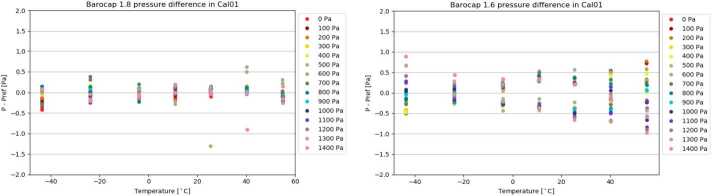
Short-term repeatability of Barocaps^®^ 1.8 (left) and 1.6 (right) in the main calibration run

PS was calibrated in both stable and changing environmental conditions. The main calibration run (so called Full Calibration) was performed in 7 stable temperature points ($\Delta $T < 0.5 ^∘^C/h) in the range of [-45 ^∘^C to +55 ^∘^C], and in the pressure range of [0 Pa to 1400 Pa] with 100 Pa steps. In each temperature/pressure point, all channels of both oscillators were measured for several minutes. Fig. 10 shows the results obtained in this main calibration run for Barocap type NGM (channel 1.8), and Barocap type RSP2M (channel 1.6) of Transducer 1.

In addition to stable conditions, measurements were made in slowly changing temperature but stable pressure to measure the impact of changing temperature on the readings of the sensor heads. The time response of the sensor was also measured in one stable temperature point during a sudden pressure drop of approximately 10 Pa, which is comparable to the changes caused by vortices (e.g. Kahanpää et al. 2016).

Shorter calibration runs (so called Check-outs) with fewer temperature and pressure points than in Full Calibration were performed between PS environmental tests to measure the impact of environmental stresses and long-term stability of the pressure sensor heads.

A calibration check with random temperature/pressure points chosen from the operational range and using ground support equipment with the MEDA FPGA code for controlling the PS and reading its raw output was performed to validate the FPGA code used in flight.

In addition to ageing drift, the Barocap^®^ sensor heads are known to have changes in their capacitance when integrated to a new electrical and thermal environment after calibration. The changes can be both pressure and temperature dependent. Because of this, the preliminary calibration done at sensor level is complemented with higher-level calibration checks at ICU and Rover level. The complementary calibration shall be done at least in high vacuum in several temperature points, but for the best results the measurements should also be done in the Martian pressure range. The final fine-tuning of the calibration is done using measurements performed during the cruise to Mars.

Complementary calibration of Barocaps^®^ has been performed first on the ICU level in high vacuum after ICU Thermal Vacuum Test, in several temperature points from the operational temperature range. In the Rover-level System Thermal Tests (STT), further calibration checks were performed both in vacuum and in 8 Torr atmosphere. The measurements from these tests, together with the measurements to be done in the cruise phase, will be used for correcting the calibration parameters obtained from the sensor-level calibration.

#### Calibration Equations

Readings of the Barocap^®^ sensors are temperature dependent, and thus the temperature of the sensor head is needed for calculating the Barocap^®^ pressure. The temperature $T_{T}$ [ ^∘^C] measured by the Thermocap^®^ sensors is solved from

1 $$ O_{T} + G_{T} T_{T} = \frac{1}{A_{T}-C_{T}} $$

where $C_{T}$ is the sensor capacitance [pF] and $O_{T}$, $G_{T}$ and $A_{T}$ calibration parameters.

The instrument heating affects the thermal profile of Barocaps^®^ in a different way than for Thermocaps. The effective Barocap^®^ temperatures [ ^∘^C] for each time point $t_{i}$ are calculated recursively by

2 $$ T_{\mathit{eff},i} = T_{\mathit{eff},i-1} + (1-e^{\kappa (t_{i-1}-t_{i})})(T_{T,i}-T_{\mathit{eff},i-1}) $$

where $T_{T}$ is the temperature measured by one of the Thermocap sensors and

3 $$ \kappa = e^{-k} $$

where $k$ is a constant factor. The initial condition is calculated using an offset value $\Delta T$ (= 0 in the preliminary calibration) by

4 $$ T_{\mathit{eff},0}=T_{T,0}-\Delta T $$

The Barocap^®^ behavior is initially treated by defining an inverse quantity

5 $$ V = \frac{1}{C_{\infty }-C_{\mathit{eff}}} $$

where the effective capacitance [pF] is calculated from an offset value $\Delta C$ (= 0 in the preliminary calibration) by

6 $$ C_{\mathit{eff}} = C-\Delta C $$

The “capacitance at infinity” [pF] is defined as

7 $$ C_{\infty }= \sum _{n=0}^{N-1} c_{n} T_{\mathit{eff}}^{n} $$

The series expansion is determined by dividing the calibration data into bins according to the measurement temperature and finding $C_{\infty }$ at these temperatures. Optimal rank is determined by k-folding and the model then created by polynomial regression. After calculating the $V$ values, the model for the pressure [Pa] becomes

8 $$ p = \sum _{m=0}^{M-1} \sum _{n=0}^{N-1} d_{mn} V^{m} T_{\mathit{eff}}^{n} $$

The optimal rank for each argument is again determined by k-folding and the coefficients found by polynomial regression.

The calibration was complemented with ICU level measurements in high vacuum and 5 temperature points. The Barocap^®^ pressure readings are corrected with a temperature-dependent offset $p_{\mathit{corr}}$ determined for each sensor head.

9 $$ p_{\mathit{corr}} = a_{\mathit{icu}}T_{T}^{3} + b_{\mathit{icu}}T_{T}^{2} + c_{\mathit{icu}}T_{T} + d_{\mathit{icu}} + p_{0} $$

The parameter $p_{0}$ is not temperature dependent and it compensates for the drift and offset-type changes of the Barocaps^®^ after integrating them to a new environment (for example the rover). After the ICU level calibration $p_{0}$ was set to 0. The final value for $p_{0}$ will be obtained from measurements performed during the cruise phase close to the landing. Once the final correction is obtained, equation (8) becomes

10 $$ p = \sum _{m=0}^{M-1} \sum _{n=0}^{N-1} d_{mn} V^{m} T_{\mathit{eff}}^{n} + p_{\mathit{corr}} $$

#### Results

The results presented in this section are preliminary based on the sensor-level calibration and the ICU-level calibration check. Final calibration results, including the estimated drift rate for the Barocap^®^ sensor heads, can be presented after the cruise phase.

##### Short-Term Repeatability

The short-term repeatability is calculated as the difference between the calibrated Barocap^®^ pressure and the reference pressure during the main calibration run at sensor level. It represents the repeatability of the sensor in the timescale of minutes. The difference is determined at each temperature and pressure point.

##### Resolution

The Barocap^®^ resolution is defined as the width of the 90% confidence range of the statistical error of the pressure value. Assuming that the statistical error has a Gaussian distribution, this means

$$ \mathit{res} = 2*1.96*\sigma $$

where $\sigma $ is the standard deviation of the pressure value. As the real pressure slowly changes during the measurements, a polynomial of degree 2 is fitted to the Barocap^®^ pressure against time and the standard deviation calculated from the difference between the measurement and the fit. Table 7 presents the resolutions of Barocaps^®^s 1.8 and 1.6 calculated from the ICU level measurements in three different modes.

**Table 7:** Barocap^®^ 1.8 and 1.6 resolutions in ICU level measurements

Barocap^®^	Nominal mode	High resolution mode	High resolution 0.5 Hz mode
**1.8**	0.13 Pa	0.10 Pa	0.08 Pa
**1.6**	0.32 Pa	0.23 Pa	0.19 Pa

##### Time Constant

**Fig. 11:**
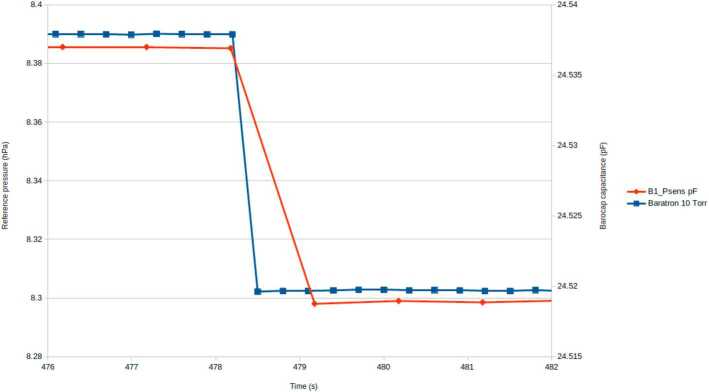
Time constant of the PS QM

The time constant was measured at sensor level in the Martian pressure of about 800 Pa. The PS model used in the test was the Qualification Model (QM). The time constant was demonstrated by making sudden pressure changes in steps. The reference sensor was set to measure every 0.25 s, and the PS was in the nominal mode measuring once per second. Figure 11 shows that both the reference and the PS sensor reacted to the sudden pressure drop in less than 1 s. The time constant at rover level is verified by similarity to the MSL rover.

##### System Thermal Test (STT) Results

**Fig. 12:**
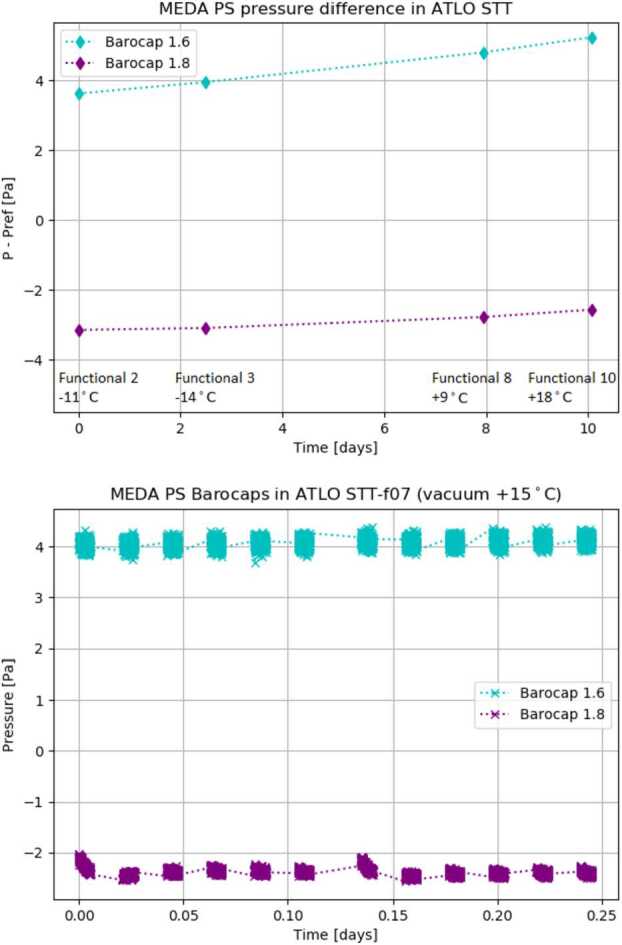
(Left) Difference between the sensor pressure and the reference pressure of Barocaps^®^ 1.6 and 1.8 in Martian pressure STT (no offset correction, $p_{0} = 0$). A drift of around 1 Pa between the first and the last test can be contributed to the outgassing of the instrument. (Right) Barocaps^®^ 1.6 and 1.8 readings in STT vacuum measurements. These measurements can be used for determining interim $p_{0}$ offset parameter of the Barocaps^®^

**Fig. 13:**
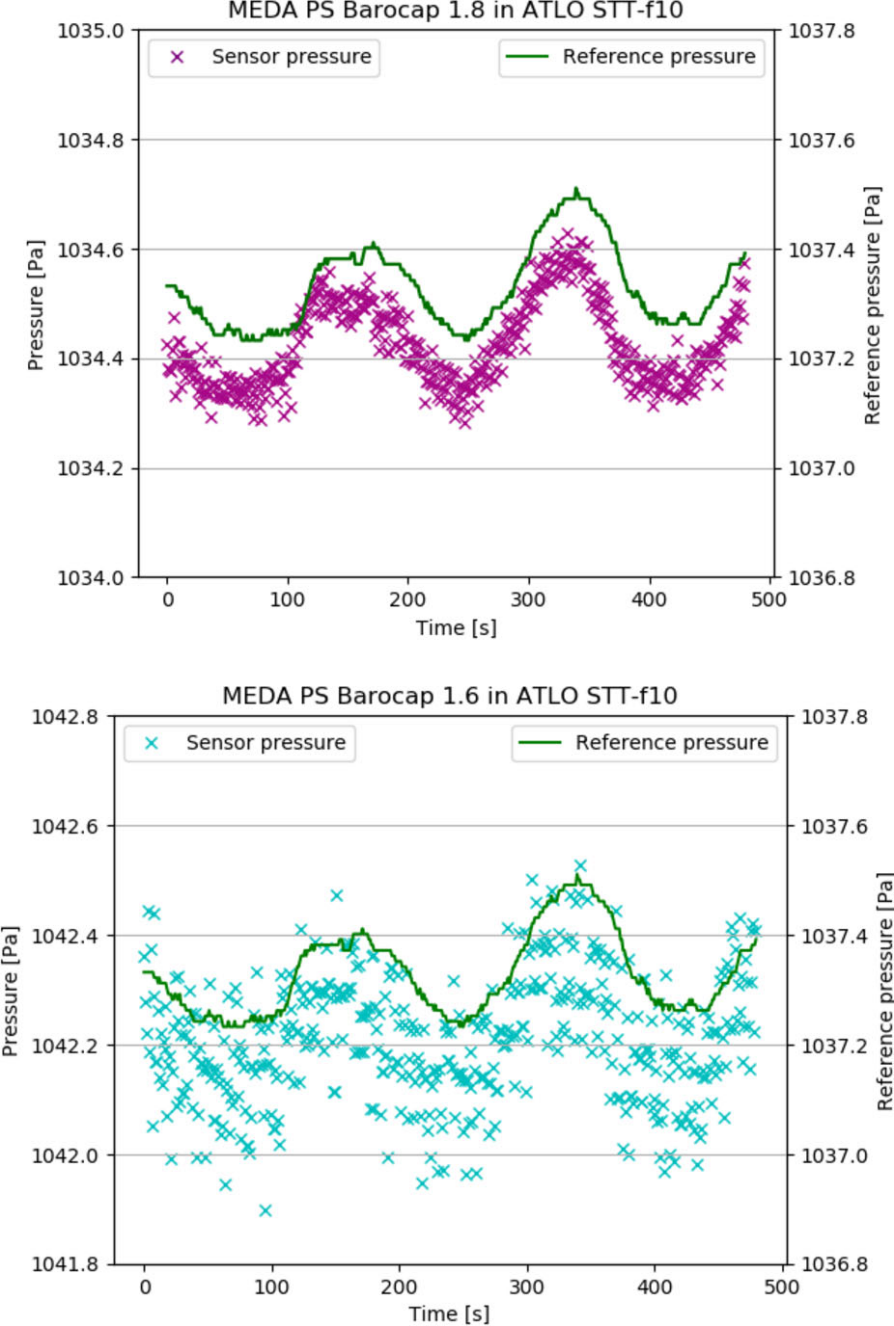
Barocaps^®^ 1.8 (left) and 1.6 (right) with the reference pressure during a single measurement in STT functional test (no offset correction, $p_{0} = 0$)

Figure 12 shows how the pressure difference of the primary Barocap^®^ 1.8 and the secondary Barocap^®^ 1.6 varied in measurements performed during STT in Martian pressure (∼1000 Pa) (left), and in vacuum (right). The pressure values of Barocaps^®^ are calculated using the calibration obtained in sensor-level calibration run, and corrected with the temperature-dependent correction determined during ICU level tests ($p_{0}$ set to 0). A single 8-minute measurement during the functional test 10 is shown closer in Fig. 13 to demonstrate the noise level and the warm-up effect of the Barocaps^®^. The effect has been minimized by the calibration.

If updated with the offset correction parameter $p_{0}$ set to the interim value obtained during STT vacuum measurement, the difference between the main and secondary Barocap^®^ reading and the reference sensor reading in the end of the STT would be around 1 Pa for both the primary Barocap^®^ 1.8 and the secondary Barocap^®^ 1.6. The other Barocaps^®^ (plots not shown here) behave similarly, but with somewhat less accurate results.

#### Planned in-Flight Check-Outs/Calibrations

During the cruise, PS will be measuring 2 times to obtain a corrected value for $p_{0}$, with the last measurement done as close to the landing as possible. A comparison of these 2 measurements and the STT measurement done in vacuum will also be used for estimating the drift rate of the Barocaps^®^.

### PS Operations Concept

#### Commanding

PS is powered on from MEDA ICU. Only one transducer may be powered on and operated in turn. After powering on, the ASIC of the powered transducer is controlled by the MEDA FPGA. The FPGA has 2 registers for controlling the PS, and 26 registers for storing raw output data of the PS. The control registers contain information about the PS operational mode. The FPGA also provides the PS ASIC multiplexer reset and step signals that allow stepping through the oscillator channels and measuring them in turn. For each measurement, the raw frequency output of each channel is stored in the FPGA before being read by the ICU processor.

#### Modes

PS can be operated with a 1 Hz or 0.5 Hz measurement frequency. Different numbers of reference clock pulses can be used for integrating the channel frequencies of the oscillator. Lower measurement frequency allows using more reference pulses, meaning more time for integrating the oscillator channel frequencies. This results in higher resolution of the channel outputs, but on the other hand the time resolution of the sensor is then lower.

In the nominal mode, the raw frequency of each channel of the oscillator is measured with an equal number of reference clock pulses, with 1 s or 2 s total measurement time depending on the chosen measurement frequency. In the high-resolution mode, all reference channels are not measured each 1 or 2 second(s) but in turn, allowing more integration time for the main Barocap^®^ channel, and thus resulting in somewhat better pressure resolution without losing time resolution.

#### Expected Usage

The primary transducer for scientific operations is P1. P2 serves as a backup transducer but shall be measured occasionally also to compensate for the drift of RSP2M Barocaps^®^. The calibration results show that in practice the pressure resolution of the primary NGM Barocap^®^ (channel 1.8) in P1 is not significantly affected by the measurement mode. Thus, the nominal mode with 1 Hz measurement frequency and 550 reference clock pulses can be used as the primary science mode.

### PS Data Format

The raw output of the pressure sensor is the number of clock pulses corresponding to the oscillating frequency of each channel. Depending on the measurement mode (see Sect. 3.4.2), a data packet containing the raw values of each measured oscillator channel is read by the ICU every 1 or 2 seconds and delivered to ground as part of MEDA telemetry packets. All data processing of the raw values is done on ground.

## Humidity Sensor (HS)

### HS Science Objectives and Requirements

Humidity sensors are used to understand the Martian hydrological cycle. The water fluxes between the surface and lower atmosphere in the vicinity of the rover can be discerned, boundary conditions for circulation models can be inferred by measuring near-surface water vapor amounts, and orbital retrievals of water abundance can be constrained and improved (see e.g. Rodriguez-Manfredi et al. 2014; Savijärvi et al. 2020; Tamppari and Lemmon 2020).

The MEDA relative humidity sensor (HS) will represent the third time that relative humidity has been measured from the surface of Mars. Previous spacecraft that flew relative humidity sensors were the Phoenix lander done (Zent et al. 2009, 2010, 2016; Fischer et al. 2019) and the Mars Science Laboratory/Curiosity rover (Gómez-Elvira et al. 2014; Harri et al. 2014b; Martínez et al. 2017). Near-surface relative humidity measurements on diurnal and seasonal time scales are important for understanding the distribution of water in the Martian atmosphere, the likelihood of stable, near-surface ice or brines, and the general modern habitability potential of the local environment.

The HS will enhance the data returned by other ground-based Mars missions by adding additional time coverage and an additional location with a potentially different environment to the previous measurements. Further, improvements in the HS from the Curiosity rover version have been made to enable a more rapid response to the environment and to have a wider dynamic range, allowing for better resolution in the measurements. In addition, the location between the equatorial Curiosity and the polar Phoenix missions, along with the simultaneous operations of relative humidity measurements at Curiosity and Perserverance, will enable us to deepen our understanding of the variability in near-surface environments across Mars and the global water cycle.

In combination with independent measurements by the SuperCam or TIRS instruments, measurements of relative humidity can be used to detect frost formation (Martinez et al. 2016), to check whether the environmental conditions at the surface and in the near surface are compatible with the formation of liquid brines (Martin-Torres et al. 2015; Fischer et al. 2016; Rivera-Valentin et al. 2018), and to assess diurnal changes in the near-surface water content, possibly due to subsurface exchange (e.g. Martínez et al. 2017; Savijärvi et al. 2019; Tamppari and Lemmon 2020), local meteorology events (Ordoñez-Etxeberría et al. 2018; Viudez-Moreiras et al. 2019c) or consistent with surface frost (Martinez et al. 2016). Site-specific characteristics can also be discerned, such as the unusually dry air in Gale crater (Harri et al. 2014a).

The relative humidity sensor will support the goals of the mission discussed in the introduction and will also support sample collection by documenting the atmospheric field context in which the sample was acquired. It will be particularly important to understand the relatively humidity of the atmosphere that may be trapped with the sample and stored on the surface of Mars for 5+ years, as water vapor sets the initial conditions that may influence the sample preservation chemistry over time.

To satisfy the goals and objectives discussed above, the relative humidity sensor requirements shown in Table 8 were developed. Testing throughout development demonstrated the sensor performance shown in the same table.

**Table 8:** Relative Humidity sensor requirements

MEPAG Goals (2014)	Investigation	Requirements	Performance
Goal I, Obj. B1.1	Annual and diurnal cycles of relative humidity.	The HS shall have a range of [0 to 100%] over a Mars temperature range of [190 K to 270 K].	The sensor has been calibrated in low pressure CO_2_ atmosphere, in dry (0% RH) conditions in temperature range of [203 K to 295 K], and saturation (100% RH) conditions in temperature range of [203 K to 233 K]. The results (dry and saturation curves) can be extrapolated to the temperature range of [190 K to 270 K].
		The HS resolution shall be equal or better than [0.5%] in the time scale of seconds over the range of [190-270 K].	Resolution depends on temperature. Resolution, limited by electronics noise level, is 0.1% RH at 203 K.
Goal II, Obj. A1, A4	Sudden changes in RH.	The HS’ response time shall be equal or better than [30 min] for temperatures above 203 K.	Humicap^®^ response time without cover is in order of 450 s in 203 K temperature. In warmer temperatures the lag is shorter, for example in order of 30 s in 233 K.
Goal IV, Obj. G	Nocturnal hydrological cycle.	The HS’ accuracy shall be equal or better than [+/-10% RH] for atmospheric temperatures above 203 K, and equal or better than [+/-20% RH] for temperatures in the range [190-200 K].	+/-10% RH down to 203 K, better than +/-20% RH down to 190 K.

### HS Design Description

**Fig. 14:**
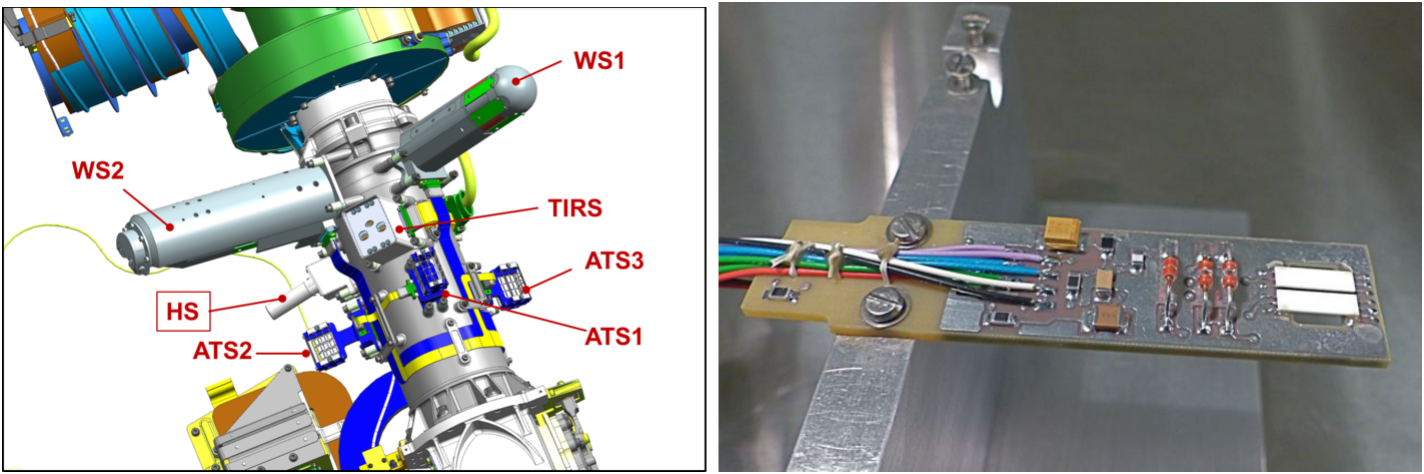
(Left) HS location on the Remote Sensing Mast. (Right) Detail of HS PCB without covers. Humicaps^®^ on right (white rectangles), Thermocap sensor heads next to them

The humidity sensor electronics are based on the same transducer design as the pressure sensor (see Sect. 3.3), but instead of pressure sensor heads it has humidity sensor heads Humicap^®^ by Vaisala, Inc. The HS has one transducer with 8 frequency channels containing sensor heads and constant reference capacitors needed to interpret sensor head capacitance. In addition to the frequency channels, the resistance of the PT1000 temperature sensors inside the Humicap^®^s is read directly by the MEDA ICU. All HS proximity electronics and sensor heads are placed on a single multilayer PCB protected by a round metallic perforated Faraday cage covered with a PTFE dust filter. The PCB with its components except the cover or other mechanical interface parts is presented in Fig. 14.

#### Measurement Principles

Humicap^®^ contains an active polymer that changes the sensor head capacitance as a function of relative humidity and temperature with 0 to 100% RH measurement range. At a given temperature, the response between 0 and 100%RH is close to linear. The polymer reacts to the relative humidity even when the device is not powered, so the relative humidity can be read almost immediately after power-on. The Humicap^®^ RSH045 type used in the MEDA humidity sensor has the same polymer and working principle as the older RS92 Humicap^®^ used in REMS onboard MSL, but it has several advantages: higher capacitance (45-50 pF in room temperature), 9x larger dynamic range (in order of 9 pF at room temperature, 2.5-3 pF at -70 ^∘^C) and in-built PT1000 platinum resistance temperature sensors providing accurate temperature of the sensor chip itself.

The dynamic range of the Humicap^®^ changes with temperature, and the sensor also becomes logarithmically slower with decreasing temperature, its time constant is about 0.1 s at 293 K, but for example at -40 ^∘^C it is about 30 s and at -70 ^∘^C about 450 s. Because the sensor is always exposed to the martian environment and the temperatures are likely to range between ∼-90 ^∘^C and ∼10 ^∘^C, the HS readings at -70 ^∘^C will be interpreted as an integral or average over the last ∼450 s. The capacitance of Humicap^®^ is also affected by CO_2_, which makes it necessary to perform its calibration in a low-pressure CO_2_ environment.

#### Accommodation

The humidity sensor is accommodated close to the MEDA temperature sensors (ATS) on the rover Remote Sensing Mast, at 1.5 m height from the ground (Fig. 14, left). It is vented to the Martian atmosphere through a 0.2 micron PTFE filter which provides protection against dust.

Table 9 shows a summary of the most relevant physical and electrical characteristics of MEDA HS.

**Table 9:** HS physical dimensions

HS	Value
Dimensions	55 × 25 × 95 mm
Mass	45 g
Power consumption	21 mW

### HS Calibration

#### Overview

The humidity sensor flight model has been calibrated together with the spare model and ground reference model at the Finnish Meteorological Institute. All three instruments went through multiple different calibration tests in ambient pressure, Martian pressure and vacuum. Both air and CO_2_ gas have been used in the calibration tests when applicable. In addition to the calibration tests performed at FMI, a test campaign with the ground reference model was carried out at the University of Michigan to verify the calibration results and one more campaign with the ground reference model is foreseen in the Mars Simulation Facility (MSF) and Planetary Analog Simulation Laboratory (PASLAB) at the German Aerospace Center (DLR).

#### Calibrations Performed at the Finnish Meteorological Institute

**Fig. 15:**
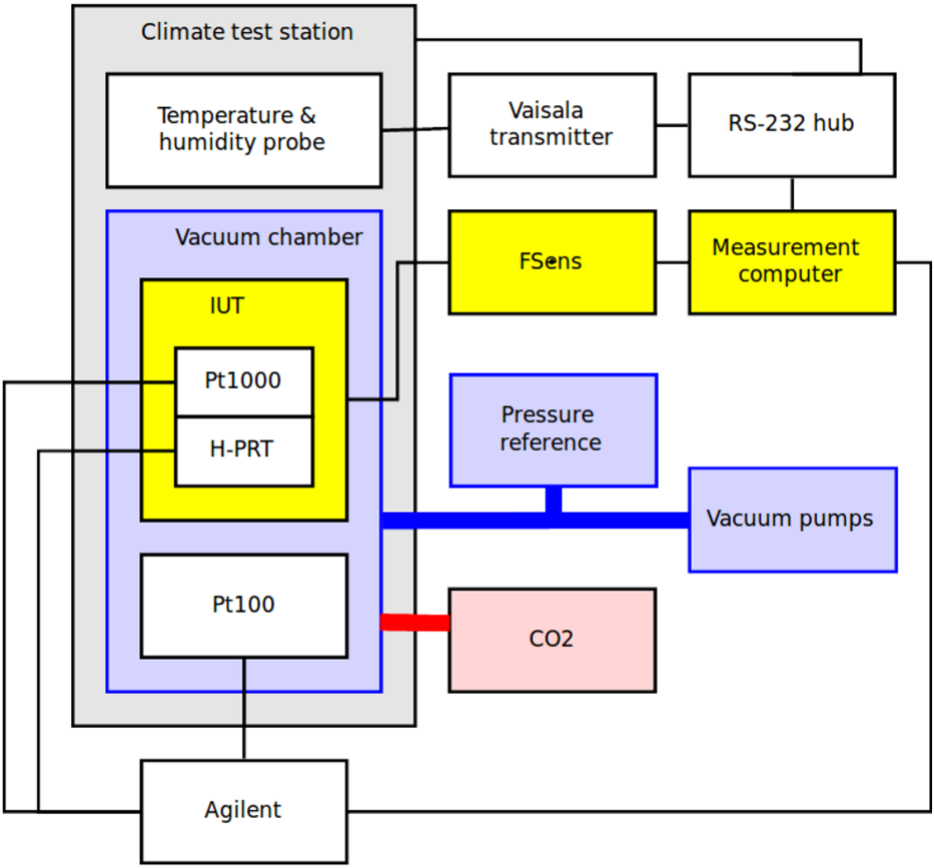
Block diagram of the calibration equipment

FMI has developed a dedicated test laboratory for humidity sensor calibration purposes. The block diagram of the laboratory is presented in Fig. 15. Instruments under test (IUT) are placed inside a climate test station in a pressure vessel. The pressure vessel provides a stable temperature environment for the instruments and can be connected to a vacuum pumping system, pressure control system and CO_2_ bottle as applicable in each test.

The calibration campaign at FMI consisted of:

1. Temperature calibration.
2. Humidity characterization in ambient pressure air in +22 ^∘^C.
3. Saturation point calibration in ambient pressure air.
4. Dry point calibration in vacuum and in low pressure CO_2_.
5. Saturation point calibration in low pressure CO_2_.

Dry and saturation point tests performed in ambient air and vacuum are not used in calculation of the final operational calibration.

**Fig. 16:**
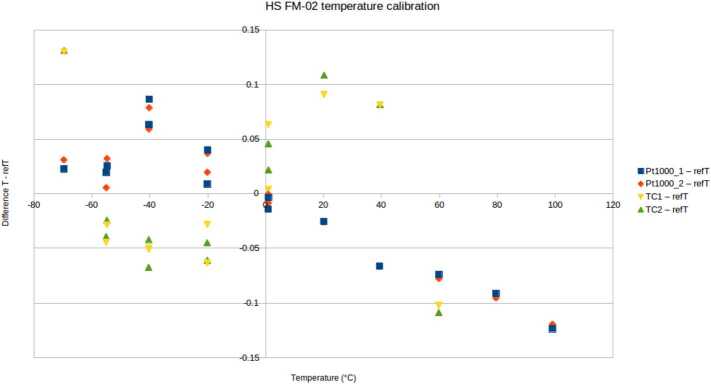
Thermocaps and PT1000 sensors difference to reference after temperature calibration

Temperature calibration was performed for Thermocap capacitive sensors, H-PRT heating resistors and PT1000 sensors in stable conditions against Netsushin NR-251 temperature sensors between +100 ^∘^C and -70 ^∘^C. The results are presented in Fig. 16.

Humidity characterization and saturation point test in ambient pressure air were performed for general characterization of the sensors and to check the condition of the instrument between environmental tests in room conditions. Humidity characterization was performed in a climate test chamber (in air, ambient pressure) in +22 ^∘^C stable temperature. The HS models were set inside the pressure vessel but the vessel was vented through a filter to the climate test station air flow. Different relative humidity points were set with the climate tests station and measured also with a Vaisala reference humidity transmitter HMT337.

Saturation points were achieved by enclosing the test specimen in a vessel with substantial humidity and cooling down the vessel until dew point/frost point is achieved. The highest measured temperature was -25 ^∘^C and the temperature was lowered to reach saturation or close to saturation points in -40 ^∘^C, -55 ^∘^C and -70 ^∘^C.

Dry point measurements give a 0% RH reference for humidity calibration. Dry point measurements are performed in high vacuum for checking purposes and in three different pressures in Martian range: 5.5, 7.0 and 8.5 hPa. Data from Humicap^®^ channels in low pressure CO_2_is used as dry point data in calibration coefficient calculation. In dry point tests the instruments were closed in a pressure vessel and placed inside a climate test station. The pressure vessel was connected either to high vacuum pumping system or Martian range pressure control system with CO_2_ gas supply. Seven temperature points between +22 ^∘^C and -70 ^∘^C were measured in all tests.

**Fig. 17:**
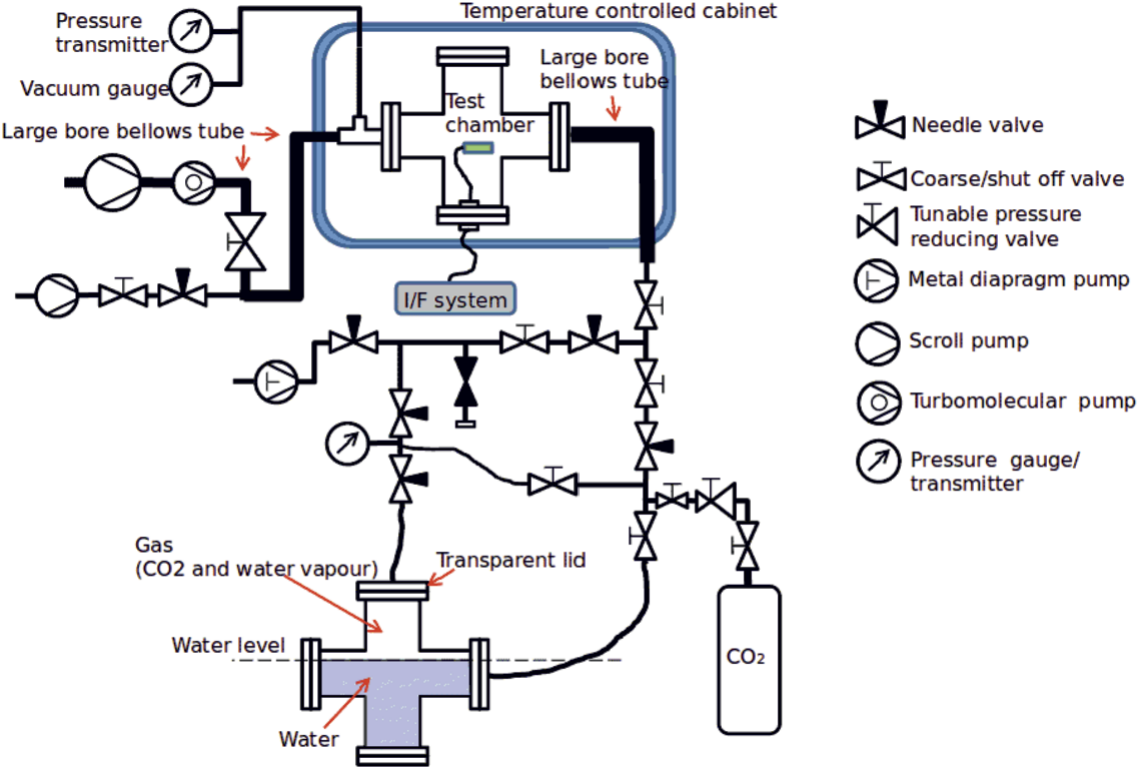
Humidity testing setup for Martian range tests

Saturation point calibration in low pressure CO_2_ requires a dedicated test equipment to be able to maintain low temperature, low pressure, CO_2_ environment and to be able to add sterile water inside the measurement volume to increase the relative humidity. The equipment used to achieve the saturation curve in a low pressure CO_2_ environment is presented in Fig. 17. The instruments were enclosed in a pressure vessel placed inside a temperature test station. The vessel was first vacuumed with pumping system and then a CO_2_ inlet was opened. CO_2_ gas was first routed through sterile water before entering the pressure vessel. The humid CO_2_ gas flow was manually controlled so that the pressure goal was reached and the gas was kept flowing until a saturation was reached. Temperatures were set and measured from -70 ^∘^C to -40 ^∘^C and back. The measurements were repeated in three different pressures: 5.5, 7.0 and 8.5 hPa. The temperature inside the pressure vessel was monitored with a Netsushin Pt100 sensor placed near the test specimen. As a pressure reference, a Vaisala PTB 201 Special, optimized for Martian pressure range was connected to the vessel. Humidity inside the pressure vessel was not recorded with a reference sensor.

#### Calibrations Performed at the University of Michigan

**Fig. 18:**
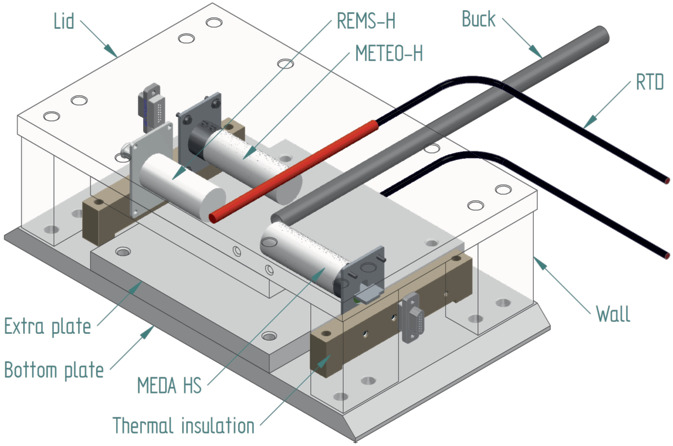
HS test setup in Michigan Mars Environmental Chamber

The HS ground reference model was tested in the Michigan Mars Environmental Chamber at the University of Michigan to verify the humidity calibration against a reference (Buck CR-1A chilled-mirror hygrometer). The measurement setup is presented in Fig. 18 where the reference hygrometer measurement tube is visible in the middle of the humidity instruments. The measurements in low pressure CO_2_ confirmed the dynamic range of the Humicap^®^ sensors but because of substantial uncertainties in the setup, including large thermal variations, the calibration curve between the 0% and 100% RH was not obtained with sufficient confidence.

#### Results

**Fig. 19:**
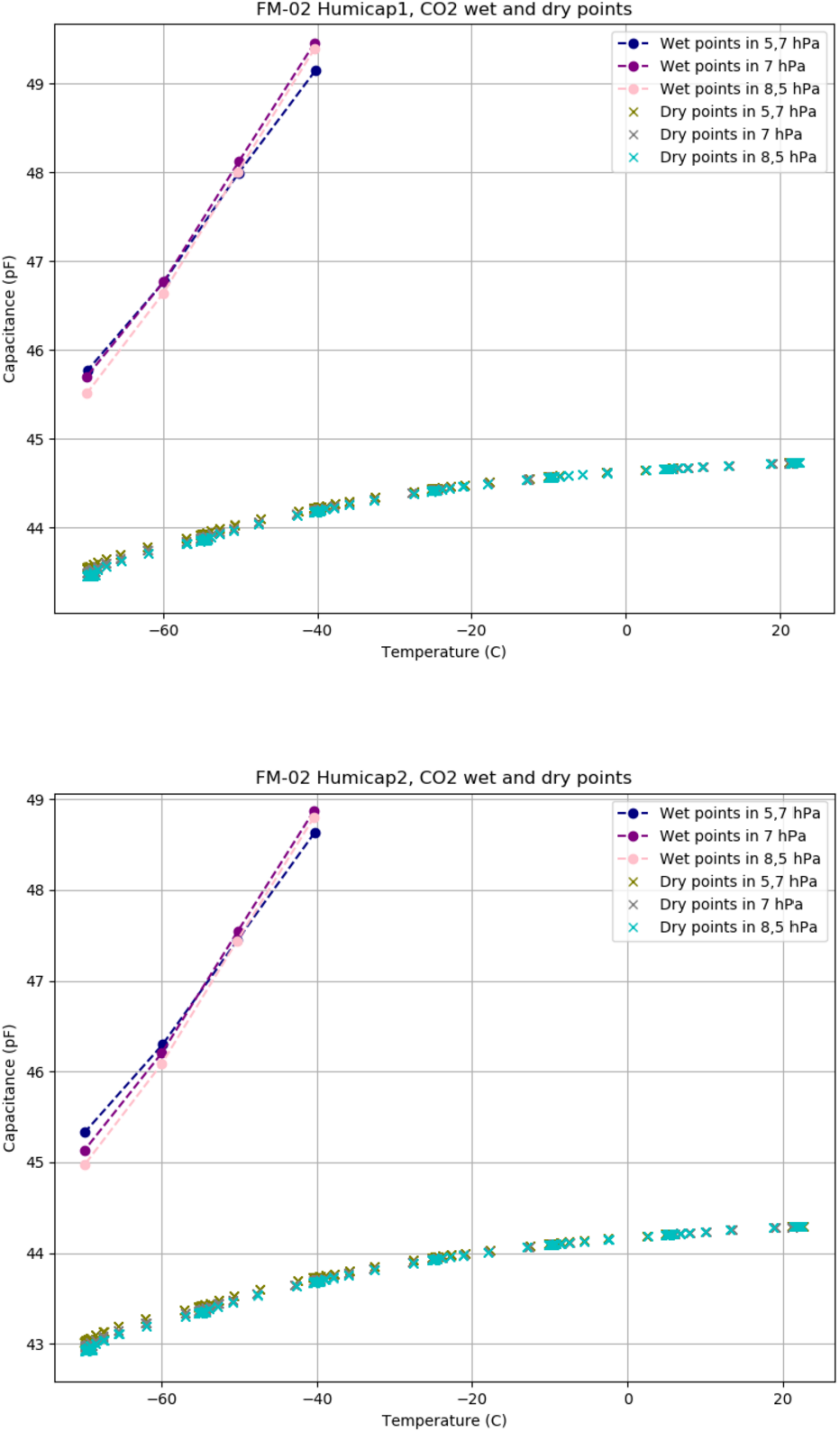
(Top) Saturation and dry points for Humicap^®^ 1 in low pressure CO_2_. (Bottom) Saturation and dry points for Humicap^®^ 2 in low pressure CO_2_

As a result of calibration tests performed at FMI a two-point calibration in Martian conditions is obtained for each Humicap^®^. The calibration curves are presented in Fig. 19 (top), as well as in three different pressures in CO_2_ gas (bottom). Between the dry and saturation curves the Humicap^®^ behaviour is expected to be close to linear within ±10% RH range based on Humicap^®^ characterization in ambient conditions.

Based on the environmental and calibration tests performed at FMI the Humicap^®^ sensor performance and overall HS performance are at the expected level and meet the requirements. This calibration presented here is based on the calibration performed so far, but does not represent the final calibration of the HS. A test campaign with the ground reference model is planned for the Mars Simulation Facility and Planetary Analog Simulation Laboratory (PASLAB) at DLR, and the results will be used for the final calibration of the HS.

### HS Operations Concept

#### Commanding

The commanding concept of the HS is the same as that of the PS (see Sect. 3.4.1), but HS has only one transducer.

#### Modes

HS is operated mostly in the same way as PS, but HS only has “nominal” mode, where all channels of the oscillator are read one by one within 1 s or 2 s, depending on the chosen measurement frequency. Each channel is integrated using the equal amount of reference clock pulses.

As with PS, the ASIC of HS is controlled by FPGA code, and raw data produced by the oscillator is stored in the FPGA registers before transferring them to the ICU. PT1000 resistance is read directly by the ICU.

#### Expected Usage

The HS is expected to be mostly used in one mode: nominal mode with 1 Hz measurement frequency. The length of the HS operation window (power on – power off) can be used to avoid any electronics heating effect: the operating cycles can be for example 5 s on – 5 min off (the off periods can also be longer depending on the desired time resolution, and power and data usage of the HS), which will keep the HS close to the ambient temperature and will allow it to capture small changes in relative humidity.

### HS Data Format

As with PS, the raw output of the humidity sensor transducer is the number of clock pulses corresponding to the oscillating frequency of each channel. In addition, the temperature of PT1000 temperature sensors inside the Humicap^®^ chips is measured by ICU every second. Depending on the chosen measurement frequency, a data packet containing the raw values of each measured oscillator channel and the resistance of PT1000 is read by the ICU every 1 or 2 seconds and delivered to ground as part of MEDA telemetry packets. All data processing of raw values is done on ground.

## Thermal Infrared Sensor (TIRS)

### Introduction, Performance and Scientific Objectives

Using five different wavelength channels, TIRS is an infrared (IR) radiometer which will measure the downward and upward long wave (LW) thermal IR radiation at the surface, the reflected short wave (SW) solar radiation at the surface, the surface brightness temperature, and the air temperature at ∼40 m altitude (Table 10).

**Table 10:** Description and performance of TIRS. An hemispherical FoV and Stefan-Boltzmann emission have been assumed to define the dynamic range of IR1, IR3 and IR4. The variation in the accuracy of IR1, IR3 and IR4 depends on the thermal scenario. IF and BSS stand for interferometric and black silver smoke, respectively

Channel	IR1	IR2	IR3	IR4	IR5
Purpose	Downward	Air Temp	Upward	Upward	Ground
	LW	(∼40 m)	SW	LW	Temp
Band (μm)	6.5–30	14.5–15.5	0.3–3	6.5–30	8–14
Field of View (^∘^)	±20 H	±20 H	±20 H	±20 H	±20 H
	±10 V	±10 V	±10 V	±10 V	±10 V
Pointing	+35	+35	-35	-35	-35
Angle (^∘^)					–
Dynamic Range	3.5–180	173–293 K	0–230	50–420	173–293 K
	W/m^2^		W/m^2^	W/m^2^	
Accuracy	±(1.2–6.9)	±2.83 K	±(3.7–9.6)	±(0.9–3.3)	±0.75 K
	W/m^2^		W/m^2^	W/m^2^	
Resolution	±0.18 W/m^2^	±0.45 K	±0.1 W/m^2^	±0.13 W/m^2^	±0.08 K
Filter Substrate	Si	Ge	Quartz	Si	Si
Absorber	IF LW	IF LW	BSS	IF LW	IF LW
Fillgas	Krypton	Krypton	Krypton	Krypton	Krypton

TIRS is the first in-situ Martian IR radiometer including upward- and downward-looking channels. IR1, IR3 and IR4 will provide the first measurements of their kind from the surface of Mars, while IR2 and IR5 will complement and extend measurements of surface brightness temperature taken by the MSL/REMS (Sebastian et al. 2010) and InSight/HP3 (Spohn et al. 2018; Kopp et al. 2016) instruments, and of air temperature taken by the Miniature Thermal Emission Spectrometer (Mini-TES) onboard the MER rovers (Smith et al. 2004, 2006; Spanovich et al. 2006), respectively.

In combination with MEDA/RDS, TIRS will allow the first in-situ quantification of the surface energy budget (SEB) on Mars. This will be critical to achieve MEDA’s objective: “*validate and improve the predictive capabilities of models of the near surface environment on Mars*”. From global to local, the predictive capability of numerical models strongly depends on the simulated SEB. This is because the near-surface environment and the conditions in the shallow subsurface are largely controlled by the energy available at the surface (Savijärvi et al. 2019; Pla-Garcia et al. 2016; Read et al. 2014). Given the low near-surface air density on Mars (∼10^−2^ kg m^−3^, two orders of magnitude lower than on Earth), the SEB is mostly determined by a radiative balance between the upward and downward SW and LW radiation at the surface ((Martinez et al. 2014) and references therein). These four terms will be measured by TIRS and RDS, allowing for an accurate estimation of the SEB.

Measurements by the TIRS IR3 channel and the panchromatic channel of the RDS will allow the determination of the surface albedo at spatial scales of a few m^2^. Based on predictions from General Circulation Models (GCMs), changes in albedo strongly affect wind circulation, dust transport and the feedback between these processes and the Martian climate (Fenton et al. 2007; Vincendon et al. 2009). On a global scale, this quantity has been inferred from telescopic (Bell and Ansty 2007) and orbital (Christensen et al. 2001; McGuire et al. 2008; Wolff et al. 2006) radiance measurements at spatial scales of a few km/pixel. At local scale, the albedo has been inferred from radiometric analyses of images taken by rovers and landers (Bell et al. 2008; Smith et al. 2009). At both scales, the surface of Mars has typically been considered Lambertian (radiance equally reflected in any angle) to simplify the determination of albedo. Given the geometric characteristics of TIRS and RDS and the variations in the slope of the terrain to be traversed by the Perseverance rover, the solar radiation will be measured for a variety of incoming and reflected pairs of angles. Testing the Lambertian approximation will increase the predictive accuracy of surface characteristics of Mars from orbit by analysis and comparison between orbital and surface observations, as albedo estimations from orbital measurements typically assume a Lambertian albedo.

Another key geophysical property of the terrain that will be determined from TIRS and RDS measurements is the thermal inertia. From seasonal to diurnal time scales, this quantity regulates the thermal amplitude at the surface and in the shallow subsurface. Thermal inertia has typically been obtained via numerical models that solve the heat conduction equation at the surface (Hamilton et al. 2014; Martinez et al. 2014; Vasavada et al. 2017). Using simulated values of the SEB as a boundary condition, these models calculate the thermal inertia by best-fitting their output with telescopic, orbital or in-situ measurements of ground temperature (Kieffer et al. 1977; Fergason et al. 2012; Vasavada et al. 2017). Since TIRS and RDS will provide values of the SEB and ground temperature around the clock, accurate estimations of thermal inertia values will be straightforwardly obtained by solving the heat conduction at the soil. These values will improve predictive capabilities of numerical models, and will provide ground truth to satellite estimations performed at larger spatial resolutions.

TIRS’ IR1 and IR5 measurements will also be used for the indirect detection of water ice clouds at nighttime, complementing daytime detection from other M2020 instruments such as SuperCam and Navcam. As an example, (Vasavada et al. 2017) showed that the seasonal evolution of the modeled and measured nighttime ground temperatures at the MSL landing site diverge from each other during the winter of each year, reaching a maximum deviation of 10 K near the peak of southern winter in Martian Year 33. By adding an extra term to the simulated down-welling LW flux, consistent with a heightened presence of water ice clouds above Gale crater, (Vasavada et al. 2017) obtained a better fit to the measured diurnal minimum ground temperature. Since TIRS will simultaneously measure ground temperatures (IR5) and down-welling LW fluxes (IR5), indirect detection of nighttime water ice clouds should be possible (e.g. Wilson et al. 2007).

Additional TIRS investigations include the determination of the vertical profile of temperature using TIRS’s IR2 (with a weighting function centered at ∼40 m) and IR5 (ground) measurements in combination with ATS (∼1.6 m) measurements. These profiles will complement those retrieved from Mini-TES onboard the MER rovers (Smith et al. 2004). Furthermore, simultaneous measurements of horizontal and vertical wind speed by the WS will allow the estimation of sensible heat fluxes, which, despite their relatively weak impact in the SEB (Martinez et al. 2014), control the formation of convective vortices and dust devils (Newman et al. 2019).

### Design Description

TIRS is composed of the sensor head and the electronic conditioning board, which is located in MEDA’s ICU. These two elements are connected through a 3–4 m long harness. The sensor head has a mass of 97 g and dimensions of 58×63×58 mm^3^. It is attached to the RSM at 1.5 m above the surface, at an orientation of 75^∘^ in the horizontal plane with respect to Perseverance’s Z axis (Fig. 14 -left-). The FoV of the downward-looking channels (Table 10) covers an area of about 3 m^2^, located ∼3.75 m away from the RTG. This area is big enough to ensure proper signal-to-noise ratio, small enough to minimize large heterogeneities of the terrain, and distant enough to mitigate thermal contamination from the RTG.

**Fig. 20:**
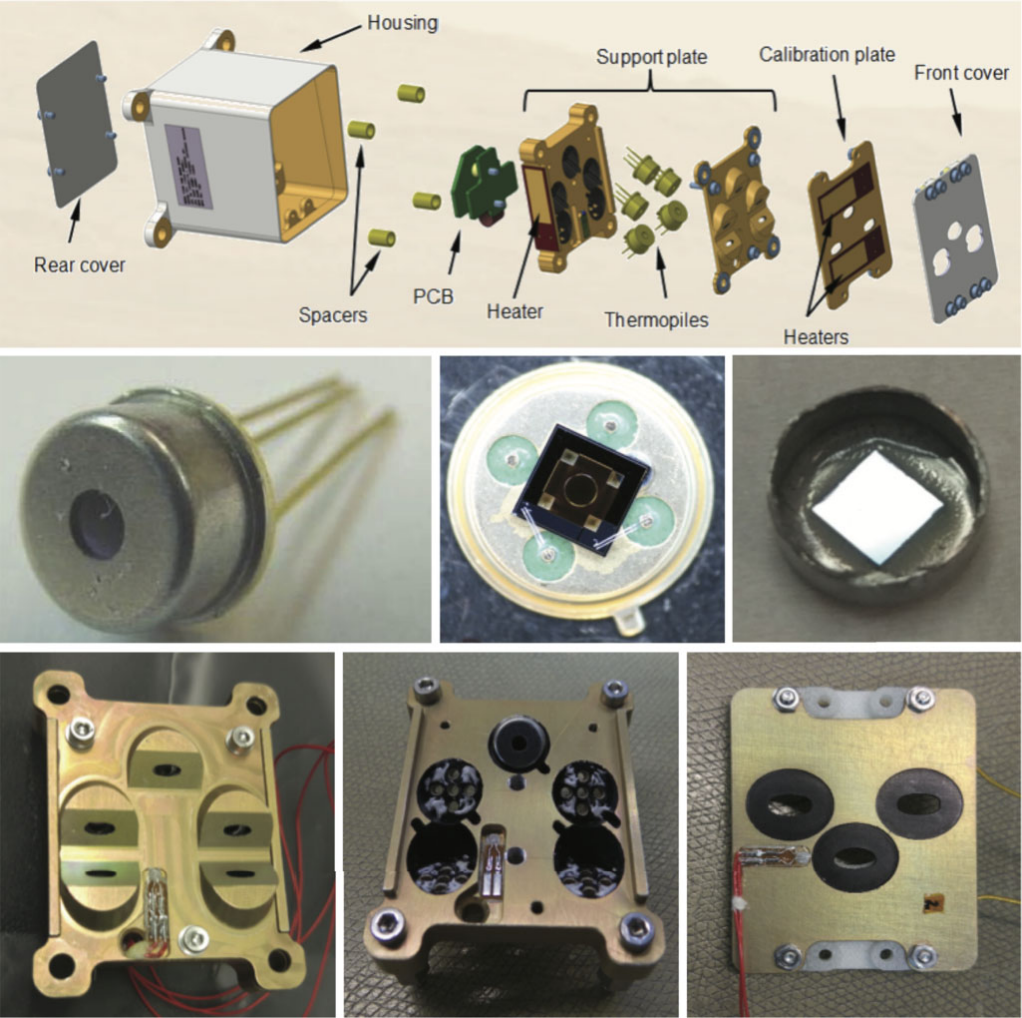
(Top) TIRS exploded 3d drawing showing the components of the sensor head: the housing, the support place, and the calibration plate. From Perez-Izquierdo et al. (2018). (Middle) (Left) TIRS thermopiles with 100 n-bismuth-antimony/p-antimony (Bi0.87Sb0.13/Sb) thermocouples with a Seebeck coefficient of 135 μV/K each. (Middle) Stain-steel socket and thermopile chip (Credit IPHT). (Right) Thermopile filter glued to the nickel cap. From Sebastian et al. (2020). (Bottom) (Left) The support plate rear structure during thermopiles gluing. (Middle) The support plate with the front structure assembled. (Right) The calibration plate (inner side). From Sebastian et al. (2020)

The sensor head is composed of three main elements: the housing and its front and rear cover, the support plate, which contains the five thermopiles, and the calibration plate (Fig. 20, top). The housing consists of an external aluminum structure and two fiber-glass (FR4) covers, back and front. It provides a mechanical chassis that attaches to the RSM and ensures thermal insulation from the environment. To reduce the solar heat loads, the external surfaces are painted using the MAP^®^ SG121 white paint with low absorbance (∼0.2). The housing also includes a single connector MDM31s type to link the sensor head with the ICU.

TIRS uses five TS-100 thermopiles from the Leibniz Institute of Photonic Technology in Jena, Germany (Fig. 20, middle-left) (Leibniz-IPHT 2020). They were selected due to their high heritage from REMS (Sebastian et al. 2010) and MUPUS, the multi purpose sensor package onboard the Rosetta lander Philae (Spohn et al. 2007). These thermopiles measure the radiative heat exchange between the observed targets and the transducer’s sensing elements at temperatures ranging from 138 to 313 K (Graf et al. 2007). The incoming radiation is sensed by a 1 mm^2^ circular absorber (hot junction). A standard TO-5 stainless steel package with 5 pin socket was used to mount the thermopiles’ chips, while redundant bond wires were used to establish electrical connection to the terminals (Fig. 20, middle-middle). Aperture holes were laser-cut into the TO-5 nickel caps and the thermopiles’ IR filters were glued in the inner side (Fig. 20, middle-right). Both chip and filters were glued using MASTERBOND^®^ EP29LPSP, a cryogenic glue that extends the operational range below 138 K. In addition, the thermopiles were encapsulated using Krypton fill gas, which has a boiling point (121 K) well below TIRS’ operational range.

The thermopiles’ sensing element coatings (absorbers) were selected based on their spectral response and endurance (Table 10). Interferometric long wave absorbers (IF LW) were used to avoid undesired transparent windows at twice the wavelength of their optical window for Silicon filters (IR1, IR4 and IR5), and above 40–50 μm for Germanium filters (IR2). A black silver smoke (BSS) absorber was selected for IR3 because no IF LW absorbers with a flat response within 0.3–3 μm were available. Unlike IF LW absorbers, BSS absorbers degrade in the presence of humidity when exposed to temperatures above 358 K (Sebastian et al. 2010), which resulted in a limitation in the sensor testing temperatures during instrument qualification and acceptance tests.

The five TIRS detectors are located inside the support plate, forming a sandwich structure between the front and rear mechanical pieces (Fig. 20, bottom-left and -middle). The support plate is intended to minimize the appearance of thermal gradients in the thermopiles’ package and to perform in-flight calibrations. It is composed of two mechanical pieces made of aluminum which are insulated from the housing using low thermal conductivity spacers made of FR4 and a flex-rigid PCB for electrical connection. Temperature reference measurements are provided by two redundant PT1000-type RTDs glued to the rear and front structures. Two Kapton film heaters were glued to the lateral side to provide power up to 0.8 W. To keep the support plate temperature stable and above ambient temperature during the in-flight calibration mode (Sect. 5.4.3), a PI controller and a PWM driver are used to provide power. The support plate’s internal surfaces, where the IR detectors are located, are inorganic black anodized, while the external surfaces are all chromate conversion coated (Alodine 1200), in order to minimize thermal losses.

The calibration plate (Fig. 20, bottom-right) is made of aluminum and was designed to meet the following criteria: (1) to conform the target FoV to ±20^∘^ in the horizontal and ±10^∘^ in the vertical by partially obstructing the thermopiles’ FoV, (2) to protect the thermopile optics from dust deposition, and (3) to serve as an in-flight calibration target. For this last purpose, the calibration plate includes two Kapton heaters commanded on/off (dissipating up to 0.8 W), as well as an RTD temperature sensor. To increase the emissivity of the calibration target (up to ∼0.9), and to prevent biological contamination, an inorganic black anodized treatment was selected for the calibration plate area sensed by the thermopiles. To reduce conductive and radiative coupling between the support plate and the rest of the instrument, the calibration plate is attached to the front cover of the housing, and all the internal surfaces are coated with chromate conversion (low emissivity of ∼0.08).

The electronic design includes an ultra-low offset and zero drift chopper-stabilized operational amplifier. A low-frequency offset estimation system based on mechanical relays was implemented to measure thermopile voltage with positive and negative polarity, thus removing amplifier offset uncertainties. Finally, two calibration channels were included to compensate offset and gain errors.

### Thermopile Mathematical Model

**Fig. 21:**
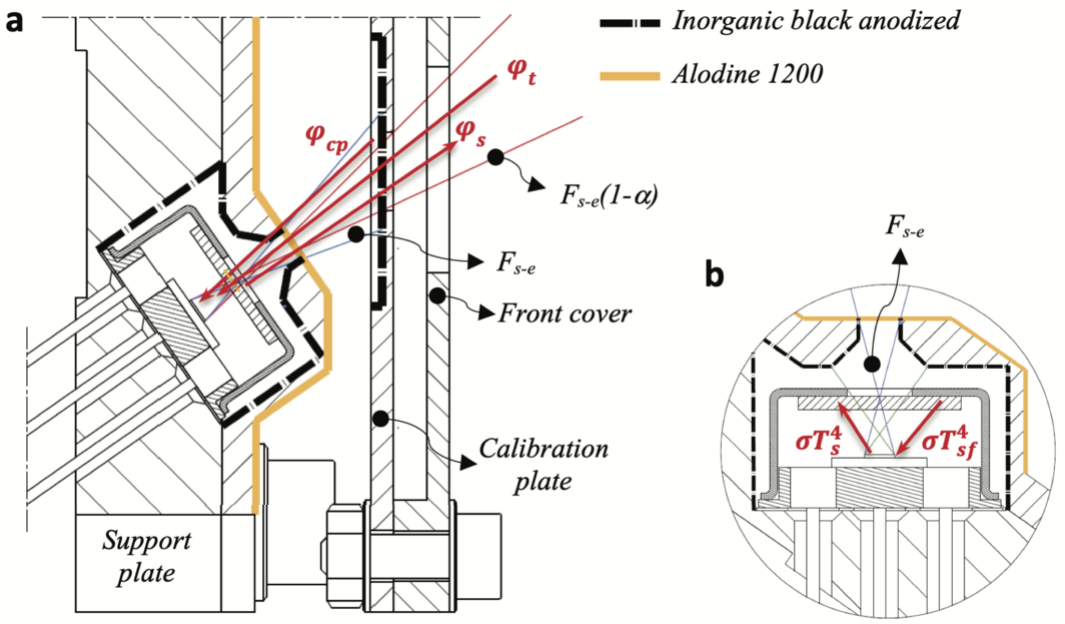
(Left) Thermopiles external IR flux diagram. (Right) Thermopiles internal IR flux diagram. From Sebastian et al. (2020)

The mathematical model for the thermopiles is given by:

11 $$ \begin{aligned} V_{\mathit{out}} = {} & S(T_{s})A_{s}[F_{s-e}\alpha \varphi _{t}+F_{s-e}(1- \alpha )\varphi _{cp}+F_{s-sp}\varphi _{sp-i}-(F_{s-e}+F_{s-sp}) \varphi _{s}] \\ & + S(T_{s})A_{s}(1-F_{s-e}-F_{s-sp})(\sigma T_{sf}^{4}-\sigma T_{s}^{4}) \end{aligned} $$

where $V_{\mathit{out}}$ is the thermopile output voltage (V), $S$ is the thermopile’s responsivity (V/W), $A_{s}$ is thermopile detector area (m^2^), $T_{s}$ is the thermopile detector temperature reference (K), $F_{s-e}$ is the thermopile detector external view factor, $F_{s-sp}$ is the thermopile detector-support plate view factor, $\alpha $ is the portion of the thermopile detector external view factor covered by the target, $(1-\alpha )$ is the portion covered by the calibration plate, $\varphi _{t}$ is the target radiosity (emitted + reflected; W/m^2^), $\varphi _{cp}$ is the calibration plate radiosity (emitted + reflected; W/m^2^), $\varphi _{s}$ is the thermopile detector radiosity (emitted + reflected; W/m^2^), $\varphi _{sp-i}$ is the support plate internal surface radiosity (emitted + reflected; W/m^2^), $\sigma $ is the Stefan-Boltzmann constant, and $T_{sf}$ is the thermopile detector temperature (front part; K). The first term on the right-hand side represents the radiative flux exchange with the external elements, target, and calibration (Fig. 21, left), while the second term represents the thermopile package inner flux exchange (Fig. 21, right).

The mathematical model can be simplified for channel IR3. Given operational temperatures and its filter bandpass (Table 10), the IR flux received from the calibration plate ($\varphi _{cp}$) and leaving the thermopile ($\varphi _{s}$) can be neglected for this channel. In this case, Eq. (11) reduces to:

12 $$ V_{\mathit{out}}=S(T_{s})A_{s}F_{s-e}\alpha \varphi _{t}+ S(T_{s})A_{s}(1-F_{s-e}-F_{s-sp})( \sigma T_{sf}^{4}-\sigma T_{s}^{4}) $$

The output from each channel is obtained by solving Eqs. (11) and (12) for the quantity of interest $\varphi _{t}$. For channels IR2 and IR5, the target temperature $T_{t}$ is simply obtained using Planck’s law $\varphi _{t}=\sigma T_{t}^{4}$. To obtain $\varphi _{t}$, the rest of quantities in Eqs. (11) and (12) must be measured or subjected to calibration, as shown in the following section.

### Calibration

**Fig. 22:**
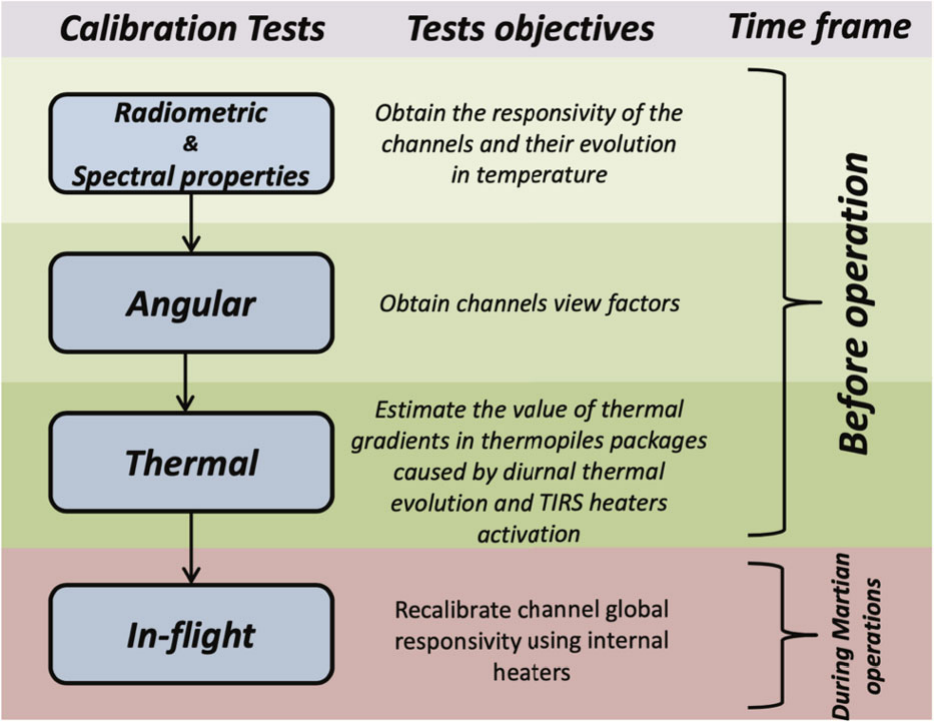
Sequential steps of TIRS’ calibration plan. From Sebastian et al. (2020)

The TIRS calibration plan consisted of four sequential steps shown in Fig. 22. A detailed explanation of the radiometric and angular tests is given in Sebastian et al. (2020), while details of the thermal tests are given in Sebastian et al. (2021). Here, we show the main results for these tests and introduce the in-flight calibration.

#### Pre-Flight Radiometric and Angular Calibration

Table 11 shows the sequential radiometric and angular tests that have been performed to calculate the value of unknown parameters in Eqs. (11) and (12). Next, we summarize the main results of each of these tests.

**Table 11:** Sequential steps of the TIRS radiometric and angular calibration plan. C and S stand for calibration and support, while av stands for average

Test purpose	Level	Blackbody temp. [K]	Solar simulator irradiance [W/m^2^]	Sample holder [K]	Test conditions
Spectral responsivity, *χ*(*λ*)	Filters sample	–	–	∼298	Earth atmosphere
C&S plate av. emissivities, $\epsilon _{cp}$ and $\epsilon _{sp}$	Surfaces samples	–	–	∼298	Earth atmosphere
Relative responsivity vs. temp.	Support plate	∼353, 368 383, 398 413	–	∼143, 153, 323	Vacuum^∗^
Absolute responsivity, $S(T_{s})$	Support plate (IR1, IR2, IR4, IR5)	∼323, 343 363, 383 403	–	∼313	Earth^∗^ atmosphere
Absolute responsivity, $S(T_{s})$	TIRS IR3	–	0, 12, 24 30	298	Earth^∗^ atmosphere
Target relative FoV, *α*	TIRS (IR1, IR2, IR4, IR5)	∼313, 323 333, 343	–	∼298	Earth^∗^ atmosphere
IR3 external FoV, $F_{s-e}.\alpha $	TIRS (IR3)	–	∼30	∼298	Earth^∗^ atmosphere

^∗^Negligible absorption by the Earth atmosphere given the calibration geometry (short optical path)

##### Thermopiles Spectral Responsivity

**Fig. 23:**
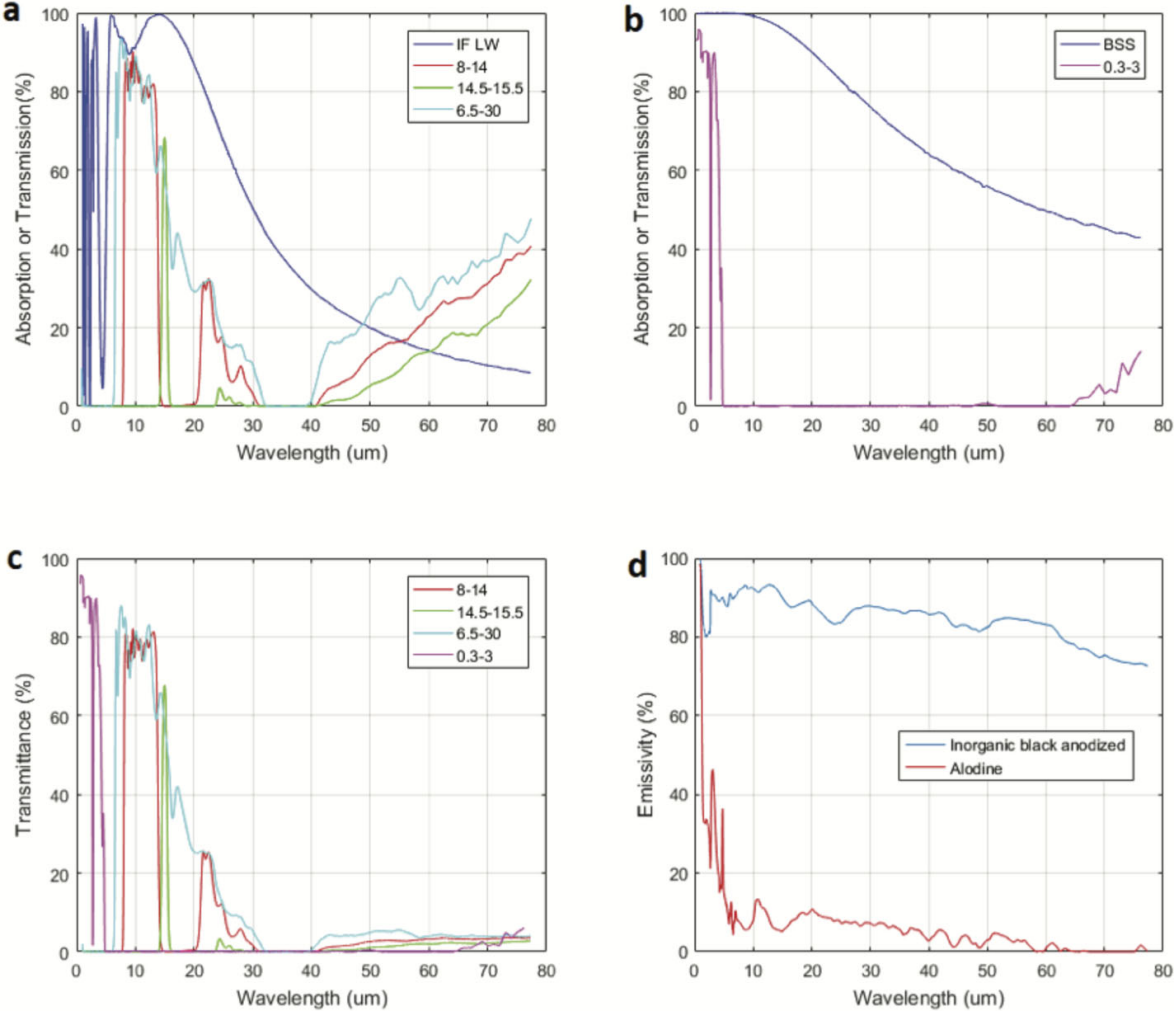
(**a**) Spectral transmittance of the 8–14 μm (IR5), 14.5–15.5 μm (IR2), and 6.5–30 μm (IR1 and IR4) filters and spectral absorptance of the IF LW absorber at room temperature. (**b**) Spectral transmittance of the 0.3–3 μm (IR3) filter and spectral absorptance for the BBS absorber at room temperature. (**c**) Spectral responsivity of the 8–14 μm, 14.5–15.5 μm, 6.5–30 μm, and 0.3–3 μm thermopiles. (d) Spectral diffuse emissivity of TIRS aluminum surface treatments (inorganic black anodized and chromate conversion). From Sebastian et al. (2020)

The thermopile spectral responsivity, $\chi (\lambda )$, is obtained as the product of the filter spectral transmittance and the absorber spectral absorptance. To calculate the thermopiles’ filters spectral transmittance, a Thermo Nicolet^®^ Nexus FTIR spectrometer operating in the range 0.43–80 μm was utilized. The filters were placed perpendicular to the light beam in a 10×10 mm^2^ sample holder at room temperature. Results of the filter spectral transmittance and spectral absorption of the IF LW coatings of channels IR1, IR2, IR4 and IR5 are shown in Fig. 23a, while those of the IR3 BBS coating are shown in Fig. 23b. The corresponding spectral responsivity is shown in Fig. 23c for each channel.

The effect of changes in temperature and radiation incidence angle in the spectral response was assessed by performing additional tests (Sebastian et al. 2020). To account for this twofold effect, a characterization of the responsivity as a function of temperature and a calculation of the FoV integration effect were performed. More details of these calculations are given below.

##### Calibration and Support Plate Average Emissivities

Table 12 shows the average emissivities of the calibration and support plate, $\epsilon _{cp}$ and $\epsilon _{sp}$. These values were obtained by integrating the equation:

13 $$ \epsilon _{x}= \frac{\int _{0}^{\infty }\epsilon _{x}(\lambda ).\chi (\lambda ).\frac{2\pi hc^{2}}{\lambda ^{5}(\exp (hc/\lambda kT_{x})-1)}}{\int _{0}^{\infty }(\chi (\lambda ).\frac{2\pi hc^{2}}{\lambda ^{5}(\exp (hc/\lambda kT_{x})-1)}} $$

where $\epsilon _{x}$ are the surface spectral emissivities shown in Fig. 23d (both for the calibration and support plate), and $\chi (\lambda )$ is the channel spectral response shown in Fig. 23c. The surface spectral emissivities, in turn, were obtained using Kirchhoff’s law as $\epsilon (\lambda )=1-r(\lambda )$, where the spectral diffuse reflection, $r(\lambda )$, was measured at room temperature from 0.43 to 80 μm by a Thermo Nicolet^®^ Nexus FTIR spectrometer with a diffuse reflection attachment from HARRICK^®^.

**Table 12:** Experimental data for the calibration and support plate average emissivities. Data for the IR3 channel is not included because the calibration plate radiosity can be neglected

Channel	$\epsilon _{cp}$	$\epsilon _{sp}$
IR1&IR4	0.908±0.007	0.0820±0.0004
IR2	0.896±0.003	0.0540±0.0004
IR5	0.917±0.006	0.0860±0.0006

##### Thermopiles Relative Responsivities vs. Temperature

**Fig. 24:**
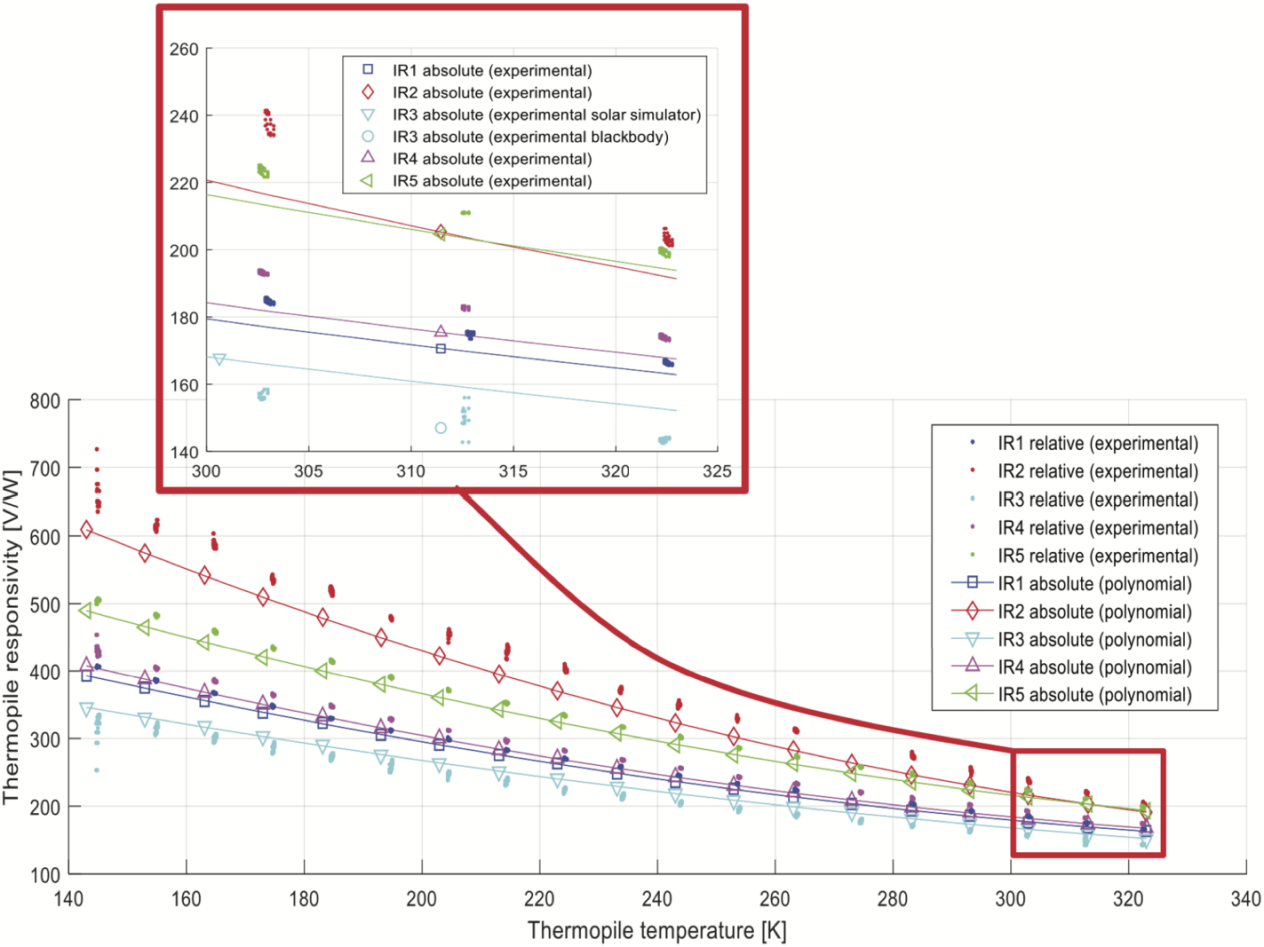
TIRS Proto Flight Model relative (filled circles) and absolute (empty symbols) responsivities as a function of temperature. Colored lines represent a polynomial fit to absolute responsivities. (Insert) Experimental absolute responsivities obtained in the range of temperature shown in Table 13. From Sebastian et al. (2020)

Experimental relative responsivities are shown in Fig. 24 (filled circles) as a function of temperature for each channel. Responsivity values decrease with temperature, consistent with thermopile fill gas thermal conductivities (Spohn et al. 2018). The scatter in the values is caused by measurement noise and small spatial gradients in the thermopiles’ packages (<0.015 K). The largest scatter correspond to IR2 and IR3 channels, which have the narrowest bandpass filters and therefore the smallest signal-to-package gradient ratio (Sebastian et al. 2011).

##### Thermopiles Absolute Responsivities

The experimental values obtained from the relative responsivity tests were subjected to uncertainties caused by the cryostat window transmittance ($\chi _{w}$). As a consequence, a second calibration step in which the cryostat window was removed for IR1, IR2, IR4 and IR5, and a specific setup was used for IR3, was required to obtain reliable values for the absolute responsivity (Sebastian et al. 2020).

Figure 24 shows the results of this second set of tests, where the empty symbols represent the values of the absolute responsivity. As in the relative responsivity tests, the scatter was caused by small spatial gradients in the thermopile’s package. Absolute responsivity curves, represented by colored lines in Fig. 24, were obtained following the next steps. First, a polynomial fit to the relative responsivities was performed as a function of temperature. Second, a correction factor was calculated by dividing the absolute responsivity at the tested temperature by the relative responsivity given by the polynomial fit. Finally, experimental values of the relative responsivities were multiplied by their corresponding correction factors, resulting in new polynomial fits for the absolute responsivity (Table 13).

**Table 13:** TIRS PFM calibration results of the absolute responsivity performed at different temperatures (second column) and polynomial fits for each channel (third column). In both cases, the temperature is expressed in ^∘^C

Channel	S @ different Temps	S(Ts)
	[V/W]	[V/W]
IR1	170.6 at 38.4 ± 0.21^∘^	0.0036⋅($T_{s}$-273)^2^-0.9931⋅($T_{s}$-273)+203.5
IR2	205.4 at 38.4 ± 4.64^∘^	0.0067⋅($T_{s}$-273)^2^-1.7865⋅($T_{s}$-273)+264.1
IR3	165.8 at 27.6 ± 1.38^∘^	0.0024⋅($T_{s}$-273)^2^-0.8720⋅($T_{s}$-273)+188.0
IR4	175.3 at 38.5 ± 0.26^∘^	0.0039⋅($T_{s}$-273)^2^-1.0230⋅($T_{s}$-273)+208.9
IR5	204.5 at 38.5 ± 0.32^∘^	0.0042⋅($T_{s}$-273)^2^-1.3045⋅($T_{s}$-273)+248.4

##### Target Relative FoV

Values of the target relative field of view, $\alpha $, and thermopile detector-target view factors, $F_{(s-e)}.\alpha $, were calculated following a dedicated calibration test described in (Sebastian et al. 2020). The results of these tests are summarized in Table 14.

**Table 14:** TIRS PFM calibration results of the relative field of view, $\alpha $, and thermopile detector-target FoV, $F_{(s-e)}\alpha $

Channel	*α*	$F_{s-e}\alpha $
IR1	0.575±0.0017	–
IR2	0.480±0.066	–
IR3	–	0.0336
IR4	0.584±0.0012	–
IR5	0.559±0.0008	–

The non-modeled angular change in the thermopiles’ spectral response was noticeable in the calibrated value of $\alpha $. The calibration plate shapes the target FoV ($\alpha .F_{s-e}$) limiting the external FoV ($F_{s-e}$) from ±30^∘^ in the horizontal and ±15^∘^ in the vertical to ±20^∘^ and ±10^∘^, respectively. Since the calibrated responsivity, $S(T_{s})$, was more strongly affected at high angles (with responsivities higher than the nominal), the retrieved value for $\alpha $ was smaller than that theoretically-predicted from geometrical analyses (∼0.55). This way, the calibrated value of $\alpha $ compensates the non-modeled angular change in the thermopiles’ spectral response.

##### IR3 Target FoV

**Fig. 25:**
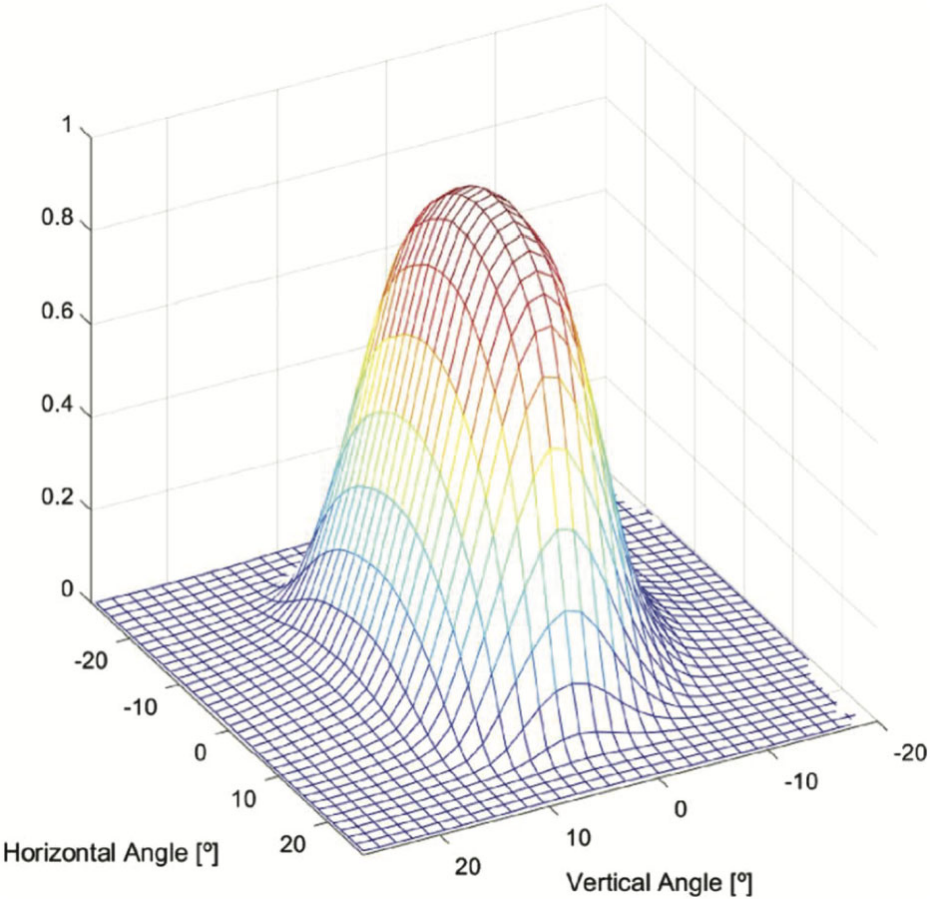
TIRS PFM IR3 normalized thermopile response versus FoV angle. From Perez-Izquierdo et al. (2018)

A dedicated calibration test was performed to calculate the IR3 target FoV, $\alpha .F_{s-e}$. Using a experimental setup described in Sebastian et al. (2020), the TIRS PFM was tilted ±25^∘^ in the horizontal and ±20^∘^ in the vertical in steps of 1^∘^ to form a data matrix. Horizontal ($\gamma $) and vertical ($\beta $) angles at which the thermopile voltage was maximum were taken as the center of the FoV, and then the IR3 angular response, $R(\gamma ,\beta )$, was normalized to that value (Fig. 25). Finally, the target FoV was calculated by comparison with the cosine response of an ideal detector of $\pi $ srad FoV as:

14 $$ \alpha .F_{s-e}= \frac{\sum _{\beta =-90^{\circ} }^{\beta =90^{\circ} }(\sum _{\gamma =-90^{\circ} }^{\gamma =90^{\circ} }R(\gamma ,\beta ))}{\sum _{\beta =-90^{\circ} }^{\beta =90^{\circ} }(\sum _{\gamma =-90^{\circ} }^{\gamma =90^{\circ} }(\cos {(\gamma )}.\cos {(\beta )})} $$

with the results shown in Table 14.

##### Uncertainties in the Radiometric and Angular Calibration

Systematic errors and corresponding uncertainties associated with the electro-optical calibration of TIRS are caused by three factors: uncertainties in the calibration target IR flux, uncertainties in the thermopiles’ responsivity calibration, and differences between simulated and actual Martian environmental conditions (mainly associated with target temperatures).

Table 15 shows the estimated values corresponding to each of these uncertainties. Details of their calculations are given in Sebastian et al. (2020). The last column summarizes the total uncertainty of the electro-optical calibration, where the different uncertainties have been added quadratically, assuming uncorrelated probability distributions for all of errors.

**Table 15:** Calibration test uncertainties values

Channel	Cal Target IR Flux (1*σ*,%)	Thermopile responsivity (1*σ*,%)	Cal Target vs. Mars filters temp ($\sqrt{6 }$*σ*,%)	Cal Target vs. Mars filters angle ($\sqrt{6 }$*σ*,%)	Total (1*σ*,%)
IR1	1.51	0.12	3.59	2.43	2.33
IR2	1.51	2.26	2.47	2.54	3.08
IR3	2.23	0.83	–	–	2.38
IR4	1.51	0.15	3.59	2.43	2.33
IR5	1.51	0.16	3.59	2.43	2.33

#### Pre-Flight Thermal Calibration

The appearance of thermal gradients in the package of IR detectors constitutes one of the most important and challenging sources of uncertainties in their calibration plans (Liess et al. 2006, 2009; Meca et al. 2002). These gradients arise, for instance, when the package is subjected to direct thermal contamination from the Sun or thermal control systems in which they are embedded, or from wind gusts in open-air applications (Meca et al. 2002; Grott et al. 2017).

The TIRS’ thermopile thermal gradient, represented by the second term on the right-hand side of Eq. (11), cannot be measured given design constraints and therefore it needs to be estimated. Here we summarize the calibration plan and tests performed to estimate this gradient, and thus compensate the TIRS measurements for its effect.

##### Thermopile Thermal Gradient Estimators

A Thermal Mathematical Model (TMM) was used to recreate the thermal environment inside TIRS. By analyzing simulated values of the thermopiles’ package thermal gradient, $T_{sf}-T_{s}$, and its correlation with a few measurable quantities (estimators).

Equation (15) represents the estimator corresponding to nominal operations (Sect. 5.5), where the thermal gradient is obtained using the temperature difference between the calibration and support plate (first right-hand side term), and the support plate temperature derivative (second right-hand side term), through the constants $K$ and $K'$ measured in units of mK/K and mK/K/h.

15 $$ T_{sf}-T_{s}=K(T_{cp}-T_{s}) + K^{\prime }\frac{\partial T_{s}}{\partial t} $$

To calculate thermal gradients during the in-flight calibration mode (Sect. 5.4.3), the TMM was used to simulate two cases in which the calibration and support plate heaters were dissipating 0.8 W for 30 min and 50 min, respectively. Analyses of these simulations showed that while the calibration plate heaters caused a positive gradient (front part hotter than the rear), the support plate heaters caused a negative one (Sebastian et al. 2021). This behavior is accounted for by Eq. (16), where a steady state function, $f_{cp}$, which depends on the spatial gradient between the calibration and support plates, $T_{cp}-T_{s}$, was defined for the in-flight calibration mode using the calibration plate.

16 $$ T_{sf}-T_{s}=f_{cp}(T_{cp}-T_{s}) $$

Similarly, another steady state function, $f_{sp}$, which depends on the power driven to the support plate heater, $\mathit{Power}_{sp}$, and on $T_{cp}-T_{s}$, was defined for the in-flight calibration mode using the support plate (Eq. (17)).

17 $$ T_{sf}-T_{s}=f_{sp}(\mathit{Power}_{sp},(T_{cp}-T_{s})) $$

##### Thermal Calibration Tests and Results

Table 16 summarizes the TIRS thermal calibration plan, which included two different sets of tests to identify the value of the unknown constants in Eq. (15) (nominal mode), and of the functions in Eqs. (16) and (17) (in-flight calibration mode).

**Table 16:** TIRS thermal calibration plan

Test purpose	Target temp. [K]	Sample holder [K]	Support plate heater [W]	Calib. plate heater [W]	Test pressure [mb]
Nominal mode	∼300	286, 293, 300, 307, 314 Profile: ±14 K/h, ±7 K/h	0	0	8
Calibration mode	∼300	286, 293, 300, 307, 314	∼0, 0.2, 0.4, 0.8	∼0, 0.8	8

The tests aimed at characterizing thermal gradients in the nominal mode were performed in the MARTE simulation chamber of the Centro de Astrobiologia in Madrid, Spain (Sobrado et al. 2014). By recreating the thermal conditions shown in Table 16, the evolution of the thermopile package gradient $(T_{sf}-T_{s})$ was computed from Eq. (15) (Sebastian et al. 2021). Then, the coefficients $K$ and $K^{\prime }$ were obtained using a least square algorithm (Table 17).

**Table 17:** Experimental values of $K$ and $K^{\prime }$ in the nominal operation mode. The associated uncertainties are being calculated at the time of this writing, and will be shown in Sebastian et al. (2021)

Channel	*K* [mK/K]	$K^{\prime }$ [mK/K/h]
IR1	13.70	-1.205
IR2	1.93	-1.602
IR3	14.91	-3.861
IR4	16.21	-1.513
IR5	9.21	-1.326

The second set of tests were also performed in the MARTE simulation chamber. The objective was to compensate thermal gradients arising as a result of in-flight calibration algorithm executions by driving power either to the calibration or the support plate. The results from these tests are summarized in Table 18.

**Table 18:** Practical values for package’s gradients and equivalent target temperature or irradiance uncertainties. Note that it has been assumed that the target and thermopiles’ temperatures are the same as the room temperature

Chan.	Nominal mode (Max. gradient/ uncertainty)	Cal. mode (support plate) (Max. gradient/ uncertainty)	Cal. mode (calibration plate) (Max. gradient/ uncertainty)
IR1	±12 mK/±2.37 W/m^2^^a^	-13 mK/-2.57 W/m^2^^a^	+60 mK/+11.85 W/m^2^^a^
IR2	±15 mK/±12.2 K	-5.5 mK/-4.37 K	+7 mK/+5.69 K
IR3	±35 mK/±5 W/m^2^	-42.5 mK/-6.07 W/m^2^	+40 mK/+5.71 W/m^2^
IR4	±14 mK/±2.73 W/m^2^^a^	-40 mK/-7.8 W/m^2^^a^	+36 mK/+7.02 W/m^2^^a^
IR5	±11 mK/±0.58 K	-28 mK/-1.48 K	+32 mK/+1.69 K

^a^This error is calculated assuming Stefan-Boltzmann emission

**Fig. 26:**
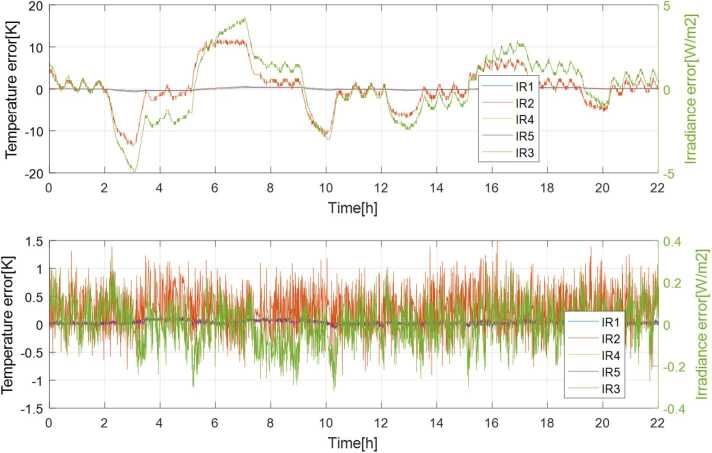
Target temperature and irradiance uncertainty as result of thermal gradients for the nominal operation test data, (top) before applying thermal calibration, and (bottom) after applying thermal calibration. From Sebastian et al. (2021)

An example of the performance of the thermal calibration is given in Fig. 26, which shows the uncertainty introduced by the appearance of thermal gradients before (top) and after (bottom) applying our correction. The uncertainty is significantly reduced for each channel (note the different scale in the Y-axis), particularly for channels IR2 and IR3.

##### Uncertainties in the Thermal Calibration

The limitations imposed by the experimental setup to cover the entire range of operational temperatures introduced uncertainties in the calculation of $K$ and $K^{\prime }$. To estimate these uncertainties, the TMM was used to simulate extreme thermal environments. Analyses of these simulations, shown in Sebastian et al. (2021), indicate that $K^{\prime }$ increases up to 31.4% at the lowest operational temperature for a support plate temperature gradient of 10 K/h. By taking 31.4% as the upper bound, the gradient estimation uncertainty can be obtained for all the channels as $K^{\prime }\times 10(K/h) \times 31.4\%$ (Table 19).

**Table 19:** Uncertainty in the thermopiles package thermal gradient

Channel	Gradient uncertainty due to testing temperature [mK]	Gradient uncertainty due to calibration target error [mK]
IR1	±0.08	±9.13
IR2	±1.61	±0.358
IR3	±4.37	±0
IR4	±0.1	±9.27
IR5	±0.39	±5.73

Although similar uncertainties might affect the thermal gradients during the heating of the support and calibration plates, the in-flight calibration procedure will be executed during the warmest hours of the day, when temperatures are in the range experimentally simulated in our calibration tests.

Finally, the errors associated with the brightness temperature of the chamber calibration target (0.3 K) introduce an uncertainty in the estimation of the thermal gradients. Table 19 shows the equivalent thermal gradient uncertainty for each channel, with the largest values presented corresponding to channels IR1 and IR4 given their larger bandwidth. In the particular case of channel IR3, the radiation from the target and the associated uncertainties are null, since the test was developed in dark conditions.

#### In-Flight Calibration

Airborne dust is expected to build up on the filter of the TIRS’ thermopiles, partially blocking the incoming and outgoing radiation from the detectors. Martian dust has high emissivity and will acquire the same temperature as the TIRS package shortly after being deposited on the filter, limiting its effective area. To account for this effect, Eq. (11) can be modified by introducing a new parameter $\beta $, which represents the part of the FoV not obstructed by dust as:

18 $$ \begin{aligned} V_{\mathit{out}} = {} & S(T_{s})A_{s}\beta [F_{s-e} \alpha \varphi _{t}+F_{s-e}(1- \alpha )\varphi _{cp}+F_{s-sp}\varphi _{sp-i}] \\ & - S(T_{s})A_{s}\beta (F_{s-e}+F_{s-sp})\varphi _{s} \\ & + S(T_{s})A_{s}(1-\beta F_{s-e}-\beta F_{s-sp})(\sigma T_{sf}^{4}- \sigma T_{s}^{4}) \end{aligned} $$

The parameter $\beta $ will be determined during in-flight operations by varying, in a controlled and iterative manner, the temperature of the calibration and support plate using their heaters. First, an initial value of $\beta $ will be considered to solve Eq. (18), both before and during the activation of the heaters. Then, the two values obtained for $\varphi _{t}$ will be compared using an estimator that accounts for the variation of such values as a function of temperature. Similar variations will indicate a correct estimation of $\beta $, while different values will keep the process iterating until an optimal value of $\beta $ is achieved.

### Measuring Strategy

TIRS has been designed to operate in three modes: nominal, high-resolution and in-flight. In the nominal model, TIRS channels and auxiliary RTDs will measure with a frequency ranging from 0.5 to 2 Hz during the first thirty minutes of each hours. In the high-resolution mode, the accuracy of all TIRS channels will be maximized by using a novel low frequency offset estimation system based on mechanical relays. This system allows to measure thermopiles voltage with positive and negative polarity, thus removing amplifier offset uncertainties. Finally, in the in-flight calibration mode, TIRS will be recalibrated following Eq. (18) to obtain $\beta $.

## Wind Sensor (WS)

The MEDA Wind Sensor (WS) consists of two booms placed at about 1.5 m above the base of the rover wheels and rotated an azimuth of 120 degree from each other around the rover RSM.

### WS Science Objectives and Requirements

Near-surface measurements of wind speed and direction provide information on large-scale wind patterns, atmospheric waves, and other phenomena (Hess et al. 1977; Schofield et al. 1997; Holstein-Rathlou et al. 2010; Pla-Garcia et al. 2016; Newman et al. 2017; Banfield et al. 2020; Viúdez-Moreiras et al. 2020). Large-scale wind patterns result from often-complex interactions between the global (Hadley) circulation, flows driven by atmospheric thermal tides, and flows guided by regional and local topography, influenced by the variable atmospheric dust distribution. Winds measured over periods ranging from several sols to several minutes also carry evidence of atmospheric waves, from baroclinic waves generated at higher latitudes to near surface waves generated by shear flows, convection and major topography (Banfield et al. 2020). Winds also provide insight into the amount and nature of daytime convective circulations, including convective vortex passage, and the presence of bores and nocturnal boundary layer jets (Savijärvi and Siili 1993; Schofield et al. 1997; Maria et al. 2006; Banfield et al. 2020). Wind-driven processes at the Martian surface include direct erosion; saltation and the formation of aeolian features such as dunes; and importantly, the lifting of dust (Greeley 2002; Bridges et al. 2017; Sullivan and Kok 2017). Wind also affects atmospheric mixing and controls transport of suspended dust and trace gases (Lian et al. 2012; Gillespie et al. 2020). All of this results in the significant impact of wind speed and direction on what we observe on the surface and in the atmosphere. In addition, due to the relationship between wind and dust lifting, there are likely major feedbacks between near-surface winds, dust storms, and climate (Newman et al. 2002; Bertrand et al. 2020).

Despite the importance of near-surface winds to understanding everything from dust storm onset to aeolian features to risks for robotic and human landed missions, good wind datasets from the Martian surface are rare. Viking Landers 1 and 2 measured wind speed and direction well for 44 and 61 sols, respectively (Hess et al. 1977). A correction to the retrieval algorithm enabled the former dataset to be extended to 350 sols (Murphy et al. 1990). These datasets enabled the first measurement of slope winds (daytime upslope / nighttime downslope flows) at VL1 landing site and tidal wind rotation, and the impact on near-surface winds of the 1977A and 1977B global dust storms. However, high-frequency wind measurements (up to 1 Hz) were recorded only rarely, and to date, only hourly-averaged measurements are publicly available via the PDS Atmospheres node. Two decades later, Mars Pathfinder’s wind sensors (Seiff et al. 1997) again measured the effect of slope on near-surface wind direction (Schofield et al. 1997), but were unable to provide wind speeds due to a problem with the planned method of calibration. The MER rovers carried no meteorological sensors. The Phoenix Lander carried only a mechanical anemometer, a “Telltale” hanging indicator that was imaged to provide estimates of wind speed and direction, for more than 140 sols, including series of high-frequency images able to study atmospheric turbulence at its polar landing location (Holstein-Rathlou et al. 2010).

The Curiosity Rover carried a forerunner of the InSight and MEDA wind sensor booms, but was plagued by difficulties. Damage to the side/rear-facing wind sensor boom at the very start of Curiosity’s mission, likely caused by particles thrown up during landing, meant that winds from the rear of the rover could not be measured with any accuracy. In addition, noise in the remaining (front-facing) boom’s electronics meant that winds could not be measured below a certain temperature, which led to the loss of 8 to 12 hours of winds overnight, depending on season. In combination, this resulted in several data gaps, particularly at night but also in southern summer, when mission constraints resulted in the rover largely facing away from the daytime wind direction. As a result, the REMS wind sensors on Curiosity were not able to measure accurate or unbiased wind speeds and directions over much of their operation period, being necessary to develop specific retrieval algorithms to extract reliable wind data from the raw REMS wind dataset (Viudez-Moreiras et al. 2019a). Curiosity’s wind sensor operation ceased altogether after 2.4 Mars years, when further damage occurred, likely due to aeolian bombardment (Viudez-Moreiras et al. 2019b). Despite this, Curiosity wind measurements confirmed the strong control by Gale crater’s major topography, with local slope flows dominating over regional/global flows in most seasons. Curiosity also made the first measurements of winds in a dune field on another planet (Newman et al. 2017).

The MEDA wind sensors (WS) will enable 1.5 m altitude horizontal and vertical winds in Jezero Crater to be measured at up to 2 Hz frequency as a function of time of sol, season, dust conditions, and location along the rover’s traverse. Continuous monitoring at frequencies of at least 0.5 Hz should be possible, enabling a very long, continuous time series to be obtained over the course of the mission, which will provide an invaluable dataset for the validation and improvement of numerical atmospheric models. The MEDA WS carries double the number of sensor boards than REMS, and will land with one of its booms in a folded and thus somewhat protected position. Hence it has greater redundancy and should prove more resilient, and the electronics have been greatly improved since REMS, as demonstrated by the success of InSight’s TWINS wind sensors (Banfield et al. 2019). The latter are performing well as of over half a Mars year into that mission, although wind speeds and directions are potentially impacted by lander elements such as the solar panels or deck antennae. By contrast, MEDA’s WS have been designed to extend far from the rover body, outside the main region of flow disturbance according to models, minimizing rover influence as much as possible. In terms of the winds themselves, while InSight is stationary on a relatively flat plain, the Perseverance rover will explore a region of far more variable topography. Thus MEDA’s WS will provide accurate and nearly continuous 1 Hz frequency wind data for a range of different local and regional topography, which will shed further light on the relative importance of global and regional winds vs slope winds, as well as a much better characterization of nearby convective vortices through the combination of wind and pressure data, for example. Over the course of an extended mission in particular, the WS would allow detailed model predictions of how winds should vary across the wider Jezero region to be tested.

The location of Jezero Crater (at 77.6^∘^E, 18.4^∘^N) is similar in latitude to the location of Viking Lander 1 (VL1; at 50^∘^W, 22.5^∘^N), enabling comparison with the wave activity measured by VL1 in pressure and wind (Murphy et al. 1990). The higher latitude of M2020 than InSight (located at 135.9^∘^ E, 4.5^∘^ N) will also provide an excellent contrast with InSight’s measurements of pressure and winds, which indicated strong controls on sol-to-sol wind variability in northern winter by baroclinic waves inferred to have traveled from northern high latitudes (Banfield et al. 2020). The dominant wind speeds and directions measured by MEDA will be useful not only for constraining models but also for better interpreting existing wind data and placing them in the context of regional-to-global scale circulations. Jezero crater is far shallower than Gale, hence atmospheric models predict that regional slope flows associated with the NW slopes of Isidis Basin will dominate over those associated with crater topography, by contrast with the strong local slope control seen in Gale for most seasons.

The MEDA WS will characterize the vertical component of the wind for the first time, shedding light on atmospheric turbulence, convection (including dust devils) and their sources through the combined measurements of ground and air temperatures from at different altitudes from other MEDA sensors.

Linking wind measurements to dust removal from MEDA’s Radiation and Dust Sensor (RDS) optics, to vortex-like pressure drops measured by the PS, and to changes in dust properties measured by the RDS, will help to understand the connection between winds, dust devils, and dust lifting from the surface, ideally both in relatively clear periods and during dust storm events.

Estimates of the expected “dust devil activity,” which gives the amount of convective vortex activity expected based on surface sensible heat flux and convective boundary layer depth (Rennó et al. 1998), have been made for both InSight’s landing site and the Jezero crater region using atmospheric predictions from the MarsWRF atmospheric model, as was previously done for Curiosity’s location in Gale crater in Newman et al. (2019). The results of this modeling are presented in Newman et al. (2021) and show that Jezero is predicted to have stronger vortex activity than at the InSight landing site in local summer (when activity peaks for both locations), and to have stronger vortex activity even in local winter than Gale crater experiences at the same time of year (i.e. in local summer in Gale, when the predicted and observed activity there peaks).

Wind measurements will also have strong synergy with other M2020 investigations. By linking wind stress (estimated from wind speed, pressure, and temperature) and observations of sand movement on the surface, they will help to infer the threshold for aeolian activity. This will not only help to explain currently active aeolian features all across Mars, but will help connect current conditions to potential past climates needed to explain the aeolian record as preserved by the characteristics of depositional bedforms and erosional features (M2020 Objective A). Finally, wind measurements will also help to constrain the weathering and preservation potential of a possible cache sample (Objective C.1).

To capture all flows of interest—from diurnal wind patterns, which may not require high frequency sampling, to turbulence and wind gusts, which require high accuracy and high frequency measurements up to high wind speeds—we identified MEDA’S L4 requirements corresponding to the WS as shown in Table 20 below.

**Table 20:** WS Requirements

Investigation	Requirements	Performance
MEDA shall characterize the atmospheric wind speed and direction around the rover on the surface of Mars	MEDA shall characterize the vertical component of the wind at the sensor location in the range of 0 to 10 m/s.	These requirements have not been tested, but the design includes 4 boards per boom for vertical component measurement,more than the REMS and Insight sensors, to make the vertical component requirements achievable.
	MEDA shall characterize the vertical component of the wind at the sensor location with an accuracy of +/-1 m/s in speed.	
	MEDA shall characterize the vertical component of the wind at the sensor location with a resolution of at least 0.5 m/s in speed.	
	MEDA shall characterize the horizontal component of the wind at the sensor location in the range of 0 to 40 m/s.	The range requirement has been verified for temperature 25 ^∘^C, for -130 ^∘^C the equivalent tested range is up to 11 m/s.
	MEDA shall characterize the horizontal component of the wind at the sensor location with an accuracy of +/- 1 m/s from 0-10 m/s and 10% above 10 m/s in speed due to the sensor, and the same due to the rover.	This requirement has not been tested, but in the TWINS instrument (using a prior and more basic version of the instrument) the error of the inverse algorithm was less than 10%, This requirement has not been tested, but in the TWINS instrument (using a prior and more basic version of the instrument) the error of the inverse algorithm was less than 10%, thus meeting the MEDA requirement. MEDA doubles the number of boards to be used in the algorithms and increases the power available in the ASIC, so it is expected that the improved design over TWINS will enable to reduce the error after tunnel calibration.
	MEDA shall characterize the horizontal component of the wind at the sensor location with an accuracy in the wind direction of +/-15 deg due to the sensor and +/-7.5 deg due to the flight system.	This requirement has not been tested, but in the TWINS instrument the direction accuracy was below 15 deg. With more boards, as is the case of MEDA, the requirement should be achievable.
	MEDA shall characterize the horizontal component of the wind at the sensor location with a resolution of at least 0.5 m/s in speed in the 0-10 m/s range, and of 1 m/s for wind speeds above 10 m/s up to 40 m/s	The speed range resolution has been validated for temperatures below -50 ^∘^C, and up to a resolution of 1.25 m/s for maximum temperatures of +20 ^∘^C

### WS Measuring Concept, Design and Description

The MEDA wind sensor system is a direct evolution from two previous designs flown to Mars: REMS on NASA’s MSL rover mission, and TWINS on NASA’s Insight lander mission. The MEDA WS is based on hot film anemometry, as in the case of REMS and TWINS. The MEDA Wind Sensor (WS) consists of two short, horizontal booms extending from the RSM, separated by $120^{\circ }$ of azimuth, mounted about 1.5 m above the ground. WS1 extends horizontally from the RSM towards azimuth $6^{\circ }$ (rover azimuth $0^{\circ }$ points directly forward), and WS2 is rotated $120^{\circ }$ clockwise from WS1 (see Fig. 29). The placement of WS1 and WS2 on the RSM and their $120^{\circ }$ azimuthal separation are intended to mitigate rover hardware wind interference as much as possible. Nevertheless, the RSM itself will affect wind flow patterns, so data collected simultaneously from both booms must be combined to produce valid wind speed and directional measurements.

**Fig. 27:**
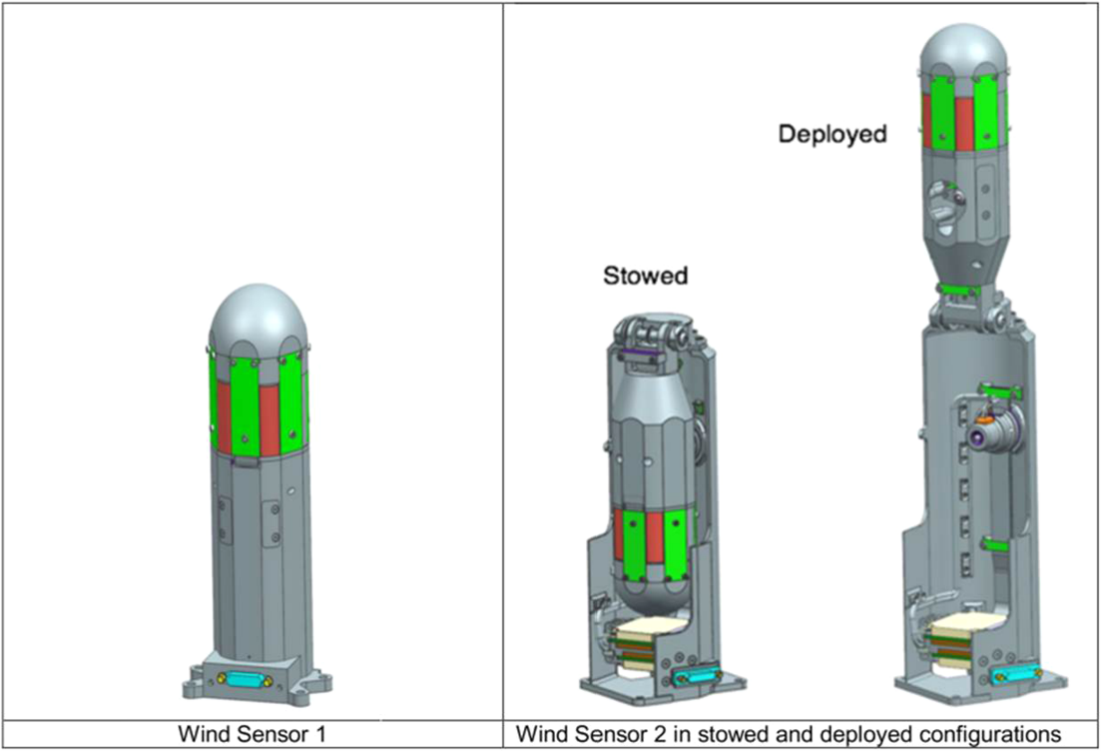
Wind Sensor Design Concept. Green plates are the wind sensor transducer boards

**Fig. 28:**
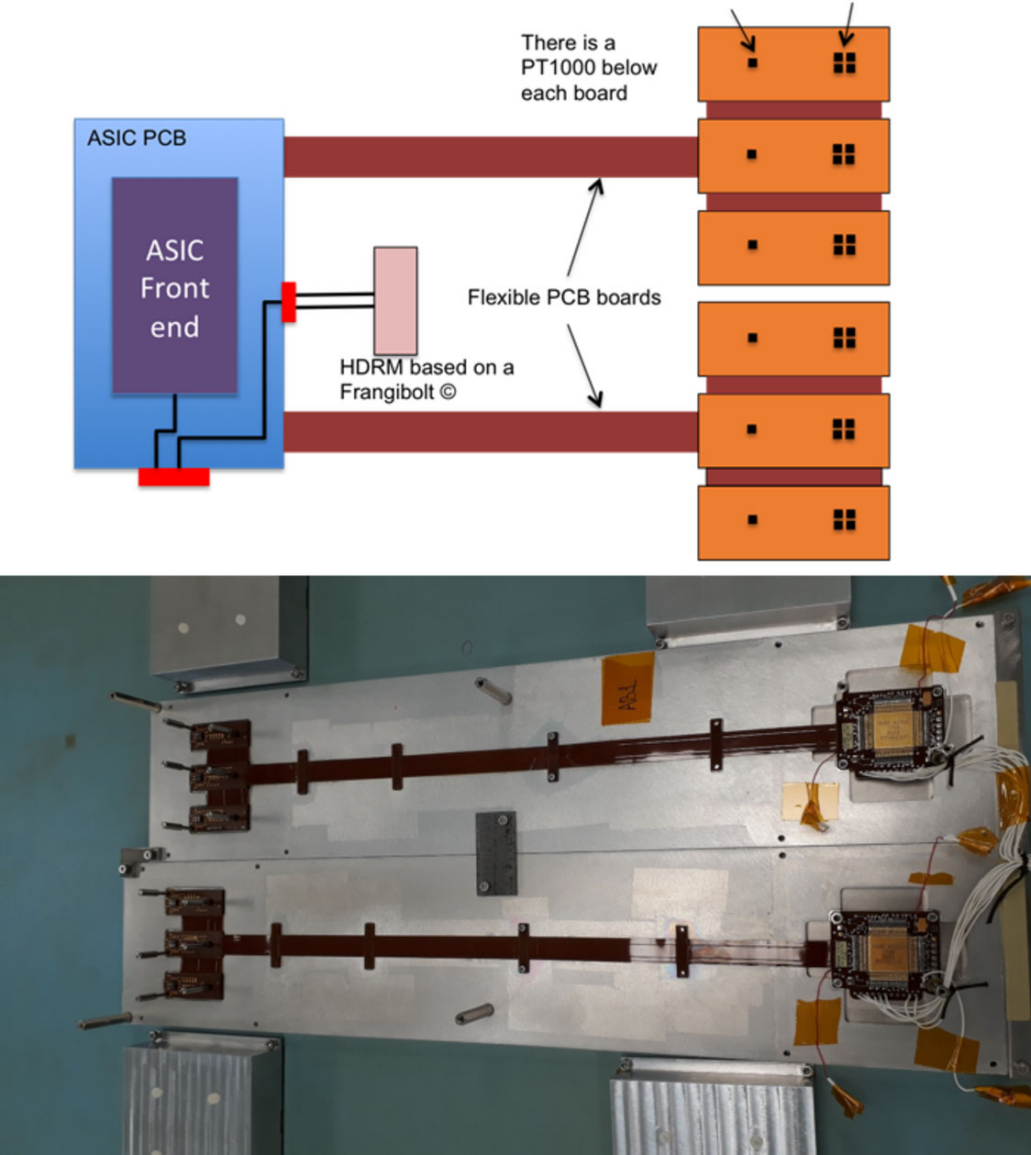
WS2 block diagram. The two transducer boards are connected to the ASIC by flexible PCB The HDRM is controlled directly from the MEDA-ICU by two lines: main and redundant; the main is switched off by a load cell included in the mechanism and redundant by a thermistor also part of the HDRM. The WS1 diagram is quite similar but without the HDRM. Each transducer board includes four hot dice and a single cold dice as well a thermistor in the back that monitors the board temperature. Lower image shows the two WS2 boards during the integration phase (Credit: AIRBUS-CRISA)

**Fig. 29:**
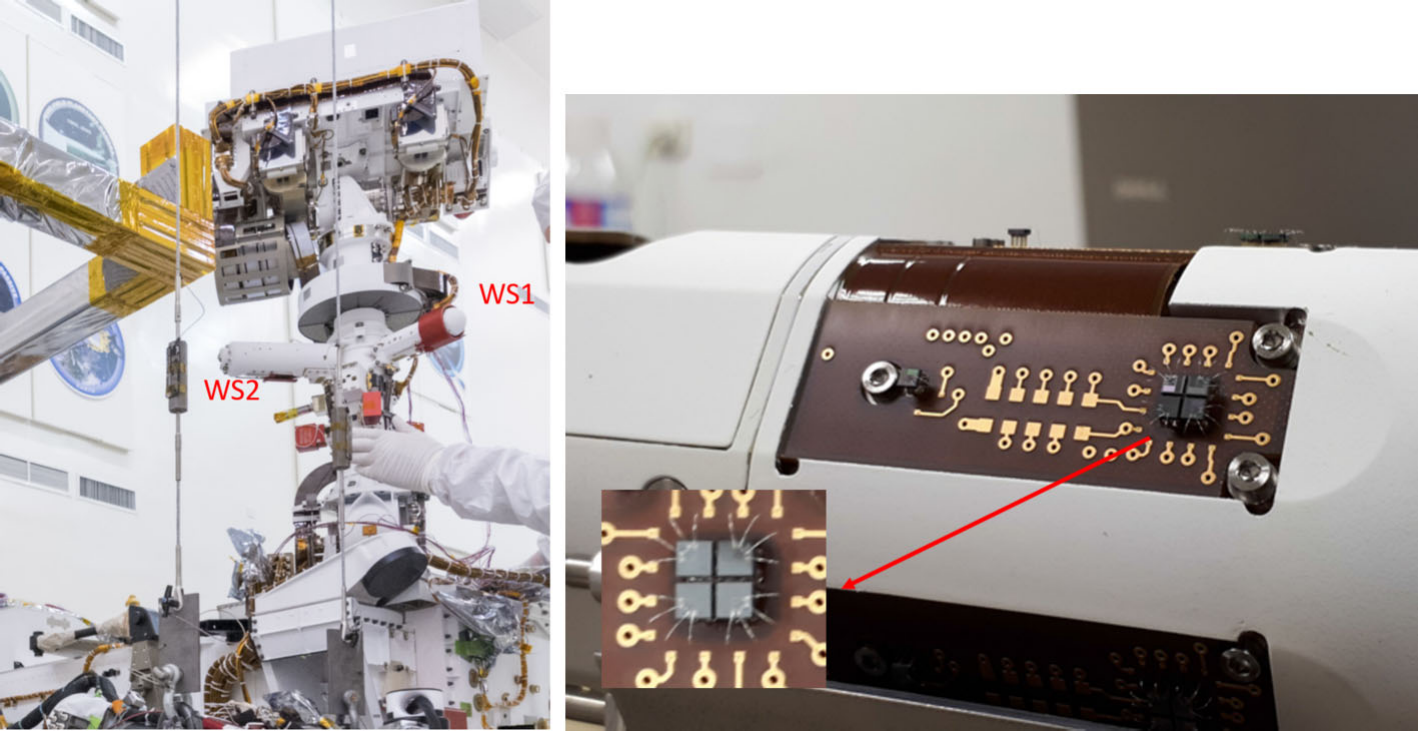
(Left) Position of WS1 and WS2 in their final configuration on the RSM. (Right) WS Transducer Board mounted on breadboard model called MP EQM+. It can be seen the four hot dice and the cold die in the back. Each hot die is a silicon cube which has printed two platinum resistances one for heating and one for sensing wire bonded to the board (see images detail). The cold die is similar but only the sensing part is wire bonded

Each Wind Sensor boom assembly includes 6 wind sensor transducer boards. The novelty of the MEDA WS design is that it has doubled the number of transducer boards per boom in comparison to the TWINS and REMS original design. The increased number of non-horizontal boards (4) will enable MEDA to sense the vertical winds. WS1 is fixed to the RSM, but WS2 is mounted to the RSM as a hinged articulated structure that includes a hold-down and release mechanism (HDRM) that places the deployable boom in its final nominal position, triggered by the MEDA control unit (see Figs. 27-29, left and right, showing the deployed configuration). WS1 is not deployable due to rover volume constraint during cruise

The transducer boards are mounted on two rigid-flex printed circuit boards (PCB): Acquisition Boards, which accommodate the anemometers and their electronics. There are 2 acquisition boards per boom, each hosting three WS transducer boards on one extreme and the ASIC (the Application-Specific Integrated Circuit, in charge of controlling and acquiring the sensor data) on the other. Each wind sensor transducer board is made up of four hot dice and a cold non-heated die, to be used as ambient temperature measurement for the sensor control (as shown in Fig. 28).

Table 21 summarizes the mechanical dimensions of each MEDA WS.

**Table 21:** WS dimensions

	Length	Diameter	Weigth
WS1	170 mm	50 mm	305 gr
WS2	220/393 mm	50 mm	665 gr

### WS Operation and Onboard Data Processing

The ASIC front end uses a closed Sigma-Delta thermal loop to deliver enough power to the hot dice to maintain them at a constant at a constant temperature that is warmer than the ambient atmosphere and is the highest achievable temperature with the available power. The circuit measures the power delivered to the hot die, and as the temperature between it and the ambient atmosphere is also known, the thermal conductance to the CO_2_ ambient atmosphere can be computed. This thermal conductance is related to the wind speed.

The four hot dice assembled in a square configuration (see Fig. 29) will provide four thermal conductances. When properly combined, these four values, can provide a conductance estimation (G) in two orthogonal directions (longitudinal and transverse) in a way that the radiation effects are cancelled out (all dice are very close, circa 1 mm), as well other common effects.

Additionally, each boom includes a front-end mixed ASIC to condition, acquire and process the data from the wind sensor transducers, and to communicate serially with the Instrument Control Unit (ICU).

The ICU hosts the algorithm that interacts with the ASIC, which controls the wind sensor transducers. The ICU control algorithm is composed of three phases, the first of which is dedicated to set the hot dice targeted temperature to the ambient temperature value. The successive phases are intended to increase the hot dice temperature, up to the maximum temperature achievable with the available power. In the same way, when the ambient temperature or wind speed decreases, the algorithm lowers the targeted dice temperature. The control algorithm also implements a series of alarms to supervise communications and both ASIC and dice health.

### Measuring Concept and WS Simulations

As mentioned before, the free-flow wind of the atmospheric boundary layer will be perturbed somewhat by the rover body and the RSM. Therefore the wind speed measured at the sensors will be a combination of atmospheric boundary layer flow and perturbations due to rover hardware from this due to rover hardware. Each WS unit will measure the local wind speed at its respective location on the RSM. In order to do so, the local gas thermal conductance is evaluated at each die based on the power injected (Dominguez et al. 2008), combining the results of the dice, two estimators are obtained for each board (the orthogonal longitudinal and transverse gas thermal conductance estimator $G_{L}$ and $G_{T}$). The combination of the 12 estimators are used together with the calibration data to give the local wind speed and direction for the corresponding WS.

**Fig. 30:**
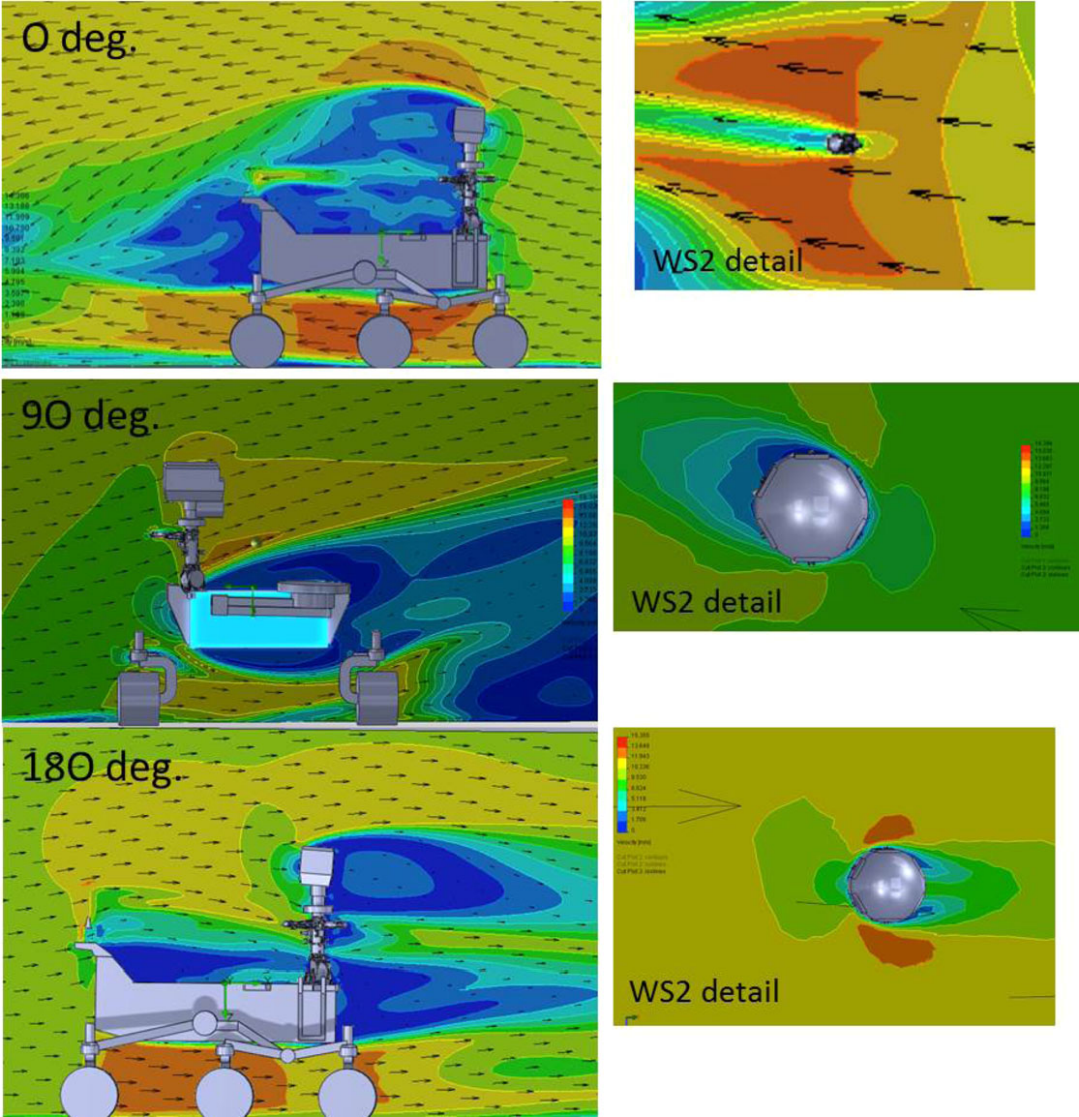
Wind speed field around for several directions ($0^{\circ }$, $90^{\circ }$ and $180^{\circ }$). The simulation has been run with the FloEFD code with a model composed by 2.7 million cells (including solid, partial and fluid cells) (Bardera et al. 2018; Torres et al. 2017)

A computational fluid dynamics (CFD) model has been constructed to evaluate how the free flow wind is affected by rover hardware and to help interpret local wind measurements at each WS location. Figure 30 shows examples based in the wind speed field around the rover for three wind stream incident directions: $0^{\circ }$ (forward), $90^{\circ }$ (lateral) and $180^{\circ }$ (rear). The CFD model to evaluate the rover hardware perturbation has been further analyzed, comparing the results of simulations with wind tunnel tests of a scale model of the rover and WS at equivalent Reynolds numbers to those expected on Mars (Bardera et al. 2018).

### WS Calibration

The calibration of the WS is composed of two set of tests: one for determining specific WS parameters for each particular boom and another set to obtain a data base for correlating wind speed and directions with board estimators (longitudinal and transverse). The latter is the basis of the wind data retrieval.

WS parameters are those required for the computation of the power injected to each die to maintain its operational temperatures, and they are:

1. Determination of the thermal constant of hot dice platinum resistance.
2. Measurement of the ohmic resistance of each hot dice (heating and measurement resistors).
3. Determination of the conductive parameters of the hot dice pillars and bondings.

The test to estimate the thermal constant of the dice platinum resistance is necessary since the value of the resistors and their temperature coefficients is used to compute the power consumption. The thermal constant is determined measuring die resistance (four wire method) at several temperatures, submerging it in a precalibrated ethanol bath. A multiple 2nd degree regression is carried out with the results obtained for two sets of resistors.

The conductive parameters test is related to the dice thermal behavior characterization, and determines the combined conduction constant of the hot dice pillars and bondings. An estimation of each die conductive constant and a general dice radiative factor is obtained, using the results from several tests at different temperatures in a thermal vacuum chamber. Using as inputs the injected power, the hot dice temperature, the chamber temperature and the transducer board temperature, a multiple regression outputs the individual conduction constants and general radiative constant (Kcond and Krad). Figure 32 shows the adjustment of these constants for all testing conditions for a particular die.

The WS Local Calibration Tests are designed to create a database that relates $GL_{ij}$ and $GT_{ij}$ (wind sensor i, board j) with wind speeds and directions. In order to simplify and reduce the calibration tests, the relations are expressed in their dimensionless form, by using the Reynolds (Re) instead of wind speed and Prandtl (Pr) numbers, which are generally defined as follows:

$$Re = V \cdot L_{c} \cdot \frac{\rho }{\mu } $$

where V is the wind speed, $L_{c}$ the characteristic length, $\rho $ the density, and $\mu $ the dynamic viscosity.

$$Pr = C_{p} \cdot \frac{\mu }{\kappa } $$

where $C_{p}$ is the specific heat and $\kappa $ the thermal conductivity.

The WS Local Calibration tests are performed both on Flight Models and on Calibration Models. Due to the limited number of tests performed on the Flight Models as a result of Planetary Protection considerations and limited dimensions on the available facility, only a small matrix of the testing database was carried out. The tested matrix for WS1 covers wind direction ranging from -90 to +90, and a Reynolds number up to 2700 (approximately equivalent to a wind speed of 25 m/s at the highest expected pressure and lowest expected temperature at Jezero crater (8.5 mbar and 185 K)). For WS2 the tested matrix covers wind direction ranging from -45 to +45, and again a Reynolds number up to 2700. An extended testing matrix will be covered with the Calibration Models in a wider facility and without planetary protection limitations.

**Fig. 31:**
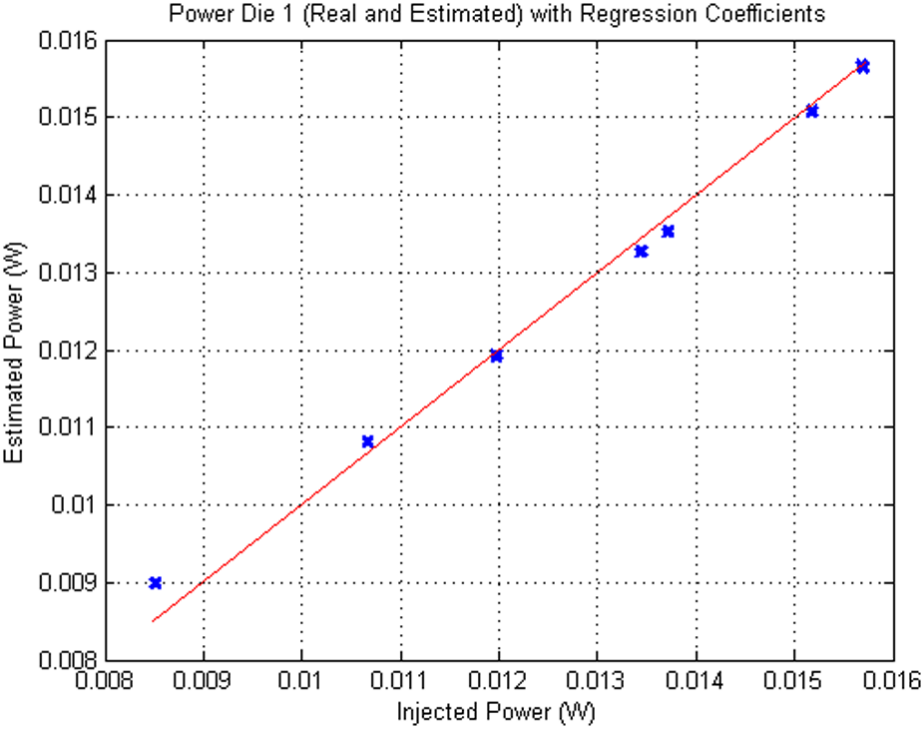
Adjustment of the power loss in the vacuum chamber to the injected power using the multiple regression derived coefficients $K_{cond1}$ (for die 1) and $K_{\mathit{rad}}$

The initial tests were conducted in the Linear Motion Facility at CAB. With this facility, the complete yaw rotation will be achieved (-180^∘^ to +180^∘^), as well as allow to test winds with vertical component. The Linear Motion Facility consists of a 6 m-long pressure chamber, within which a linear track allows wind sensor apparatus mounted on a carriage to be moved at known speeds (Gomez-Elvira et al. 2012). The carriage includes a pan-and-tilt capability to allow the wind sensor to be oriented inside the chamber, allowing the simulation of all possible incident angles. Calibration experiments can be conducted with air or $\text{CO}_{2}$ at different pressures. The sensor response is a function of wind flux Reynolds and Prandtl numbers. The calibration matrix covers a full range of Mars expected Reynolds numbers: This is done running tests combining carriage speed and chamber pressure accordingly. As an example, Fig. 31 shows the WS1 FM1 response of board 2 ($GL_{12}$ and $GT_{12}$) of Transducer Board 1 (Horizontal Board) for different tested yaw angles and a constant Reynolds number.

**Fig. 32:**
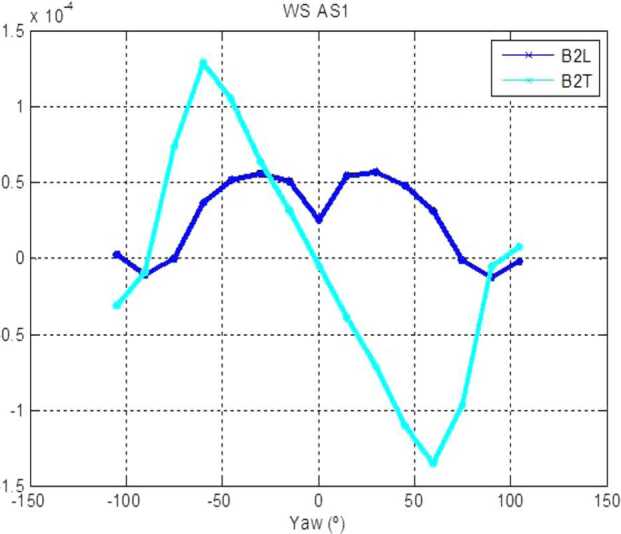
Tunnel tests with WS1 FM1 to obtain reduced set of mesh points. In the vertical axis $GL_{12}$ and $GT_{12}$ (labeled B2L and B2T) and flux direction in degrees in the horizontal axis

## Radiation and Dust Sensor (RDS)

The MEDA Radiation and Dust Sensor, here and after RDS (Rodriguez-Manfredi et al. 2014; Apéstigue 2019) uses a combination of direct sky imaging and, sky-pointing multi-wavelength and azimuthal measurements of diffuse light to capture the diurnal and seasonal evolution of the aerosol optical depth, the aerosol phase function, and the column gas abundance of ozone. Sky imaging is accomplished using an upward-viewing wide-angle camera, RDS-SkyCam, while diffuse light is recorded over different wavelengths and azimuths using two sets of eight photodiodes that are part of the RDS-Discrete Photodetectors or RDS-DP.

Direct imaging of the Sun through solar filters has been used to retrieve aerosol optical depth dating back to the two Viking Landers (Colburn et al. 1989; Pollack et al. 1995). The same technique was used by the Pathfinder Lander (Smith and Lemmon 1999), the Mars Exploration Rovers (Lemmon et al. 2004, 2015), and the Mars Science Laboratory (e.g. Guzewich et al. 2019) to record the seasonal variation of aerosol optical depth at those sites. Those variations have also been estimated even from orbit (Hoekzema et al. 2011; Petrova et al. 2012). However, all of those observations were performed using cameras whose primary purpose was documenting the surrounding terrain and geological context, and the frequency of atmospheric optical depth observations varied and was not always done daily. Imaging of the sky to determine the aerosol particle size and scattering phase function has also been performed by previous landers and rovers (e.g. Tomasko et al. 1999; Soderblom et al. 2008; Smith and Wolf 2013; Chen-Chen et al. 2019) with some limited success in analyzing subdiurnal time scales (Mason et al. 2015; Vicente-Retortillo et al. 2017).

The REMS instrument on-board the Mars Science Laboratory included a set of six upward-viewing photodiodes that spanned the UV wavelengths for the purpose of measuring the radiation environment at the Martian surface (Gomez-Elvira et al. 2012). These photodiodes are also used to estimate aerosol optical depth (Smith et al. 2016) and aerosol particle size (Mason et al. 2015; Vicente-Retortillo et al. 2017) supplementing the observations taken by the Mars Science Laboratory cameras.

The DREAMS payload on board the Schiaparelli lander of the ExoMars 2016 mission, included a radiometer named DREAMS-SIS that presented a “sectored” configuration, in which different detectors with equal characteristics provided simultaneous observations of different areas of the sky, allowing a better time-resolved estimation of the atmospheric optical depth thanks to the simultaneous sampling of the diffuse and direct light contributions (Arruego et al. 2017; Toledo et al. 2017; Esposito et al. 2018).

The strategy established in the design of the RDS was to combine and expand upon all the observations types described above in a single package dedicated to observing the sky and atmospheric properties. The set of photodiodes included in the RDS include more spectral bands than the precedent REMS and DREAMS sensors, from the UV to near-IR, and increase the DREAMS-SIS sectorization by including both zenith-viewing and side-viewing detectors to continuously sample the scattering phase function by observing sky brightness at a wide range of azimuths. These observations require relatively low power and data volume therefore enabling high temporal resolution for long periods of time during each sol. Meanwhile, the RDS-SkyCam improves previous imaging strategies, as it is a fully dedicated camera that will allow imaging of the sky multiple times per sol. Moreover, as it will be shown later, it includes an annular density filter that will allow obtaining sky brightness maps with the Sun in the FoV and no blooming, when scanning of highly over-saturated pixels spreads the saturation signal easily along columns and more slowly across rows, but still a high sensitivity in the poorly illuminated areas of the sky. This combination of observations makes RDS a powerful instrument capable of characterizing dust and cloud aerosols over a variety of timescales and their effect on the ozone chemistry (e.g. Viudez-Moreiras et al. 2020).

### RDS Science Objectives and Requirements

The RDS is designed for the monitoring of Martian dust scattering properties, the detection and characterization of clouds at twilight, and the estimation of ozone column abundance. The brightness of the sky as a function of wavelength and angular distance from the Sun depends on the vertical distribution, the particle size, and the optical properties of aerosols (dust and water ice clouds), as well as on the abundances of gases present in the atmosphere.

**Fig. 33:**
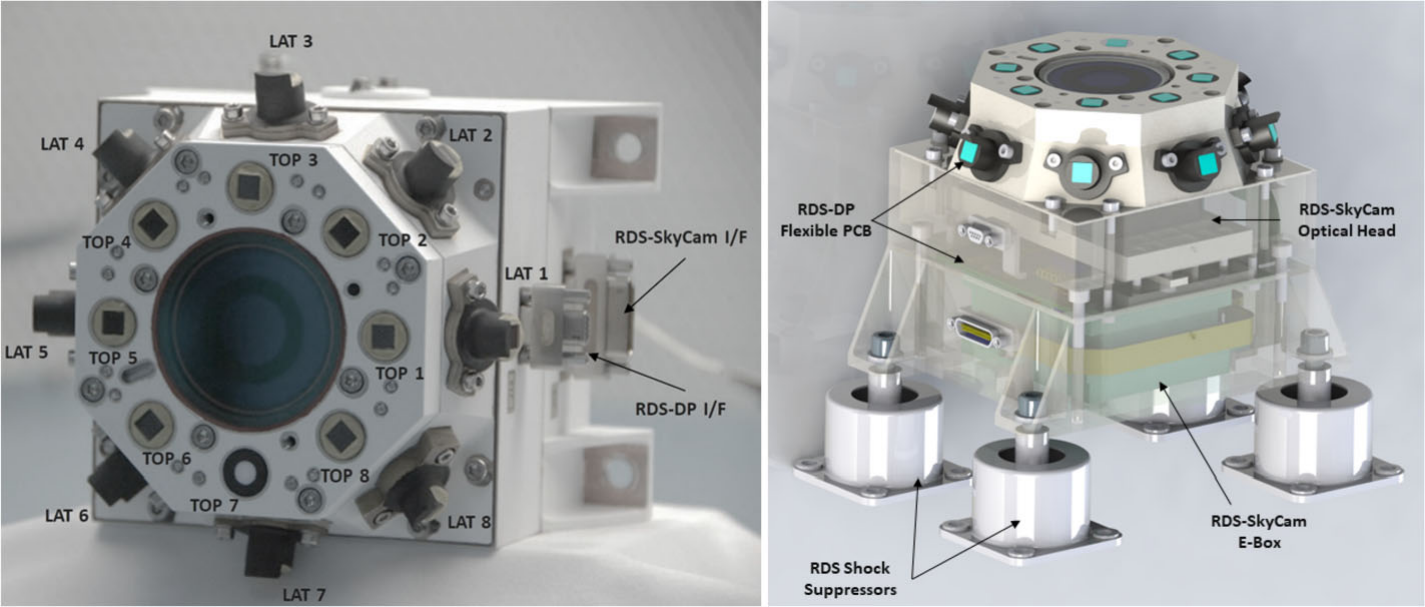
(Left) RDS upper (TOP) and lateral (LAT) detectors disposition, and (Right) RDS assembly 3D view

By combining observations at different sky sectors and wavelengths with radiative-transfer simulations, dust and cloud scattering properties and gas abundances can be characterized and studied (Tomasko et al. 1999; Markiewicz et al. 1999). The RDS-DP radiometer consists of: 7 lateral-viewing photodiodes with the same wavelength filter (750 nm ±10 nm) and orientated at different azimuth angles (here and after LAT detectors, Fig. 33 and Table 23); and 8 top detectors that view the zenith direction with different spectral filters that range from the UV to near-IR wavelengths, (TOP detectors, Fig. 33 and Table 22).

**Table 22:** RDS-DP TOP Channels description

TOP Cannel	Wavelength (nm)	Field of view (^∘^)	Azimuthal position (^∘^)	Elevation^∗^ (^∘^)
1	255 ± 5	±15	N/A	90
2	295 ± 5	±15	N/A	90
3	250 – 400	±15	N/A	90
4	450 ± 40	±15	N/A	90
5	650 ± 25	±15	N/A	90
6	750 ± 5	±15	N/A	90
7	190 – 1100	±90	N/A	90
8	950 ± 50	±15	N/A	90

^*^Pointing angle measured from the horizontal

The science objectives of the RDS-DP are:

1. To retrieve the optical depth, single scattering albedo and phase function of dust particles as a function of season and local time. From these retrievals, we will estimate the particle size and refractive index of dust particles. For this analysis, the RDS-DP lateral photodiodes will be used, along with observations taken by the RDS-DP top photodiodes at different wavelengths to discriminate between cloud and dust aerosols.
2. To determine the optical depth and altitude of water ice clouds at twilight. This information combined with the dust retrievals will enable the study of dust particles as cloud condensation nuclei (CCN). The cloud detection and characterization will be performed by using the color index (CI) (Toledo et al. 2017), defined as the ratio between the Top detectors’ observations in the UV and near-IR.
3. To estimate the column abundance of ozone as a function of season and local time, and to study the influence of clouds on the estimated abundances. Simultaneous observations at the maximum of the Hartley band absorption, i.e. 255 nm, and its end at 295 nm measured by the RDS-DP top photodiodes, will be used to retrieve the abundance of ozone as previously done by Mars’ orbiters (Lane et al. 1973; Lebonnois et al. 1973; Montmessin et al. 2011).
4. To characterize the solar radiation environment at the surface of Mars at UV to near-IR wavelengths, and its seasonal and daily variations. Observations taken by the RDS-DP top photodiodes will enable the evaluation of the radiative forcing produced by clouds and dust.

RDS-SkyCam will provide a 124^∘^ pan-sky view in order to provide instantaneous cross-sky radiance surveys from which physical properties studies may be performed. In addition, SkyCam will include an annular ND-5 coating—when the Sun is within the coated region, direct solar flux measurements may be made. The annular ND coating will provide two times per day (approximately 9:00 and 15:00 local true solar time) when solar flux, and thus aerosol optical depth, may be retrieved.

The combined RDS data set will allow an unprecedented look at sky radiances, and thus aerosol physical properties. SkyCam measures the radiation on the Solar almucantar plane and provides context of the geometric distribution of aerosols. SkyCam will provide imaging across the sky at several times per sol; the sideways looking photodiodes will provide higher precision and accuracy radiance over a subset of scattering angle at many times through the sol and the uplooking photodiodes distinguish between ice and dust aerosols. In addition, Mastcam-Z and SkyCam measurements will be highly complementary. Mastcam-Z can determine optical depth only through use of the rover’s remote sensing mast; however, it may do so at any time the Sun is up. Use of the Mastcam-Z opacity record will also allow tracking of dust on the SkyCam optics: Mastcam-Z can be calibrated via the Beer-Lambert-Bougher extinction law, and simultaneous use with SkyCam can divide the sky dust column into an atmospheric and a contamination component. In addition, Mastcam-Z can be used to provide multispectral, cross-sky radiance surveys to supplement the more frequent, monochromatic, SkyCam data.

SkyCam is designed to provide radiance data in the 600 nm–800 nm visible to near-infrared range, complementing a long record of blue to infrared opacity measurements by previous missions (Colburn et al. 1989; Smith and Lemmon 1999; Lemmon et al. 2004, 2015, 2019). The design incorporates a wide field of view with few obstructions, at least ±60^∘^ from the boresight, in order to achieve pan-sky imaging; a baffle incorporated in the RDS top plate restricts light from outside this range, so that sky images uncontaminated by solar glare may be acquired near sunrise and sunset. The SkyCam response must accommodate typical Martian sky radiances, while simultaneously accommodating solar radiances that are typically 5 orders of magnitude higher. High spatial resolution is not required; one sample per degree would be adequate for model constraints. Note that the camera is capable of 8 pixels/degree, but the thumbnail images can be produced at the lower resolution. In addition, SkyCam may be used for other purposes, such as observing clouds and determining winds from successive cloud images; thus, repeat imaging at a rate of about 1 frame per minute is required.

All these high temporal-resolution observations carried out with by the photodiodes, combined with the camera pictures of the sky taken at specific moments multiple times per sol, will provide a comprehensive characterization of dust and cloud aerosols above the rover site on Mars, characterizing their diurnal variability. The combined suite of observations performed by both RDS will provide key new insights into the seasonal variability of dust and ozone, the influence of dust on clouds, and the water cycle on Mars.

### RDS Design and Description

One of the major constraints during the design of the RDS was the accommodation of the camera. RDS-SkyCam is a residual of the engineering cameras of Mars Science Laboratory (Maki et al. 2012) which in turn was a build-to-print copy of the Mars Exploration Rovers cameras described in (Maki et al. 2003). Its accommodation inside RDS had some important implications: the camera was already manufactured in two boxes linked with a fixed flexible cable without connectors (Fig. 36, right), the thermal design of this device was thought for other mission requirements, the optics had to be redesigned to have a wide-view angle and the capacity to take direct Sun images, the SkyCam cleanliness level requirements were inherited by the interior of the RDS and the camera was not compatible with DHMR (Dry Heat Microbiological Reduction). These aspects, in turn, added important constraints to the RDS integration and design impacting in the final dimension and mass of the sensor.

As the camera hardware was already designed and manufactured, the rest of RDS components had to grow all around SkyCam and its optics. The solution adopted was to use a rigid-flex PCB that allowed to accomplish the high integration levels required (Fig. 33, right). This electronics board (Fig. 35) was divided in two areas linked by a flexible interface, the Optical Head (OH) that incorporates the sensors, the proximity electronics to conditioning the signals as close as possible to avoid noise and the multiplexors needed to send as minimum lines to the Processing Electronic (PE) which includes the ADC (Analog to Digital Converter) acquisition chain, the brain of the sensor based on an FPGA (Field Programmable Gate Array) and a SRAM memory (Static Random Access Memory), the power electronics and the interface with the MEDA Instrument Control Unit (ICU). From the thermal point of view, the electronic box of the camera was thermally decoupled from the rest of the RDS structure using washers to ensure that its 3.5 W heater would be able to warm the unit enough under the worst cold conditions. The test performed in vacuum during the acceptance campaign shown that the internal heater of the camera reached -55 ^∘^C (the lower operational temperature of the camera) in less than 30 minutes, starting from -140^∘^ RDS’ temperature.

**Fig. 34:**
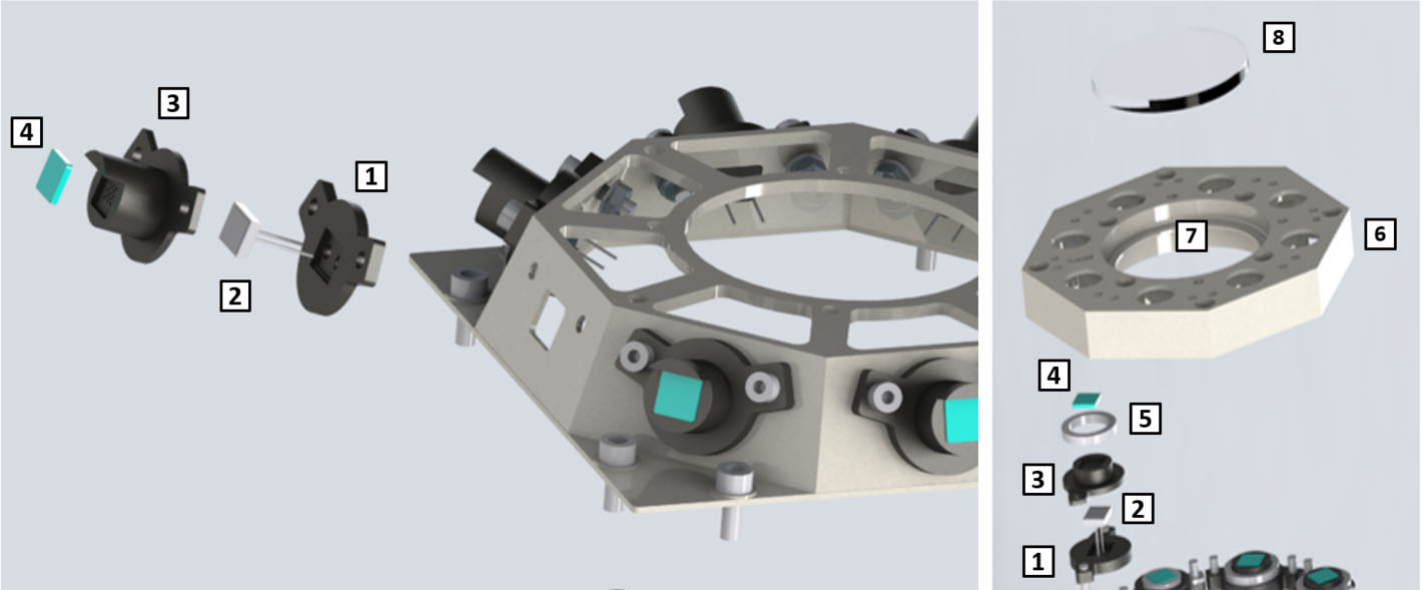
Lateral and Top opto-mechanical sets detail. (Left) RDS-DP Lateral channels (Lat) detail. (Right) RDS-DP Top channels and Top Housing detail

The new optics design entailed a big lens and therefore, less space to integrate the photodetectors and their electronics. The “Top Housing” structure of the RDS (Fig. 34, right-6) was carefully designed to accommodate TOP detectors, and the rest of the components of the optical path of the camera, including a baffle (Fig. 34, right-7) to avoid straight light, and a sapphire window on top (Fig. 34, right-8) to protect the lens from exterior (possible pebbles impacts during landing, atmospheric dust, etc.). The cleanliness levels imposed by the camera implied the use of a laminar flow bench (ISO 5) for the integration of the sensor. The internal surfaces of the RDS were maintained extremely clean, avoiding molecular and particulate contamination on the optics (the structure of the sensor was baked out and regular inspections of particulates were done during all the integration process). Furthermore, camera’s microbiological reduction process incompatibility strongly conditioned the integration process of the sensor. Each part of the structure of the RDS was intensively cleaned and processed by DHMR before their integration, and more than 20 biological assays were done during the integration activities monitoring that the number of internal spores were below the requirement of 300 spores/m^2^. In addition, an HEPA (High Efficiency Particulate Air) filter was included in the venting path of the unit to prevent internal contamination during acceptance test campaign and after, during the mission, to safeguard the environment from a possible internal RDS biological contamination.

#### RDS Discrete Photodetectors Design

The RDS-Discrete Photodetectors (RDS-DP) radiometer design takes heritage from the experience accumulated along the development of a Solar Irradiance Sensor (SIS) for the DREAMS meteorological package on-board of the Schiaparelli descent module of ExoMars 2016 (Arruego et al. 2017).

In the RDS-DP, two sets of 8 photodiodes are used to measure the brightness of the sky as a function of both the wavelength and the azimuthal angle. The first set of 8 photodiodes (TOP channels) points towards the zenith and is described in Table 22. Each channel is made up of a photodiode with an embedded interferential filter (Fig. 34, right-2), a Field of View—FoV mask (Fig. 34, right-3) and a strong magnet (Fig. 34, right-5) to avoid deposition of dust in an external sapphire (Fig. 34, right-4). A Radiation Shield (Fig. 34, right-1) completes the opto-mechanical set at the bottom, to provide 1 mm shielding protection to the interior electronics. This group of upward-looking photodiodes covers a range of UV, visible and near infrared wavelengths suitable for the study of the aerosol particle size and for the discrimination of dust from water ice clouds. The wavelength of the first two channels has been selected in the middle of the Hartley Band (255 nm) and out of its limits (295 nm), to be able to estimate the ozone column abundance by means of the difference absorption that this gas has in those bands. A so-called “total light” detector, meaning that no filter is applied so that its spectral response ranges from 190 to 1100 nm, completes the set, allowing the measurement of the integrated incident radiation from UV to NIR.

The second group of 8 photodetectors, (named LAT channels), are pointed 20^∘^ above the horizon and distributed each 45^∘^ in azimuth (see Table 23). All lateral detectors have the same interference filter, centered in 750 nm, and a narrow FoV (5^∘^). They have been designed to measure simultaneously the brightness of the sky at different directions with the objective to extract information of the dust shape and size. The retrieval strategy is similar to the employed before with previous rover engineering cameras’ observations on Mars (Smith and Lemmon 1999; Lemmon et al. 2004 and 2015; Guzewich et al. 2019). The opto-mechanical set assembly for these channels is mainly the same of TOP ones but without the magnet ring. The avoidance of dust deposition over the external sapphire is done by a sunshade protruded in the FoV mask (Fig. 34, left–3).

**Table 23:** RDS-DP LAT Channels description

LAT Cannel	Wavelength (nm)	Field of view (^∘^)	Azimuthal position (^∘^)	Elevation^*^ (^∘^)
1	750 ± 10	Blind	0	20
2-7	750 ± 10	±5	45, 90, …270	20
8	750 ± 10	±5	315	35

^*^Pointing angle measured from a reference point aligned to the connectors side and anticlockwise

^**^Pointing angle measured from the horizontal

Due to the complicated process of the RDS accommodation into the rover deck, in the early stages of the project, two of the lateral channels were modified with regard to the initial design: (a) Lateral 1 was blocked by the Sampling and Caching Subsystem (SCS) so it was decided to blind it to serve as a reference sensor of the degradation due to radiation. This solution was already used in previous works (Jimenez et al. 2012; Arruego et al. 2017) and it is based on measuring the increment of the dark current of the internal photodiode during the mission. This increment is related to the displacement damage produced on the detector by high energy particles such as protons and heavy ions. (b) Lateral 8 was partially blocked by the same SCS hardware, but in this case the adopted solution was to modify its pointing angle, moving it from 20^∘^ to 35^∘^ elevation, thus avoiding the interference.

**Fig. 35:**
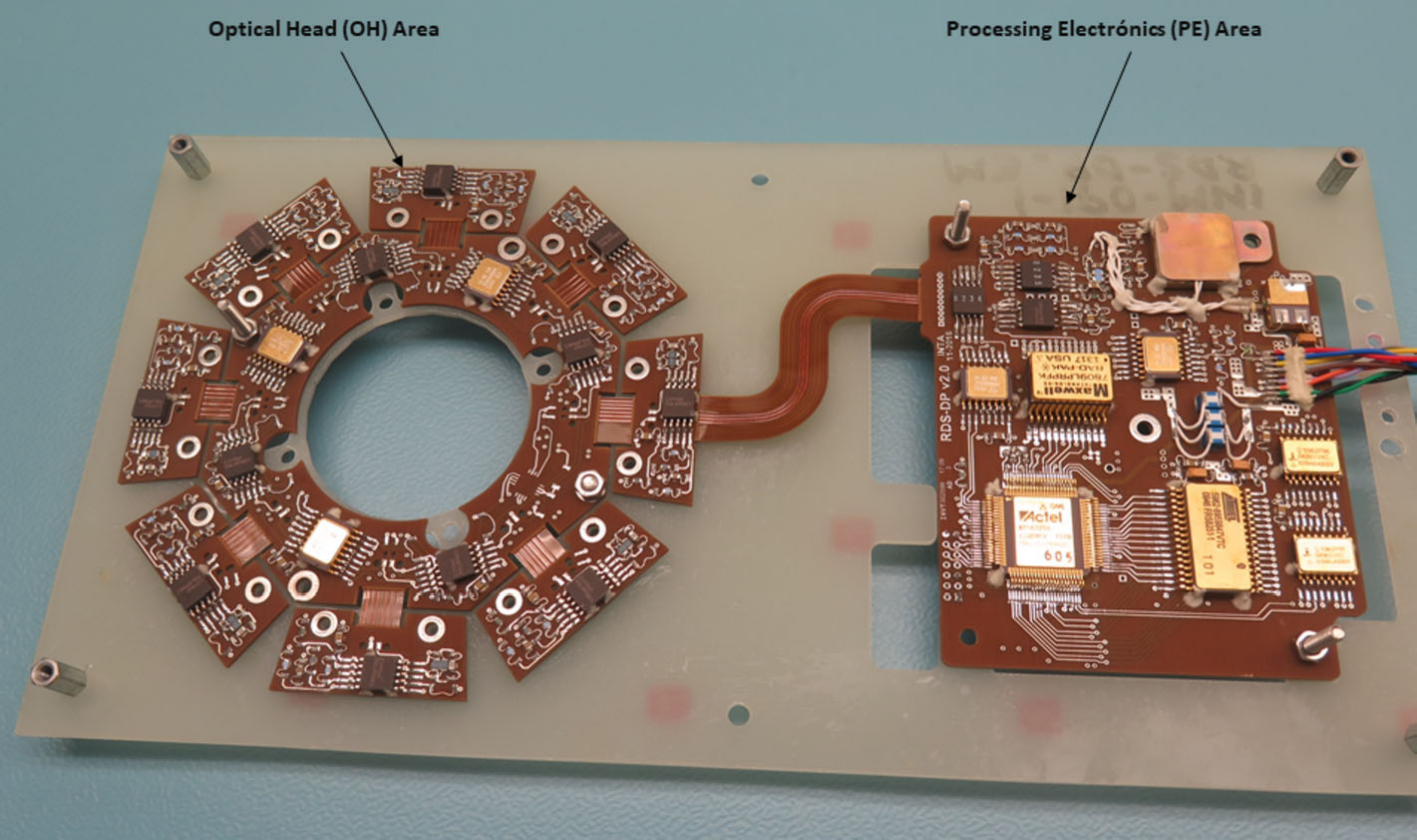
RDS-DP flexi-rigid PCB with two differentiated areas: Optical Head (OH) and Processing Electronics (PE)

The final sensor configuration has evolved during the project from the initial proposal, where the different optical detectors would have been wired directly to MEDA’s Instrument Control Unit (ICU) in order to be acquired, to the final configuration where the RDS-DP becomes a completely digital sensor. The actual RDS-DP has the capacity to condition the signals as close as possible to the detectors and to acquire them through a precise analog to digital conversion. An anti-fuse FPGA (Field Programmable Gate Array) is in charge of capturing the signal of each channel, filtering and storing it into a SRAM memory until the ICU downloads the data (Fig. 35). This upgrade in the design provided a significant increase of performance, in terms of signal quality, an allowed to meet the demanding scientific requirements for UV and lateral channels.

Given that RDS operates directly exposed to the Martian atmosphere, the use of non-thermally electronics was only possible thanks to an intensive thermal cycling qualification campaign performed to all the components, materials and processes that were to be employed in the construction of the sensor. This complex and time-consuming test campaign (Apéstigue et al. 2015), which simulated three times the duration of the M2020 mission, has been carried out during the last five years at INTA facilities.

#### RDS SkyCam Design

MEDA’s SkyCam is based on previous MER and MSL HazCams (Maki et al. 2003, 2012), which are used for rover navigation. The electronics and detectors are inherited from MER and MSL. The optical prescription’s design and performance are also inherited, however significant redesign of the lens barrel, neutral density (ND) filters, and baffles was necessary because the down-looking cameras were not designed to have the sun in the field of view, which causes stray light and internal reflections, ghosting and narcissism, which were superimposed on the detector.

The key similarities with Hazcam are as follows: (1) The camera has the same fisheye optical system to allow a large field of view, 120^∘^ across. (2) The detector is the same: a CCD with 1024×1024 active pixels and 32 columns of virtual pixels. (3) The readout electronics are the same, with 12-bit analog to digital conversion in units of a counter Digital Number (DN). (4) The read out is done via rapid frame transfer to a non-illuminated region and slow readout with no physical shutter.

**Fig. 36:**
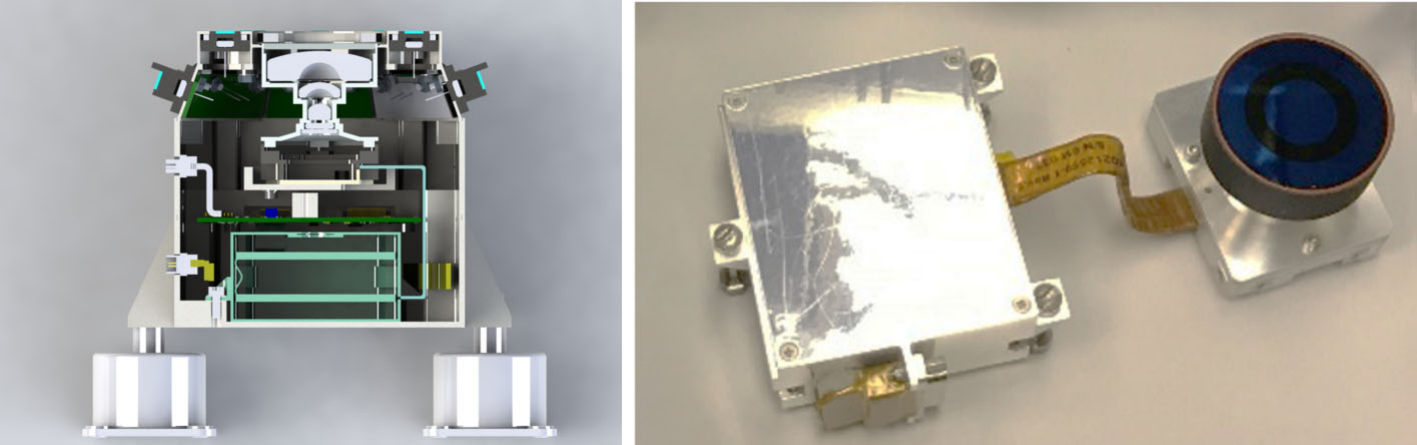
(Left) Cutaway view of the SkyCam within the RDS. (Right) RDS-SkyCam Flight Model unit

**Fig. 37:**
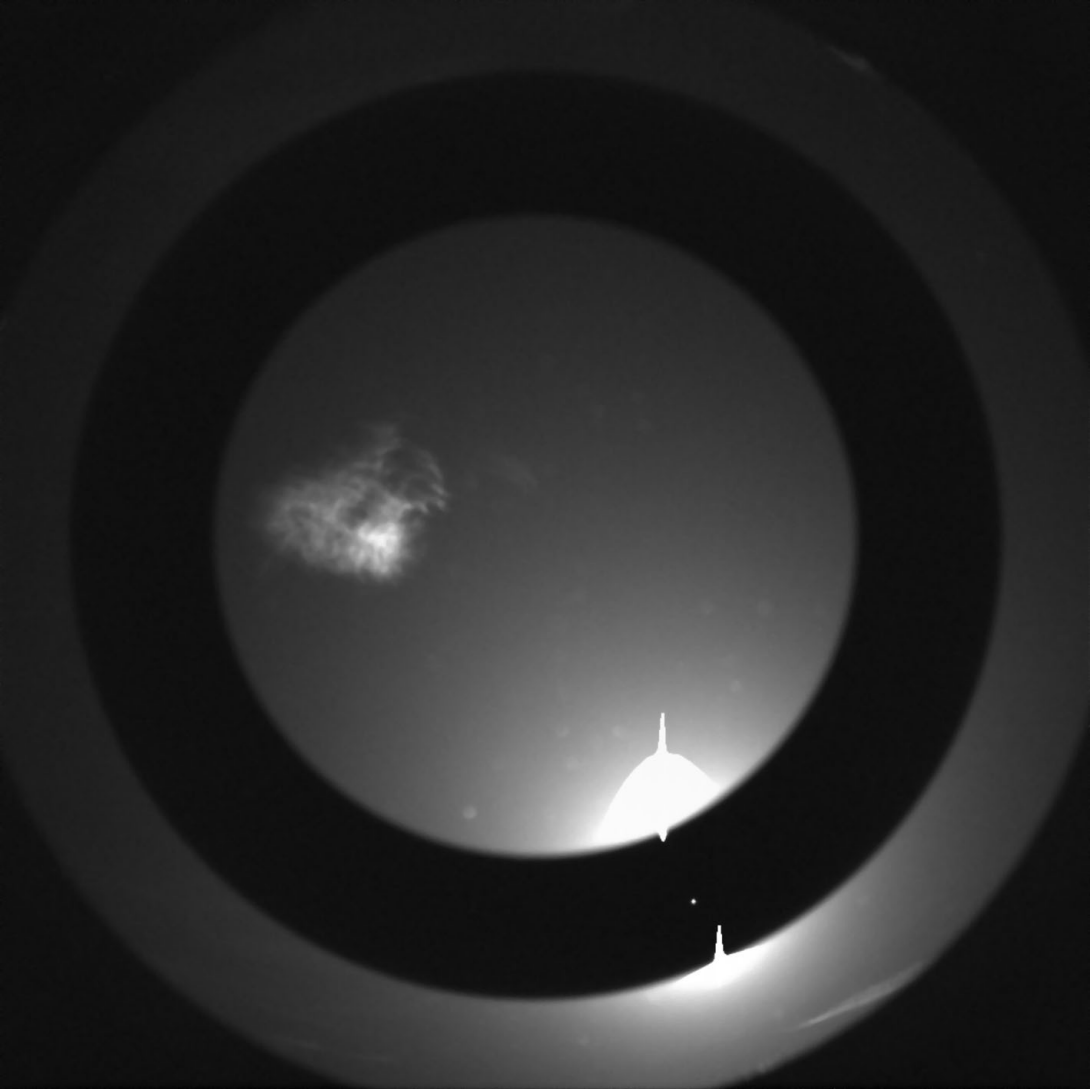
SkyCam QM image of California sky. Clouds appears to the left and lower right; the Sun is visible with the ND-coated annulus. The bright sky near the Sun causes saturation and column bleeding

The key changes from Hazcam are seen in Fig. 36 and are: (1) The camera was designed to be mounted with a vertical boresight. (2) On the window above the optics, an ND coating was added to reduce light by 5 orders of magnitude. This coating was designed such that, for any reasonable tilt and season, the Sun would spend 60 minutes per sol transiting the annulus before and after noon. (3) Circular baffles were added in the SkyCam and in the RDS top plate so that a low Sun, out of the field of view, would not produce stray light that dominates sky light. (4) Reflections were minimized for all internal surfaces via dark coatings and baffles. (5) An internal reflective ND-1.1 filter was removed and instead an absorbing ND-1.1 filter was added above the top fisheye lens. Figure 37 shows a test image with the SkyCam qualifying model outdoors in Pasadena, CA, illustrating the detection of clouds and the ND-coated area.

In addition, the camera control was implemented differently. SkyCam may be commanded via MEDA observation table while the rover compute element (RCE) is not awake; or it may be commanded by the RCE. The RCE performs all image processing, either delayed (OT) or in real time (RCE). Options include shutter subtraction (differencing an image and a 0-exposure image to remove bias and frame transfer effects), auto-exposure (RCE only), removal of reference (non-imaging) columns, thumbnail extraction, and compression.

### RDS Calibration

#### RDS Discrete Photodetectors Calibration

The calibration procedure of the RDS-DP follows the same methodology applied for previous Mars’ radiometers developed by INTA (Jimenez et al. 2018) which is based on the spectroradiometric transfer, from a standard lamp to a detector, in well-controlled laboratory conditions. In these conditions, the calibration model assumes that the wavelength, the temperature and the relative position of the incident light can be considered as independent variables and, therefore, the output signal of every optical channel can be expressed as:

$$I(T,\varphi ,\theta ,E_{\mathit{Sun}}) = \mathit{ARF}(\theta ,\varphi ) \cdot \mathit{TRF}(T) \cdot R_{\lambda _{1_{\mathit{Sun}}}}^{\lambda _{2}} \cdot E_{\lambda _{1_{\mathit{Sun}}}}^{ \lambda _{2}} + \mathit{offset}(T) $$

where $R_{\lambda _{1_{\mathit{Sun}}}}^{\lambda _{2}}$ is the mean throughput between $\lambda _{1}$ and $\lambda _{2}$ of a detector under a solar light spectrum irradiance expressed in Am^2^/W; $E_{\lambda _{1_{\mathit{Sun}}}}^{\lambda _{2}}$ is the Sun irradiance between $\lambda _{1}$ and $\lambda _{2}$ and is expressed in W/m^2^; $\lambda _{1}$ is the initial wavelength of the band pass filter of the channel; $\lambda _{2}$ is the final wavelength of the band pass filter of the channel; $\mathit{ARF}$ is a dimensionless parameter, the Angular Response Function, that represents the channel output dependence on the incoming light angle and takes values between 1 and 0; $\mathit{TRF}$ is another dimensionless parameter, the Thermal Response Function, and represents the thermal dependence of the channel response. It is a correction factor referenced to the highest temperature of the calibration range (57700 counts that represent around 50 ^∘^C); and the $\mathit{offset}$ term, temperature dependent, that represents the sum of every offset produced by the conditioning electronics and by the dark current of the photodiode. As the calibration has been carried-out in an end-to-end basis (i.e., using light as input, and final ADC counts as output), the different parameters have been calculated directly against the number of counts obtained by the ADC, thus avoiding partial conversions to physical magnitudes that are not relevant in the calibration process (for example, $\mathit{TRF}$ is not related to a real temperature, but to the ADC value of the temperature sensor, regardless of the absolute calibration of this one).

The calibration was carried out in the SPASOLAB (Space Solar Cell Testing Laboratory, located at INTA, Madrid). This facility is excellence reference laboratory which is certified and maintained by ESA in order to supplement some technical services provided by ESTEC laboratories. SPASOLAB has several light sources, sun simulators and reference radiometers, which constitute the main part of the calibration OGSE (Optical Ground Support Equipment).

**Fig. 38:**
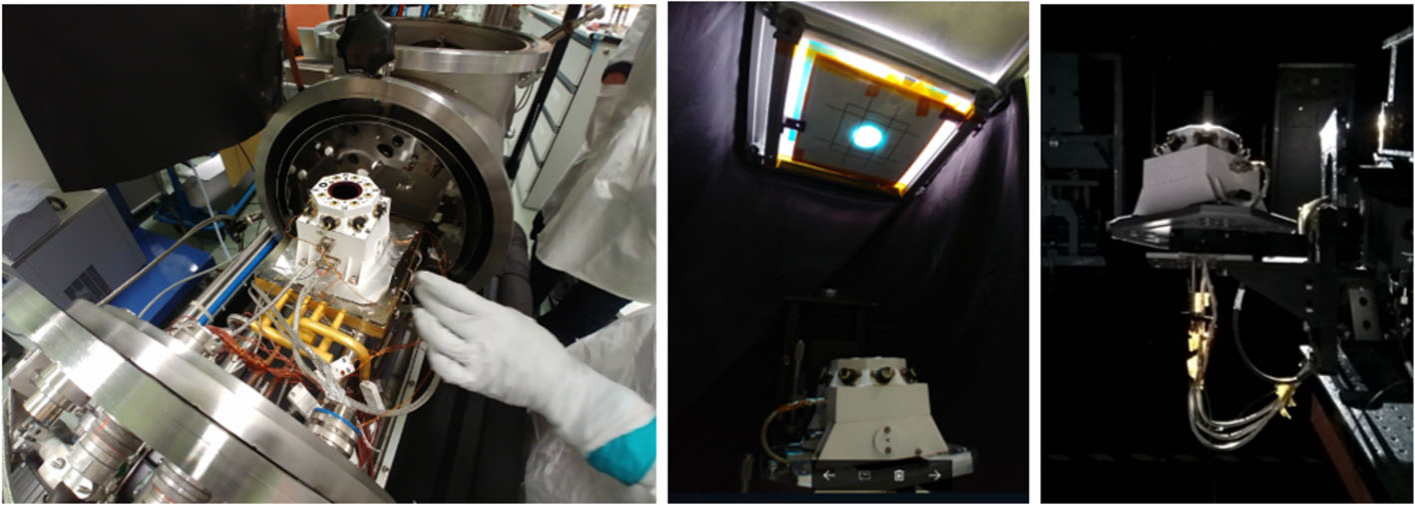
SPASOLAB facilities. (Left) LT/HT Chamber for $\mathit{offset}$ and $\mathit{TFR}$ calibrations. (Center) AM0 solar simulator for irradiance calibration. (Right) 4,5 m far away xenon/mercury lamp for $\mathit{ARF}$ calibration

**Fig. 39:**
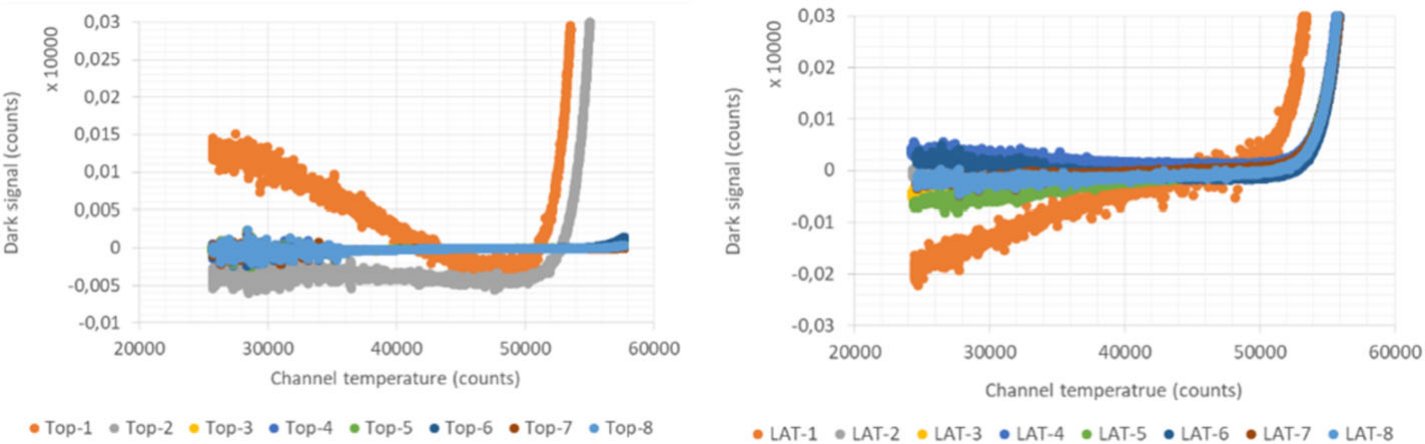
$\mathit{Offset}$ calibration for the TOP (left) and LAT (right) channels as a function of the temperature

**Fig. 40:**
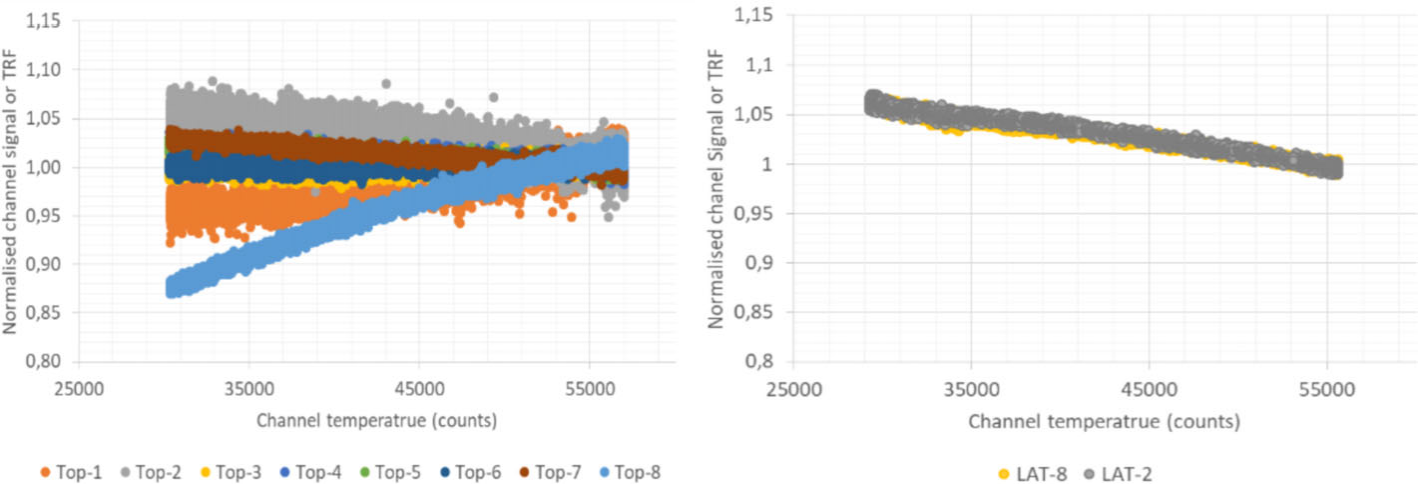
Thermal Response Function ($\mathit{TRF}$) for the TOP (left) and LAT (right) channels as a function of the temperature

The offset and the $\mathit{TRF}$ calibrations use the same setup based on a thermal vacuum chamber (Fig. 38, left) with an optical quartz window (50 cm diameter) which allows the RDS to be subjected to thermal excursions either in darkness, or under a solar simulator which illuminates the sensor with a stable and constant light. As can be seen in Fig. 39, the offset is negligible for TOP channels 3 to 8 (which have low amplifications) for the entire temperature range (-140^∘^C to 40^∘^C). Channels with higher gains, such as the ozone and lateral ones, experience an increase in the dark current for temperatures above 6.45^∘^C (50.000 counts) and 18.19^∘^C (52.000 counts) respectively, which could be representative of low illumination scenarios. Fig. 40 shows the TRF calibration for the TOP (left) and Lat (right) channels that fit well to a second-order polynomial function.

**Fig. 41:**
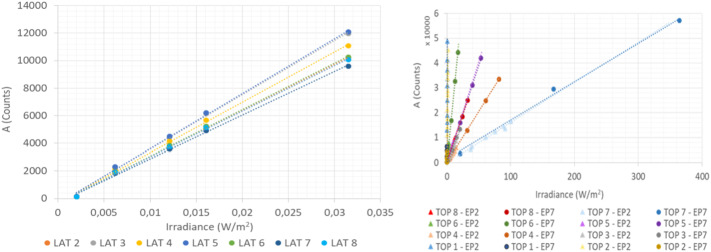
Responsivity Calibration Results for lateral (left) and top (right) channels

The responsivity calibration is obtained by positioning each RDS detector normally to the incident beam of light that comes from the solar simulator (AM0), where the irradiance level and light spectrum that reach the RDS are well known and controlled. To get the points of the curves (Fig. 41), the intensity of the light source is controlled by using different power configurations of the simulator, as well as by interposing individual neutral filters or stacks of them in front of the light beam. For the case of the UVA TOP channels a second simulator was used with higher UV emissivity and the results were quite similar and the differences between them were below the uncertainty of the spectroradiometer used as a pattern cell.

**Fig. 42:**
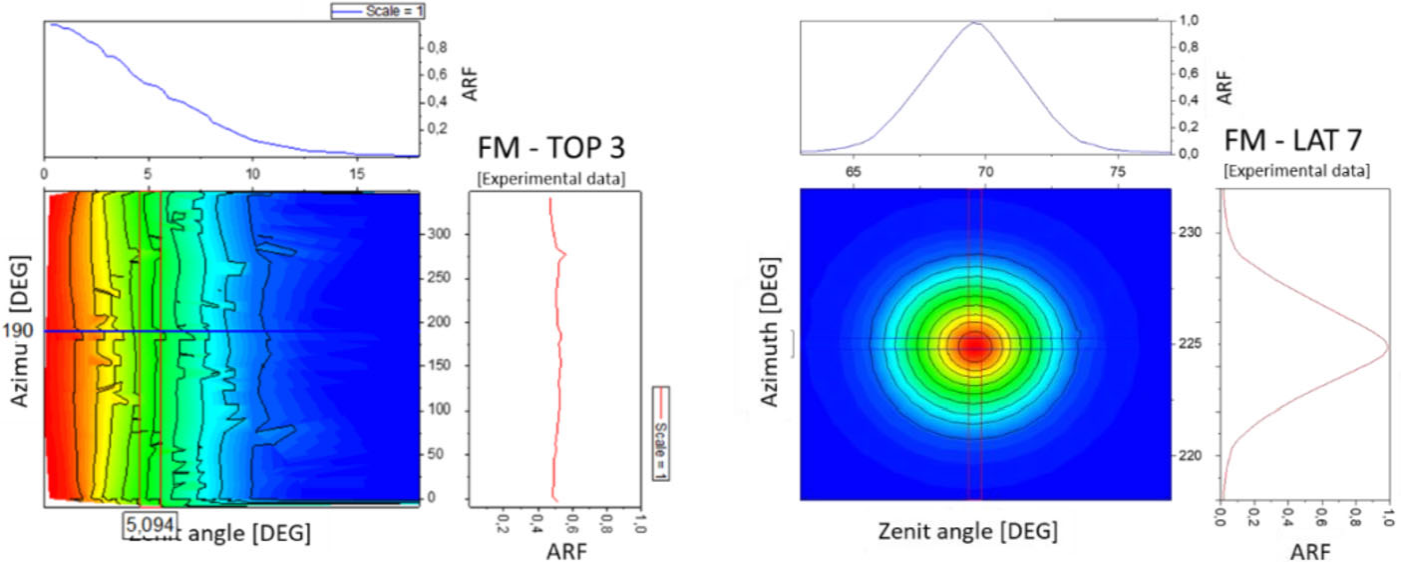
Normalized experimental $\mathit{ARF}$ for a Top channel: TOP3 (left), and a Lateral one: LAT-7 (right)

Finally, the ARF calibration is performed in a dark room facility of SPASOLAB, maintaining a constant ambient temperature and using a stable Xenon lamp situated far enough from the sensor (around 4,5 m) to obtain a uniform plane of light. The RDS is installed then in a robotic 2-axis platform (0,01^∘^ precision each) that allows to situate each detector at any angle with respect to the light beam. A series of sweeps in the zenith and azimuthal coordinates are performed around the normal to each detector while RDS is measuring. An example of the data obtained is shown in Fig. 42, where the values are normalized to the maximum reached in the center of each channel.

The Table 24 summarizes the RDS-DP performance finally accomplished at the beginning of life of the instrument. The accuracy values for Top channels have a strong dependence on the accuracy of the spectroradiometer pattern used in the calibration. However, in case of the Lat channels the accuracy rise up to 6.7$\%$ from 4.5$\%$ (accuracy of the spectroradiometer at 750 nm) where the weight of the angular term $\mathit{ARF}$, increased due to the lateral narrowed FoVs. Precision, understood as the repeatability of the measurements, is really close to the noise for again channels with larger FoV (Top channels), however the influence of the $\mathit{ARF}$ term in the narrowed FoVs is higher as it can be seen for the lateral channels.

**Table 24:** RDS Discrete Photodetectors Channels Performance

Channel	Investigation	Wavelength (nm)	Range^*^ (W/m^2^)	Accuracy (%)	Precision^*^ (W/m^2^)	Noise^**^ (W/m^2^)
TOP 1	Ozone	255 ±5	0.2612	12	6.3⋅10^−5^	3.16⋅10^−5^
TOP 2	Ozone	295 ±5	1.55	5.5	2.25⋅10^−4^	1.14⋅10^−4^
TOP 3	UV flux at surface	250-400	91.25	6.7	5.66⋅10^−3^	2.71⋅10^−3^
TOP 4	Dust and Clouds Properties	450±40	139.5	4.4	8.25⋅10^−3^	4.22⋅10^−3^
TOP 5	Dust and Clouds Properties	650±25	71.85	4.4	4.58⋅10^−3^	2.29⋅10^−3^
TOP 6	Dust and Clouds Properties	750±10	22.095	4.5	1.69⋅10^−3^	8.49⋅10^−4^
TOP 7	Radiative Balance	190–1100	341.5	5.6	2.21⋅10^−2^	1.12⋅10^−2^
TOP 8	Dust and Clouds Properties	950±50	80.85	6.5	6.13⋅10^−3^	3.11⋅10^−3^
LAT 1	Displacement Damage	750±10	N/A	N/A	N/A	N/A
LAT 2	Dust Properties	750±10	0.175	6.7	1.65⋅10^−5^	8.27⋅10^−6^
LAT 3	Dust Properties	750±10	0.148	6.7	1.39⋅10^−5^	6.85⋅10^−6^
LAT 4	Dust Properties	750±10	0.159	6.7	1.63⋅10^−5^	8.10⋅10^−6^
LAT 5	Dust Properties	750±10	0.146	6.7	1.14⋅10^−5^	5.56⋅10^−6^
LAT 6	Dust Properties	750±10	0.173	6.7	2.00⋅10^−5^	1.10⋅10^−6^
LAT 7	Dust Properties	750±10	0.184	6.7	1.92⋅10^−5^	9.63⋅10^−6^
LAT 8	Dust Properties	750±10	0.177	6.7	1.85⋅10^−5^	9.15⋅10^−6^

Once Perseverance rover starts its journey to Mars, RDS-DP detectors and its electronics will suffer the degradation due to radiation environment, mainly by the GCR (Galactic Cosmic Rays) of the deep space. At Mars’ surface, thanks to the shielding that offers the planet and the atmosphere, the degradation will continue but at less of half rate (Hassler et al. 2014). To characterize this effect, a blind detector (Lat 1) has been included in the RDS. It is known that the dark current of a photodetector increases its value due to the displacement damage occasioned by charged particles. Therefore, having this information, it will be possible to estimate the radiation damage and to apply a correction in the rest of RDS-DP channel’s measurements.

However, radiation will be a minor degradation factor compared to the Martian dust when the sensor works on the red planet’s surface. In one hand, previous missions have shown that the ionizing radiation environment is not as hard as predicted by the worst cases models (Zeitlin et al. 2013; Hassler et al. 2014; Semkova et al. 2018). On the other hand, dust will affect the RDS attenuating detectors’ signal along the Martian year as has been seen in REMS UV sensor or in the MERS solar panels. The magnets of the Top channels and the sunshade of the Lat channels will partially mitigate this problem, and of course the strong seasonal winds and the occasional “dust devils”. However, a good estimation of this issue should be done. One possibility is to use the rover cameras that have similar filters that RDS Top channels, and correlate the irradiance measured and then estimate the dust attenuation. Another strategy is to situate the rover with some inclination to force the sun to cross the FoV of the Top channels during one clear day. This will permit to have diffuse and direct contribution of light for each channel at different moments of a sol. Then, using the RDS radiative transfer model, it will be possible to estimate the overall degradation of each channel (as proposer for Dreams SIS instrument in Toledo et al. (2017)).

#### RDS SkyCam Calibration

SkyCam was calibrated in several stages. The camera alone, without the RDS assembly, was tested at JPL in several component-level, room-temperature tests. It was tested at INTA as part of the RDS assembly in subsystem-level tests at room temperature (e.g., stray light) and over temperature (e.g., dark current). It was tested again at JPL in flight configuration as part of rover testing.

##### CCD Characterization

The CCD has a $1024\times1024$ active area, with 17 reference columns (read put columns not attached to physical pixels, diagnostic of electronics performance) read out before the images and 14 after, with a final column containing the camera serial number. The frame transfer time is 5.2 ms, during which the image is moved to a shielded part of the $1024\times 2048$ physical device; readout from there takes 5 s, with no further image acquisition, although dark current is generated. The camera is linear to <1% for 300 DN<signal<3000 DN, and <2 DN for signal<3000 DN. Read noise is 30 electrons, with a gain of 55 electrons/DN. Saturation occurs at 3100 DN, or ∼160,000 electrons.

##### Bias and Dark Current Characterization

The signal from the camera, as read out, comprises three parts: (1) Electronics bias sets a minimum DN level that may be controlled by the commanded video offset (in practice, values other than the default of 4095 are not expected to be used). (2) Dark current accumulates during the exposure and during the readout. (3) The image accumulates during the exposure, and also during the frame transfer process. Each of these components except the image accumulation must be removed either in ground calibration or through the subtraction of a contemporaneous 0-sec exposure image (which removes bias, readout dark current, and the image frame transfer effect, but not the active accumulation of dark current).

**Fig. 43:**
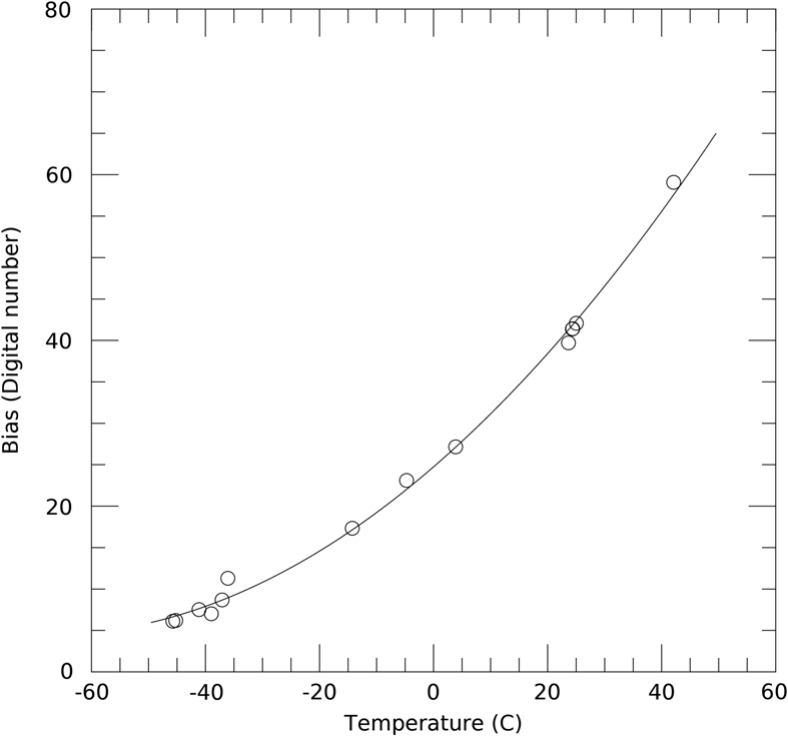
SkyCam electronics bias over temperature, with quadratic fit

The bias is modeled as a polynomial function of temperature (Fig. 43). The mean bias in the first 17 columns, in DN, is $24.52 + 0.595 * T + 0.004505 * T^{2}$, where the temperature is given in ^∘^C. The variation of bias with row is similar to that shown by Bell et al. (2003), Fig. 14b.

**Fig. 44:**
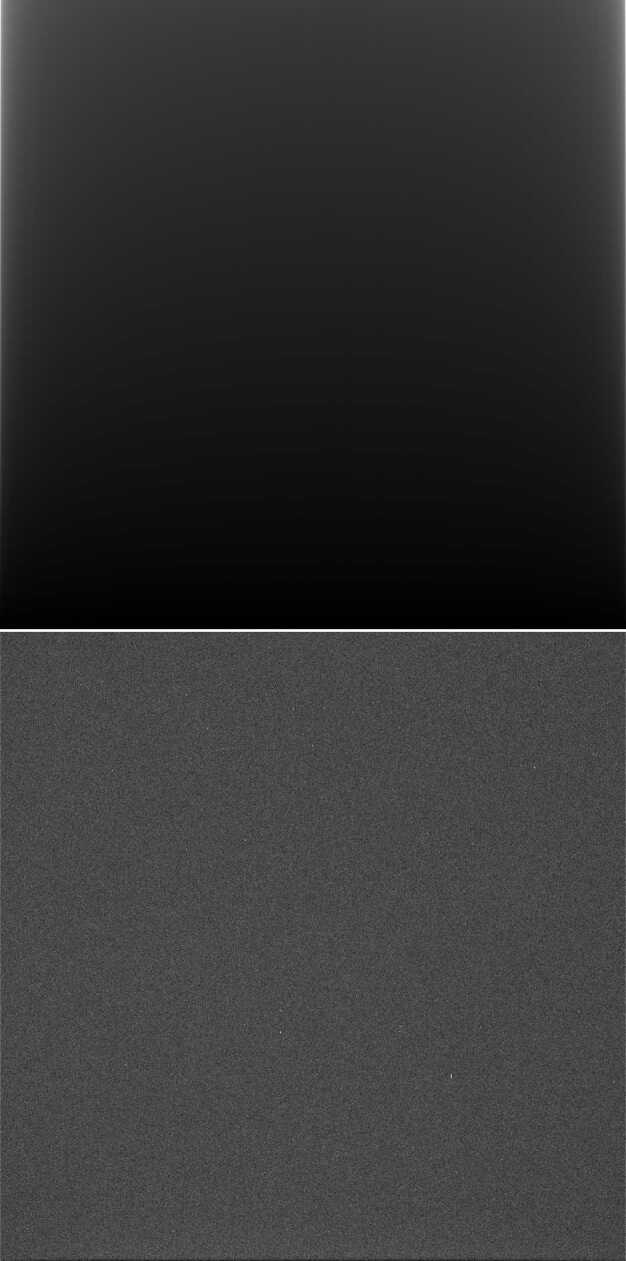
SkyCam dark current field: the frame transfer dark near 30 ^∘^C is shown on the left, the active dark is shown on the right

**Fig. 45:**
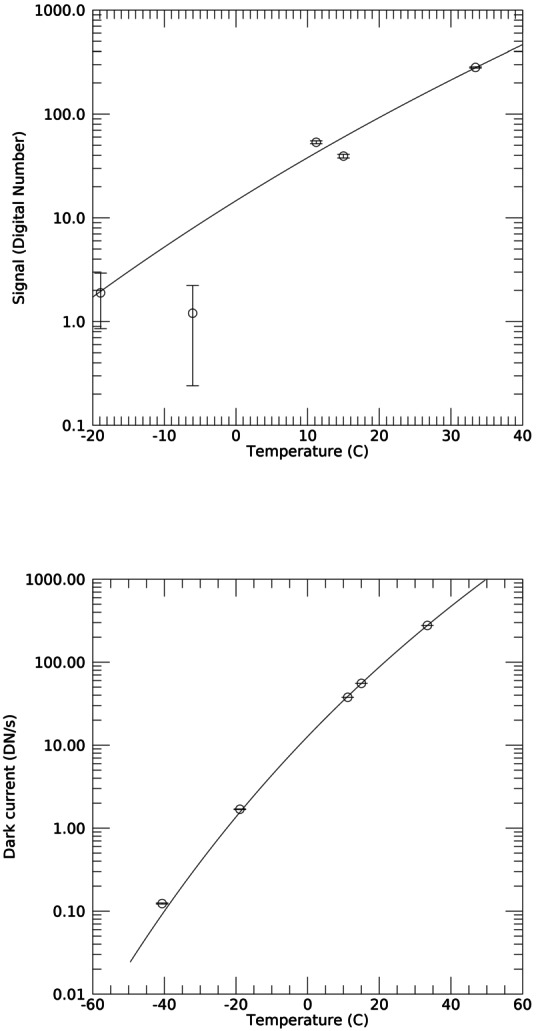
Dark current is shown over temperature. Frame-transfer dark current, averaged for the last two rows to be read out, is shown in DN per exposure. Frame-averaged active dark current is shown as DN/sec. In each case, and Arrhenius fit is shown

Frame transfer and active dark current images are shown in Fig. 44). Over temperature, both follow an Arrhenius law as shown in Fig. 45). The top (last to read out) two rows in the frame transfer dark have mean signal of S = (6.471 DN) * exp(34.610 * X),where X=(273.15 K)/T-1, and T is the temperature in K. The mean value of the active dark is (12.732 DN/s) exp(2.260* X). At the warmest temperatures, fewer than 100 pixels had at least twice the mean dark current.

Operationally, most SkyCam images will be taken with subtraction of a contemporaneous 0-s image. Thus, the bias and frame transfer effect will be removed, leaving only the active dark to calibrate out. In some cases, this may not happen, and the information here will be used to put the various images on the same scale.

##### System Spectral Throughput

**Fig. 46:**
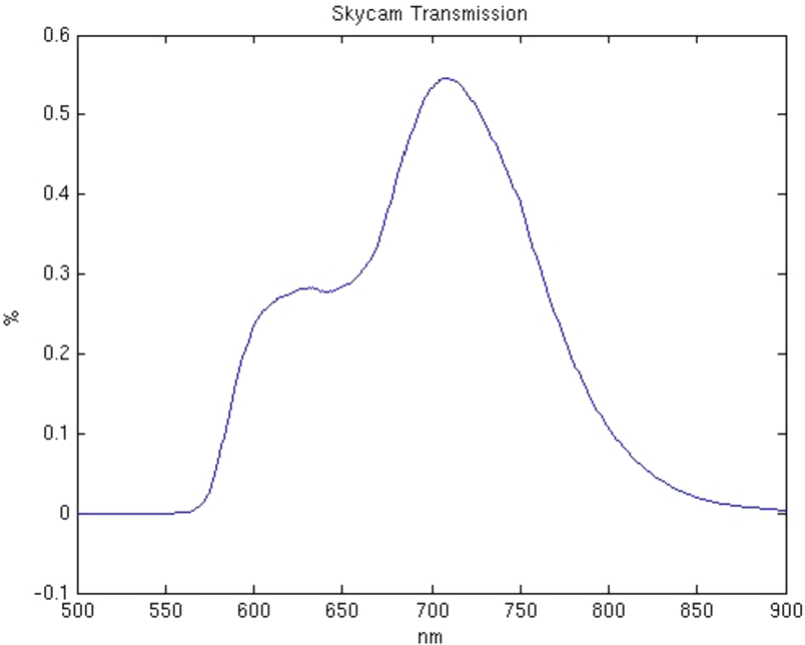
Transmission of the SkyCam optics over wavelength

The system spectral throughput is similar to the Hazcams from which it was derived, and is shown in Fig. 46. The bandpass is approximately 594-777 nm, with an effective wavelength of 699 nm. Including the spectrum of sunlight, the effective wavelength for aerosol studies is 691 nm.

##### Flatfield (Pixel to Pixel Responsivity) Characterization

**Fig. 47:**
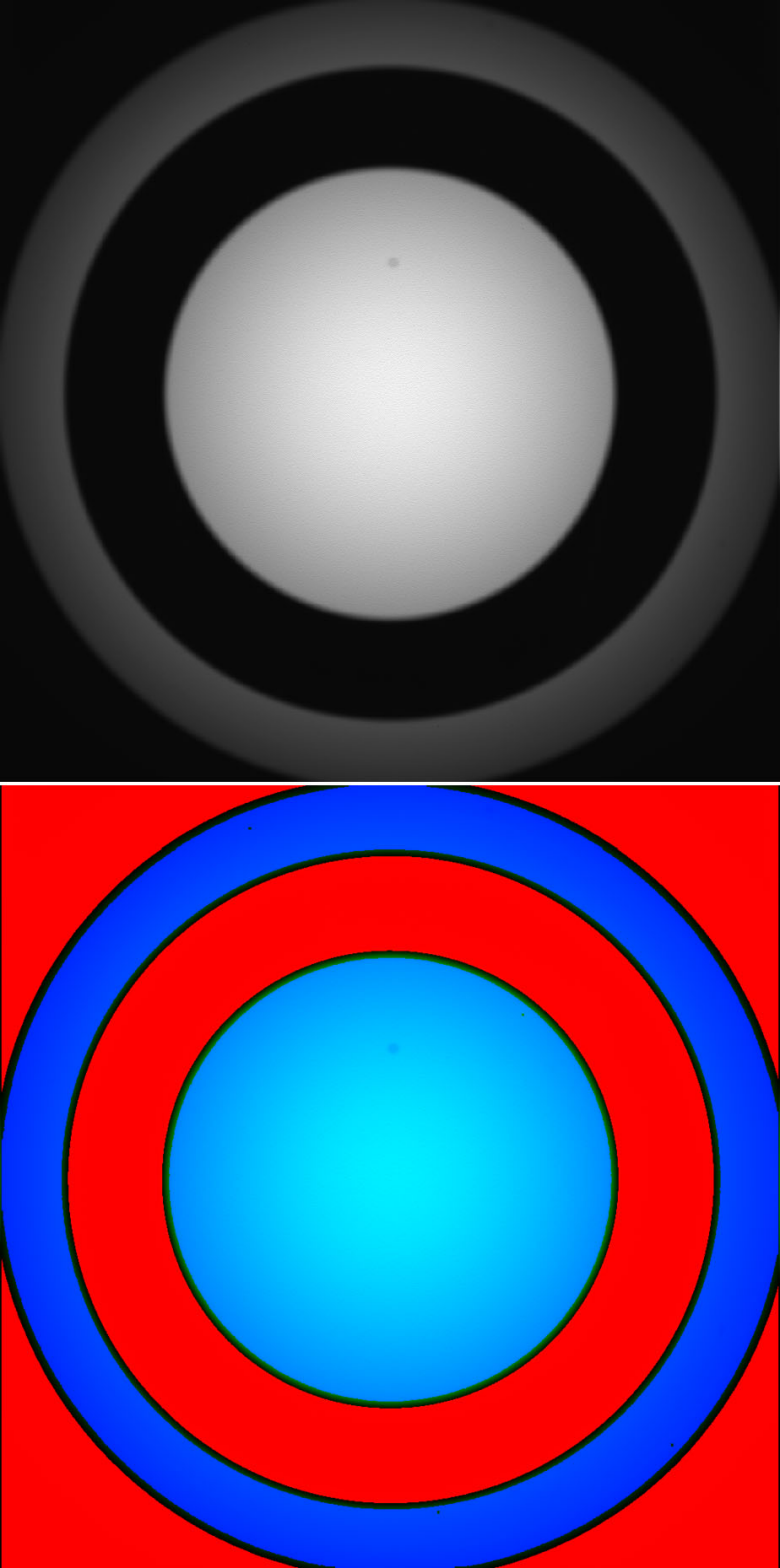
The SkyCam flat field (left). A series of masks (right) identify the areas sensitive to the sky blue), the ND area (red annulus), the baffled area (4 red corners); transition areas are uncolored

The pixel-to-pixel response variation for SkyCam is complex (Fig. 47). First, the fish-eye optics produce an approximately cosine-squared fall-off of sensitivity away from the boresight, with a reduction to 20% at the extreme of the field of view. Second, the baffle produces a circular obscuration that leaves no sensitivity to light in the corners of the FoV. Third, the ND coating reduces sensitivity by 5 orders of magnitude in an annulus. That reduction in sensitivity was not measurable in images of a flat-field source: a signal bright enough to see in the annulus would have produced extensive bleeding from the center and edges, resulting in a non-image. The sensitivity within the annulus was spot-checked with a solar simulator, and the reduction was determined to be 5.00 orders of magnitude. Including the radiometric fall off, the middle of the annulus is 5.4 orders of magnitude less sensitive then the center of the FoV. Fourth, there are pixel to pixel variations at typically sub-percent levels. There are three areas of several percent reduced sensitivity as a result of the ND coating process.

The flat-field measurement was done in parts. Initially, an image of an integrating sphere was used as an approximate flat field. Due to both the fish-eye, and the fact that the integrating sphere was in focus, that left artifacts in the field. A second approximation included a diffuser in front of the camera, with the integrating sphere. That reduced the small-scale artifacts, but introduced an angular dependence. A set of images was taken from a pan-tilt platform, looking at a radiometrically calibrated integrating sphere much smaller than the SkyCam FoV. This set of measurements produced the radiometric calibration (next section), and also allowed for an angular correction to the flat-field image.

In practice, all parts of the FoV will be used. The inner circle and outer annulus of sky light is used for radiance observations of the sky. The ND annulus is used for solar flux measurements. The baffled region, as well as the area of the annulus away from the Sun, is used both as a dark current witness (when the detector is warm enough that this is relevant, and to assess internally scattered (“stray”) light.

##### Radiometric Responsivity

The SkyCam radiometric responsivity was determined in two steps. First, images of a calibrated integrating sphere were taken at room temperature at JPL. Second, images of a constant source were taken through a thermal-vacuum chamber window at INTA as the SkyCam temperature was varied. The response of the central $100\times 100$ pixels was determined to be 3.789E-5 $\mbox{W}\,\mbox{m}^{-2}\,\text{sr}^{-1}\,\text{nm}^{-1}$ / (DN/s) at 23 ^∘^C. No temperature variation was detected: a best-fit increase of 1% over -50 ^∘^C to 40 ^∘^C was observed, which was slightly smaller than the uncertainty.

For a radiance of 0.8 $\mbox{W}\, \mbox{m}^{-2}\, \text{sr}^{-1} \,\text{nm}^{-1}$ at the center, a 140-ms exposures would achieve a signal near 3000 DN and a signal-to-noise ratio (SNR) of ≫100 in each pixel. For 0.005 $\mbox{W}\,\mbox{m}^{-2}\, \text{sr}^{-1}\, \text{nm}^{-1}$ at the center, a 3-s exposure would yield a signal of 400 DN, and SNR of 100 after dark subtraction. A more stressing science case is when the Sun is low and surrounded by a bright aureole, while the sky opposite the Sun at low elevation is dark. In a representative case, a 750-ms exposure would yield 3200 DN for 0.8 $\mbox{W}\, \mbox{m}^{-2}\,\text{sr}^{-1}\,\text{nm}^{-1}$ and 20 DN for 0.005 $\mbox{W}\,\mbox{m}^{-2}\,\text{sr}^{-1}\,\text{nm}^{-1}$; this would result in SNR of 14 for a single pixel in the dark area, but >100 for a 1-degree average (8x8 pixels) and 57 for a $4\times4$ pixel average. The measurement precision (1 DN/pixel) in this case would be 0.0003 $\mbox{W}\, \mbox{m}^{-2}\, \text{sr}^{-1} \,\text{nm}^{-1}$, which is an order of magnitude below the expected noise.

The accuracy is estimated to be <10%. Include something related to the laser test showing no stray light. The relative uncertainty budget, comparing 1 pixel of a typical image to another pixel of another image, is:

1. Measurement uncertainty of 0.25% based on SNR after dark subtraction;
2. Temperature correction uncertainty <1%;
3. Linearity, 0.1%;
4. Flat field knowledge, <1% for central FoV, <2% for exterior annulus.

The absolute uncertainty budget is:

1. Uncertainty in the light source, 2%;
2. Uncertainty in color temperature of the light source, convolved with the camera bandpass, 2.75%;

The absolute uncertainty is thus 4.75% (added linearly, not quadratically, as they are not uncorrelated). The worst case uncertainty is 9.2% (adding all source), while the expected uncertainty is <6% due to uncorrelated sources.

##### Geometric Performance

**Fig. 48:**
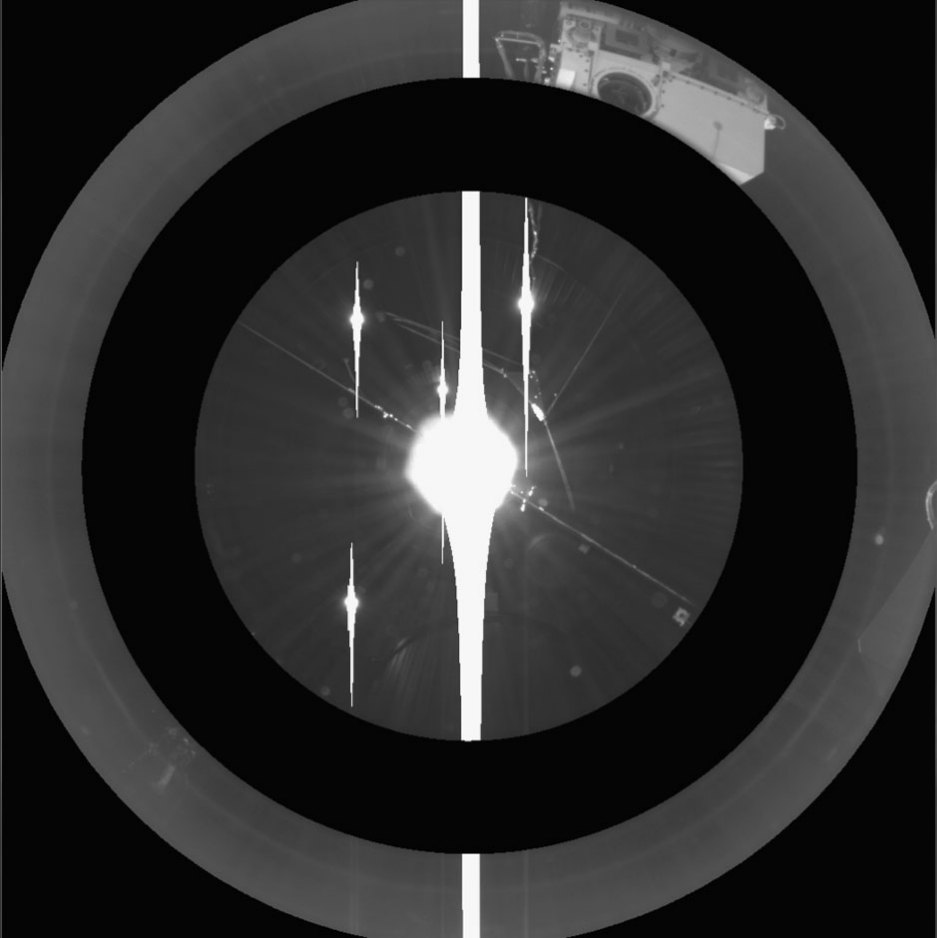
SkyCam image from rover testing within enclosed chamber. The vertical bright streak is bleeding of highly saturated pixels. The remote sensing mast instrument are visible near the top

During the absolute calibration procedure, the angle from the camera boresight to the calibrated integrating sphere was varied in 5-degree steps, row-wise and column-wise, from the center. Based on these measurements, the FoV was determined to be $124.7^{\circ }\pm 0.1^{\circ }$. The resulting instantaneous FoV is 8.28 ± 0.1 pixels per degree; thus, $8\times 8$ binning results in approximately 1-degree sampling. The diameter of the area interior to the baffle is 127^∘^. Tests on the rover show that the field of view is largely unobstructed (Fig. 48), although the remote sensing mast and high gain antenna move.

**Fig. 49:**
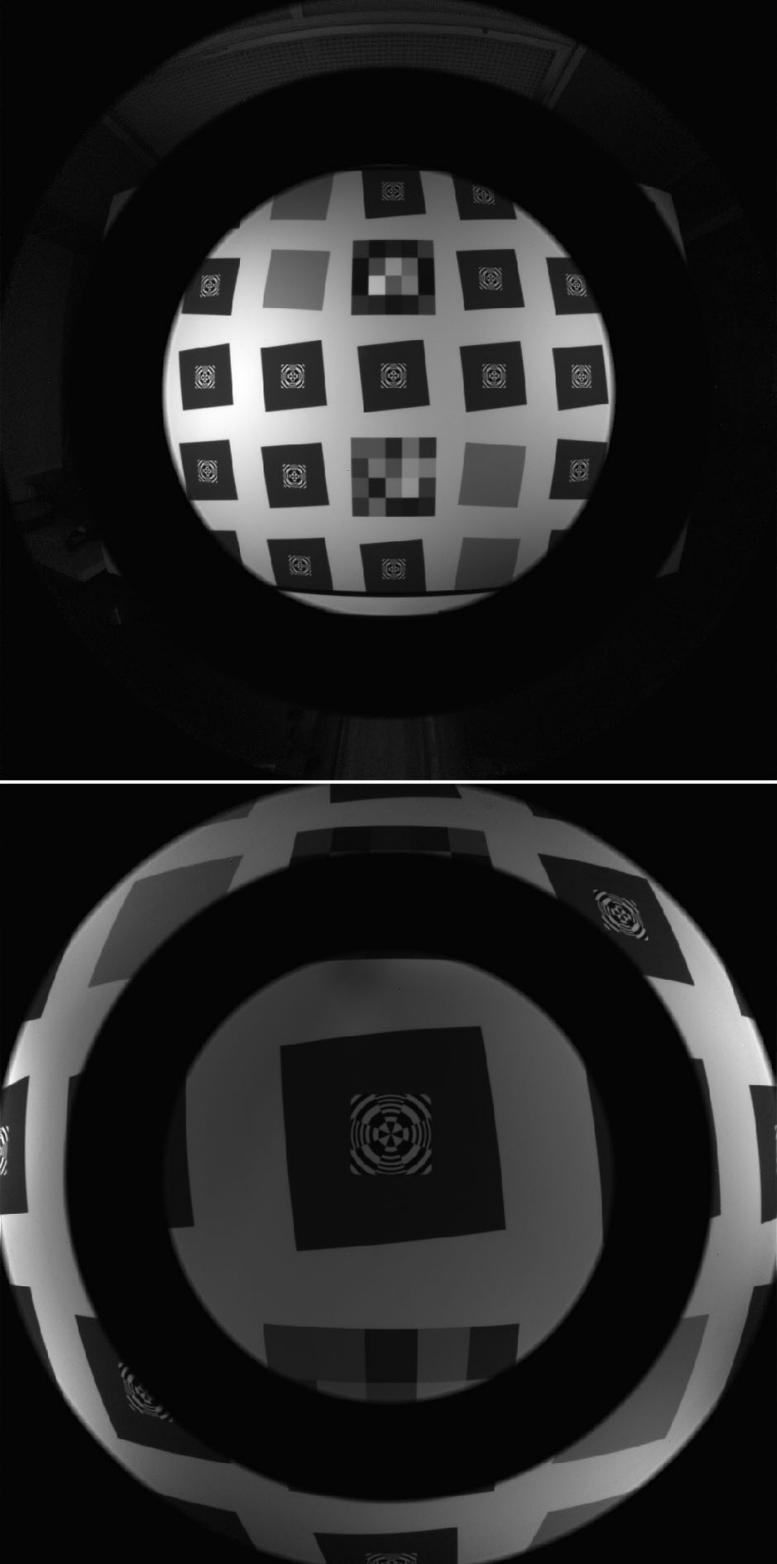
Images of resolution targets were used to assess modulation transfer function

Resolution targets (Fig. 49) were imaged at varied distances much larger than the hyperfocal distance. This allowed determination of the modulation transfer function (MTF) at different places in the FoV using a slanted edge method; note that the lighting was adjusted for each image, as uniform lighting of the large area proved challenging. Optical resolution (using the inverse of the frequency at which MTF falls below 0.3) is $0.31^{\circ }\pm 0.01^{\circ }$ near the center and $0.38^{\circ }\pm 0.01^{\circ }$ near the edge, about 3 pixels.

##### Neutral Density Coating

The inner diameter of the ND annulus is 71.7^∘^; the outer diameter of the annulus is 104.8^∘^; the annulus is centered $<2$ pixels from the center of the arrays. The Sun will be centered in the ND annulus when it is at an elevation angle of 46^∘^ for a level rover, and a normal transit of the baffle would take approximately 1.1 hours of Local Mean Solar Time (about 4100 s, compared to a Martian hour of 3699 s). For adverse tilts (>25^∘^ north, at 19^∘^ north latitude, at northern winter solstice) the Sun does not enter the annulus; however, the Sun typically spends at least an hour each morning and afternoon within the annulus.

A solar source was imaged within the central part of the FoV and within the ND annulus. In the central images, the shortest non-zero exposure led to saturation; however, 0-sec images allowed determination of the source brightness in instrument units. In the ND area, 15-sec exposures were used to measure the source while centered in the annulus, approximately on the diagonals of the FoV. The resulting measurement of attenuation was 4.997 ± 0.004 orders of magnitude. The ND area is also in an area that is 30–50% as sensitive as the image center (this varies due to the flat field), resulting in the ND area being near 5.4 orders of magnitude less sensitive than the center of the array.

### RDS Measurement Strategy and Operation Modes

The double electrical interface present in the RDS, one for the photodetectors and one for the SkyCam, provides the possibility to manage their operation independently and even capable of working in parallel.

RDS-DP is a low power and low data volume sensor designed to be activated along the sol, with a corresponding duty cycle compatible with the rover power budget. Two different commands can be sent to the photodiodes electronics: a) *the nominal acquisition command*, which performs a complete acquisition cycle to all RDS-DP channels. This command has an unique configuration parameter, setting the number of acquisitions per channel that are accumulated at a 100 ksps (kilo-samples per second) rate, and finally sent back to the ICU as telemetry; b) *the high gain command* that performs the same operation as the nominal command, but applying a higher gain to the optical channels, aiming at getting a better resolution in sunrises and sunsets, and so evaluating the offset of the channels during the night to estimate possible degradation.

As for RDS-SkyCam, in compliance with the overall mission data volume and power constraints allocated to MEDA, the nominal scenario states the acquisition of 7 images per sols (commanded through the execution of OTs, or direct commands from the rover computer). Typically, these daily images will be distributed to be acquired during sunrise, sunset, in the middle of the day, or when the Sun is blocked by the ND annulus mask. As mentioned, these images will let the team to (a) perform dust surveys, (b) look for clouds and characterize their motion; (c) cross-calibrate with other cameras; and (d) obtain cross-sky astronomical images. The latter may include images to derive night opacities from stellar fluxes, or to look for meteors.

There are two ways to command image acquisitions. On the one hand, through the so-called Observation Tables (OT), a program that is made in a specific MEDA-language, which runs internally in the instrument ICU, and that can be uploaded whenever necessarily. These OT acquisition commands will have manually configured exposure times, as the instrument doesn’t have the capability to automatically estimate the best exposure time according to lighting conditions.

On the other hand, direct commands from the rover computer can also trigger the acquisition of images on MEDA. In this case, the images can be acquire with predetermined exposure times, or automatically estimated on board through algorithms that are executed in the rover computer. This last mode is the so-called MEDA Auto-Expose acquisition.

In both case, the acquisition strategy consists of capturing two images in a row, one with an exposure time of 0 seconds (dark image), and other with the commanded exposure time. Both images will be stored in the instrument memory, and subsequently sent to the rover for automatic subtraction, thus generating a single $1024\times1024$ corrected image with the elimination of dark currents (after being stripped of the reference columns), which will be subsequently sent to Earth. Other modes of operation on the rover computer provide for the raw images to be sent to Earth, or different compression algorithms to minimize bandwidth.

Once on Earth, the images will be processed (converted into a readable format, radiometrically and geometrically calibrated, …), and then archived and published into the Planetary Data System for further use of the planetary scientific community.

### RDS Retrieval Products

Aerosol optical depth will be determined from SkyCam images from solar fluxes observed within the ND annulus, and reported through the mission in ASCII tables. As the images will be taken over a narrow range of solar zenith angles using a camera on which dust is expected to accumulate, the retrieval will be a multi-instrument effort. Mastcam-Z (Bell et al. 2020) opacities are expected to be calibrated like those from previous missions via Beer-Lambert-Bougher Law. From time to time through the mission, simultaneous measurements will be used for cross-calibration. Operationally, SkyCam is expected to be used to provide twice-daily measurements of opacity, while Mastcam-Z is expected to provide less time coverage, but add spectral coverage and cross-calibration to remove the effect of dust on RDS.

Multiple scattering discrete-ordinates radiative transfer modeling will be used to model the observed sky brightness observed by the RDS photodiodes and SkyCam. In this upward-looking geometry, the observed sky brightness comes from sunlight scattered by aerosols into the line-of-sight of each photodiode or SkyCam pixel, and thus depends on the basic properties of the scattering aerosols. The quantities to be retrieved using RDS observations are:

1. Column optical depth of dust and water ice aerosols.
2. Effective particle size for dust and water ice aerosols.
3. Column abundance of ozone gas.

Each of the above quantities will be retrieved as a function of local time, and as a function of season as the mission progresses. Additionally, constraints may be placed on the shape of the aerosols and the vertical distribution of aerosols at certain times of day when the geometry allows such analysis.

## Conclusions

This work describes the concepts behind the science intent, design, and development of what is actually a suite of instruments, several of them technically complex on their own. It has been an enormous international collaborative effort by many engineers and scientists from different institutions and agencies. The challenges that were confronted have led to technological achievements even before integration into a rover that, due to its volume, geometry, and the generated thermal environment, interacts with most of the environmental variables that MEDA will record. What has been learned in this process can be hopefully applicable to future missions and, additionally, return new science on a new location on Mars.

The team anticipates, and also hopes, that the science investigations outlined in the different sections of this manuscript are not the full story and readers can find in this document the tools and inspiration to enrich the science feasible with MEDA.

## List of Acronyms

ADC: Analog to Digital Converter
ARF: Angular Response Function
ASIC: Application-Specific Integrated Circuit
ATS: Air Temperature Sensor
BSS: Black Silver Smoke
CAB: Centro de Astrobiologia, Spain
CC: Contamination Control
CCD: Charge-Couple Device
CCN: Cloud Condensation Nuclei
CFD: Computational Fluid Dynamics model
CI: Color Index
CJ: Cold Junction
CM: Calibration Model
CO_2_: Carbon Dioxide
DHMR: Dry Heat Microbiological Reduction
DLR: German Aerospace Center
DN: Discrete Digital Number, RDS-SkyCam counter units
DP: Discrete Photodetector, RDS-DP
DREAMS: DREAMS Instrument onboard ExoMars 2016/Schiaparelli lander
EDL: Entry, Descent and Landing
EM: Engineering Model
EQM: Engineering Qualification Model
FM: Flight Model
FMI: Finnish Meteorological Institute, Finland
FoV: Field of View
FPGA: Field-Programmable Gate Array
FS: Flight Spare
G: Gas Thermal Conductance Estimation
GCM: General Circulation Model
GCR: Galactic Cosmic Rays
GL: Longitudinal Gas Thermal Conductance Estimation
GT: Transverse Gas Thermal Conductance Estimation
HCC: Worst Cold Case
HDRM: Hold-Down and Release Mechanism
HEPA: High Efficiency Particulate Air
HJ: Hot Junction
HP3: Heat Flow and Physical Properties Package onboard InSight
HS: Humidity Sensor
ICU: Instrument Control Unit
IF: Interferometric
INTA: Instituto Nacional de Técnica Aeroespacial, Spain
IR: Infrared
ISRU: In-Situ Resource Utilization
IUT: Instrument Under Test
JPL: Jet Propulsion Laboratory
L<x>: Level <x> requirements
LAT: Lateral
LW: Long Wave
M2020: Mars 2020 mission
MEDA: Mars Environmental Dynamics Analyzer
MEPAG: Mars Exploration Program Analysis Group
MER: Mars Exploration Rover
Mini-TES: Miniature Thermal Emission Spectrometer onboard MER rovers
MOXIE: Mars Oxygen In-Situ Resource Utilization Experiment
MSF: Mars Simulation Facility
MSL: Mars Science Laboratory mission
MTF: Modulation Transfer Function
MUPUS: Multi Purpose Sensor package onboard the Rosetta lander
ND: Neutral Density
OGSE: Optical Ground Support Equipment
OH: Optical Head
OT: Observation Table
P<x>: Pressure Sensor’s transducer <x>
PASLAB: Planetary Analog Simulation Laboratory
PCB: Printed Circuit Board
PDS: NASA’s Planetary Data System
PE: Processing Electronics
PFM: Proto-Flight Model
PHX: Phonenix mission
PP: Planetary Protection
Pr: Prandtl number
PRT: Platinum Resistance Thermometer
PS: Pressure Sensor
PTFE: Polytetrafluoroethylene (Teflon)
QM: Qualification Model
RAMP: RoverAvionics Mounting Panel
RCE: Perseverance’s Rover Compute Element
RDS: Radiation and Dust Sensor
Re: Reynolds number
REMS: Rover Environmental Monitoring Station
RH: Relative Humidity
RSM: Remote Sensing Mast
RTD: Resistance Temperature Detector
RTG: Radioisotope Thermoelectric Generator
SCS: Sampling and Caching System
SEB: Surface Energy Budget
SIS: Solar Irradiance Sensor
SKG: Strategic Knowledge Gap
SkyCam: Imager, RDS-SkyCam
SNR: Signal to Noise Ratio
SPASOLAB: Space Solar Cell Testing Laboratory at INTA, Spain
SRAM: Static Random Access Memory
STT: System Thermal Test
SW: Short Wave
TF: Transfer Function
TIRS: Thermal IR Sensor
TMM: Thermal Mathematical Model
TRF: Thermal Response Function
TS: Thermocouple Sensor
TWINS: Temperature and Wind for InSight
UV: Ultraviolet
VL: Viking Lander
WHC: Worst Hot Case
WS: Wind Sensor
